# Macrocyclic Donor–Acceptor Dyads Composed of a Perylene Bisimide Dye Surrounded by Oligothiophene Bridges

**DOI:** 10.1002/anie.202113598

**Published:** 2021-11-23

**Authors:** Kevin Bold, Matthias Stolte, Kazutaka Shoyama, Marco Holzapfel, Alexander Schmiedel, Christoph Lambert, Frank Würthner

**Affiliations:** ^1^ Institut für Organische Chemie Universität Würzburg Am Hubland 97074 Würzburg Germany; ^2^ Center for Nanosystems Chemistry (CNC) Universität Würzburg Theodor-Boveri-Weg 97074 Würzburg Germany

**Keywords:** donor–acceptor dyads, macrocycles, oligothiophenes, perylene bisimide, photoinduced electron transfer

## Abstract

Two macrocyclic architectures comprising oligothiophene strands that connect the imide positions of a perylene bisimide (PBI) dye have been synthesized via a platinum‐mediated cross‐coupling strategy. The crystal structure of the double bridged PBI reveals all *syn*‐arranged thiophene units that completely enclose the planar PBI chromophore via a 12‐membered macrocycle. The target structures were characterized by steady‐state UV/Vis absorption, fluorescence and transient absorption spectroscopy, as well as cyclic and differential pulse voltammetry. Both donor–acceptor dyads show ultrafast Förster Resonance Energy Transfer and photoinduced electron transfer, thereby leading to extremely low fluorescence quantum yields even in the lowest polarity cyclohexane solvent.

## Graphical abstract

The synthesis and properties of a perylene bisimide dye bridged by one or two quinquethiophene units are described. The compounds show panchromatic light absorption in the UV/Vis spectrum, multiple reversible redox processes, and a highly efficient charge separation by photoinduced electron transfer.

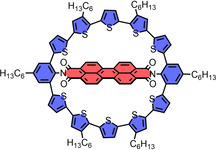

## Introduction

Cyclic molecular structures have always attracted chemists for a variety of reasons. These include purely aesthetic reasons,^[1]^ the suitability of macrocycles as well pre‐organized supramolecular host^[2]^ and the usefulness of π‐conjugated (macro‐)cycles to shed light on aromaticity^[3]^ or for enabling special physical properties^[4]^ of interest. Emanating from the latter research field, the fascinating classes of cyclo[*n*]oligothiophenes,^[5]^ cyclo[*n*]paraphenylenes,^[6]^ carbon nanobelts^[7]^ as well as porphyrin nanorings^[8]^ have been realized in the recent years. Some of them, such as Bäuerle's cyclo[*n*]oligothiophenes or Osuka's and Anderson's porphyrin nanorings, also exhibit fascinating properties, that is, the formation of polaron pairs (biradicals) or the switching between aromatic and antiaromatic properties upon oxidation or photoexcitation, respectively.[^3e^, ^4c^, ^9^] By taking a look onto the light‐harvesting complex 1 (LHC 1) of purple bacteria we may identify another roadmap for future research on macrocyclic functional π‐scaffolds.^[10]^ In this complex nature organizes a ring of 32 bacteriochlorophyll (BChl) *a* molecules in an elliptical ring structure around the reaction center with its special pair of BChl *b* molecules, thereby encoding the desirable functionality of light harvesting, energy transfer and electron transfer required for the primary steps of photosynthesis.^[11]^ This example from nature may inspire chemists to further develop the chemistry on π‐conjugated macrocycles consisting of a sequence of identical building blocks such as phenylene, thiophene, porphyrin, etc. towards dyads^[12]^ including a second functional dye to realize a broader variety of functional properties. In this Research Article we pursue along this approach starting from the pioneering work of Bäuerle on cyclo[*n*]oligothiophenes.^[5]^ Like their linear counterparts, cyclo[*n*]oligothiophenes are electron‐rich molecules belonging to the class of organic semiconductors.^[13]^ By combining cyclo[*n*]oligothiophenes with electron‐poor perylene bisimides (PBIs),^[14]^ we envisioned to accomplish molecular dyads with the function of panchromatic light absorption and efficient charge separation on the nanoscale, similar as at the donor–acceptor interface of a photovoltaic bulk heterojunction.

## Results and Discussion

### Synthesis and Structural Characterization

**Scheme 1:**
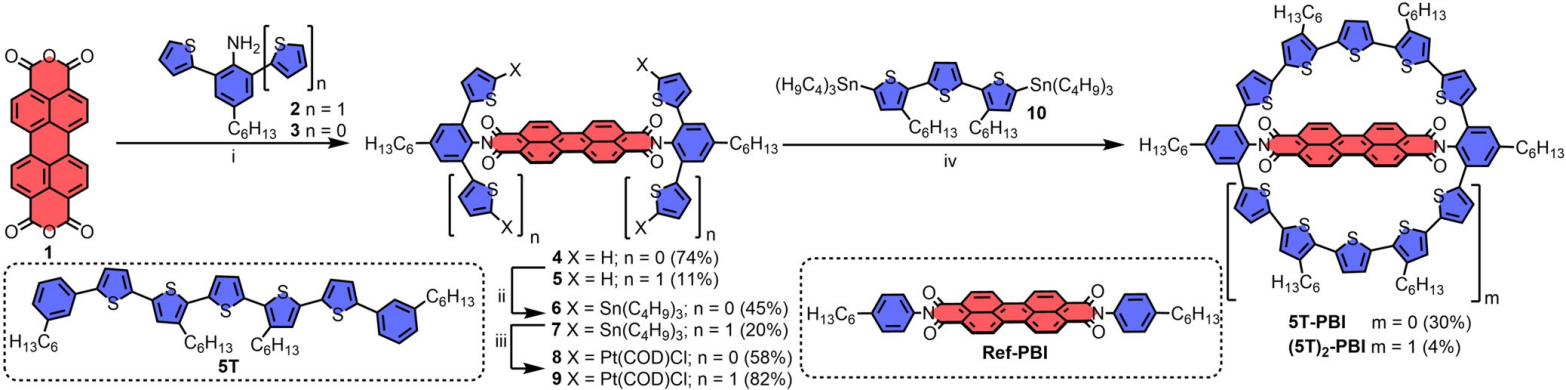
Synthesis of the macrocyclic architectures **(5T)_2_‐PBI** and **5T‐PBI**. Reagents and conditions: i) Zn(OAc)_2_, imidazole, microwave irradiation. ii) Sn(C_4_H_9_)_3_Cl, *n*‐BuLi, THF, r.t., overnight. iii) Pt(COD)Cl_2_, toluene, 95 °C. iv) Toluene, 75 °C, overnight, then dppf, CH_2_Cl_2_, r.t., 6 h, then *m*‐xylene, 120 °C, overnight. Dppf=1,1′‐Ferrocenediyl‐bis(diphenylphosphine); COD=1,5‐Cyclooctadiene.

Our synthesis of macrocycles **5T‐PBI** and **(5T)_2_‐PBI** started with the imidization of perylene bisanhydride **1** with sterically hindered amines^[15]^ **2** and **3** which could only be accomplished using microwave irradiation as well as elevated temperatures yielding PBIs **4** and **5** (Scheme 1). Subsequently, twofold and fourfold stannylation of the free thiophene α‐positions was conducted using excess of deprotonation agent *n*‐BuLi and tin source Sn(C_4_H_9_)_3_Cl. Upon Sn–Pt exchange of **6** and **7** with Pt(COD)Cl_2_ the precursors **8** and **9** could be obtained. At this point, it should be noted that the compounds **4**, **6** and **8** prevail as mostly inseparable *syn*/*anti* rotameric mixtures in solution. In case of **5T‐PBI**, precursor molecules **8** exhibiting a *syn*‐conformation are a prerequisite for the desired macrocyclization to proceed and accordingly elevated temperatures in order to overcome the calculated inversion barrier of 114 kJ mol^−1^ (for details see Supporting Information, SI) were applied for the final reaction.^[16]^ Inspired by other successful macrocyclization protocols utilizing this method in literature,^[17]^ especially the formation of cyclo‐*para*phenylenes,^[18]^ the macrocyclization of bay‐bridged PBI‐thiophene compounds^[19]^ and the synthesis of giant cyclic oligothiophenes,^[5b]^ we decided to apply this three step platinum‐mediated cross‐coupling reaction cascade to obtain target molecular dyads **5T‐PBI** and **(5T)_2_‐PBI** in yields of 30 % and 4 %, respectively (Scheme 1; for details see SI). This decrease in yields can be explained by four vs. two couplings statistics and the enhanced strain energy of **(5T)_2_‐PBI** in comparison to **5T‐PBI** (vide infra). Target molecules **5T‐PBI** and **(5T)_2_‐PBI** were characterized by nuclear magnetic resonance (NMR) spectroscopy and high‐resolution mass spectrometry (HRMS). In comparison to their linear phenyl end‐capped α‐quinquethiophene counterpart **5T** (Scheme 1)^[20]^ the thiophene units in the macrocyclic bridges in **5T‐PBI** and **(5T)_2_‐PBI** adopt a more rigid all‐*syn* configuration in solution. Two doublet and two singlet proton signals in the aromatic region of the ^1^H NMR spectrum for both constrained oligothiophenes confirm the high symmetry (Figure S1). Interestingly, both sets of oligothiophene signals for **5T‐PBI** and **(5T)_2_‐PBI** barely differ in the chemical shift suggesting a very similar chemical proton environment, which is also true for the PBI subunit proton signals when compared to **Ref‐PBI** (Scheme 1).

**Figure 1:**
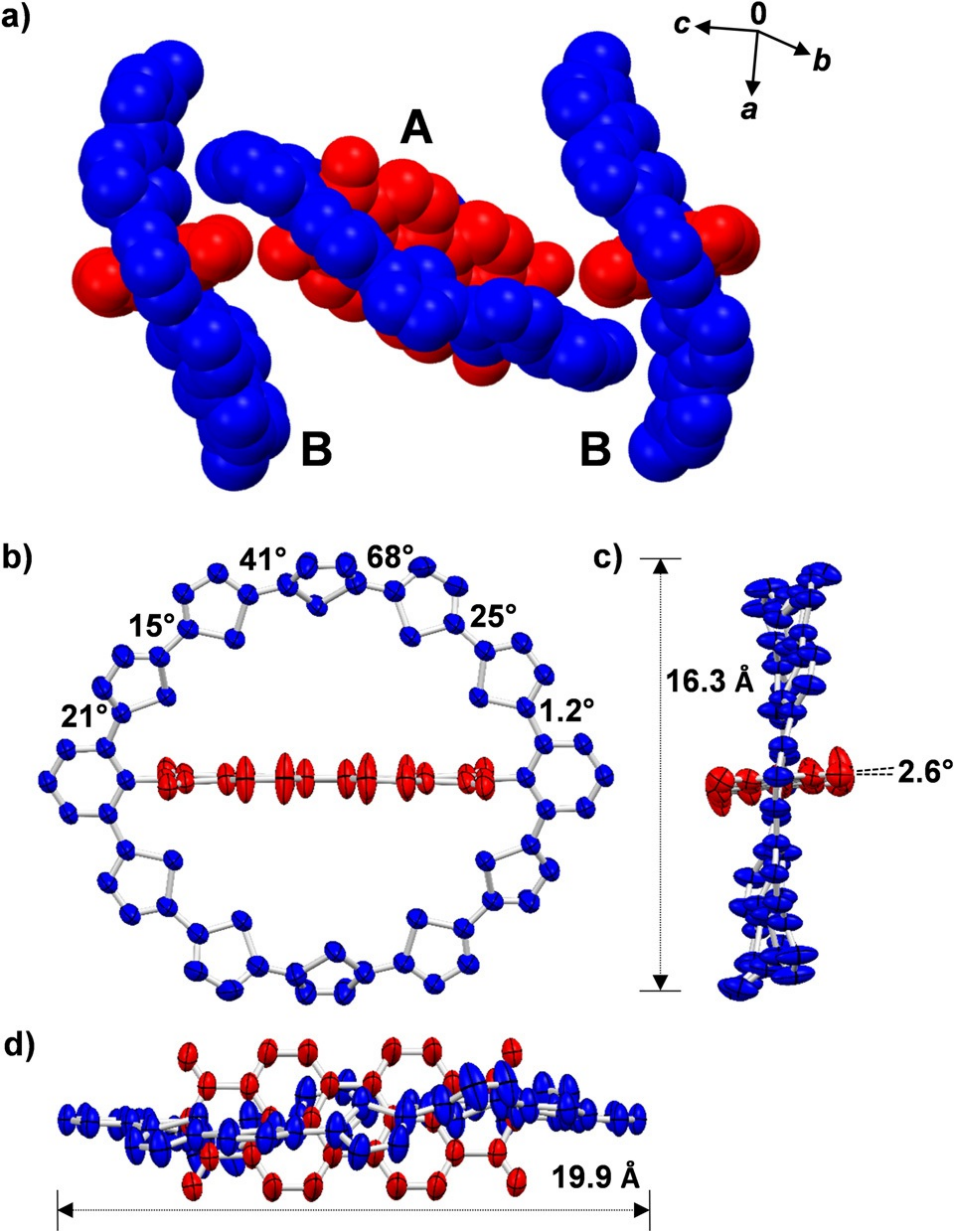
Crystal structure of **(5T)_2_‐PBI** with ellipsoids set to 50 % probability and PBI colored in red and macrocycle in blue.^[30]^ a) Space‐filling model of the unit cell consisting of three molecules with structures A and B and their molecular packing in the crystal structure. b) Front, c) side and d) top view onto the molecule A of **(5T)_2_‐PBI** with crystallographic inversion center. The hydrogen atoms, solvent molecules as well as the alkyl chains are omitted for clarity.

For an unambiguous structural proof for the 12‐membered macrocycle‐formation as well as PBI enclosure, single crystals suitable for X‐ray crystallography could be grown by slow vapor diffusion of methanol into a solution of **(5T)_2_‐PBI** in chlorobenzene. While the small set of signals in the aromatic region of the ^1^H NMR (SI) suggested a very high degree of symmetry (*D*_2*h*_) in solution, contrarily in the crystal of **(5T)_2_‐PBI** two different manifestations of the molecule are present, presumably due to packing effects in the solid state. One motif has a crystallographic inversion center (molecule A in Figure 1 a), whereas the other (molecule B in Figure 1 a) shows rather deformed oligothiophene bridges. Two of these non‐symmetric structures B flank one molecule A with high symmetry in the unit cell (Figure 1 a). The attached aliphatic chains are not depicted in Figure 1 but are included in Figure S2 in the SI.

Figure 1 b–d provide further analysis for molecule A whilst molecule B, embedded solvent molecules and structural disorder in the unit cell is analyzed in Figure S3 in the SI. In the side and top view of the molecule (Figure 1 c and d) the thiophene chain describes an S‐shape with respect to the PBI subunit. As proven by the crystal structure as well as corroborated from DFT calculations (Figure S5) both oligothiophene bridging units show an overall highly twisted structure with neighboring thiophene units rotated from 15° to 68° (Figure 1 b). These values are indeed larger than those observed for linear α‐oligothiophene chains^[21]^ and similarly sized cyclic oligothiophenes.[^9^, ^22^] Interestingly, the central thiophene units of each bridge show the highest torsion of the ten subunits, thereby interrupting the π‐conjugation. The oligothiophene ring with an outer diameter of 16.3 Å adapts an all‐*syn* conformation with all sulphur atoms pointing towards the central PBI chromophore. The averaged interatomic S–S distance amounts to 3.37 Å which is 9 % below the sum of their van der Waals radii (3.70 Å). Together with the slightly extended interring thiophene‐thiophene bond lengths (1.453(3) Å to 1.477(3) Å) the above discussed factors add up to a strained macrocycle which also leads to electronic consequences (vide infra). Due to the fact that the central PBI chromophore only exhibits a small twist between the two naphthalene subunits of about 3° it can be concluded that the oligothiophene bridges compensate for the major part of the macrocyclic strain energy of 30.6 kJ mol^−1^ for **(5T)_2_‐PBI** which is slightly more than two‐fold higher as for **5T‐PBI** (13.9 kJ mol^−1^, for details see the Supporting Information). In comparison to similarly sized macrocycles such as [12]cycloparaphenylene (117 kJ mol^−1^)^[6]^ or α‐cyclo[12]oligothiophene (7.5 kJ mol^−1^)^[5a]^ the macrocycle (**5T)_2_‐PBI** can be considered as moderately strained

### Redox Properties

**Figure 2:**
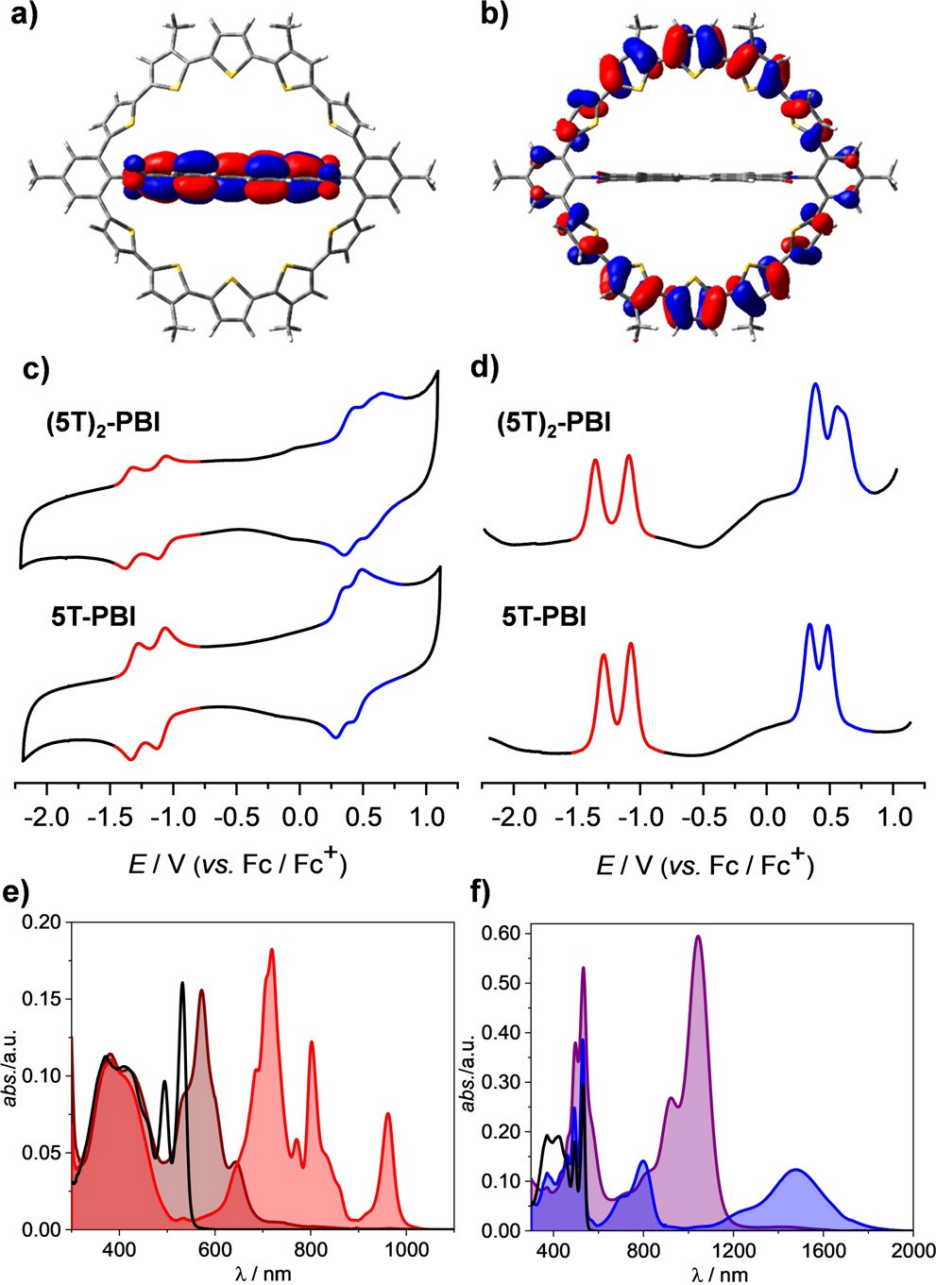
a) LUMO and b) HOMO of **(5T)_2_‐PBI** based on geometry optimized structures from DFT calculations. The quantum mechanics calculations were carried out on the level of B3LYP density functional with the 6‐31G(d) basis set as implemented in Gaussian 16. c) CV and d) DPV measurements of **5T‐PBI** and **(5T)_2_‐PBI** as well as UV/Vis/NIR absorption spectra of **5T‐PBI** (black line) upon electrochemical reduction to e) **5T‐PBI^.−^** (red line), **5T‐PBI^2−^** (maroon line) and electrochemical oxidation to f) **5T^.+^‐PBI** (blue line) and **5T^2+^‐PBI** (violet line) in CH_2_Cl_2_ solutions with Bu_4_NPF_6_ at room temperature (*c*_0_=10^−4^ M).

Both oligothiophene‐bridged macrocycles were electrochemically characterized by cyclic voltammetry (CV), differential pulse voltammetry (DPV) and spectroelectrochemistry (SEC, for **5T‐PBI**) in dichloromethane (CH_2_Cl_2_) with Bu_4_NPF_6_ as supporting electrolyte (Figure 2). In the CV reversibility of all involved redox processes can be monitored.

Two reversible reduction waves for both structures, **5T‐PBI** and **(5T)_2_‐PBI**, mark the formation of an anionic (*E*_red,1_=−1.07 V/−1.09 V, Table 1) and dianionic (*E*_red,2_=−1.29 V/−1.35 V) PBI chromophore, respectively. Compared to **Ref‐PBI** (*E*_red,1_=−0.99 V/*E*_red,2_=−1.19 V, Figure S7) these reduction events are at about 0.1 V more negative potential which might be due to a macrocyclic ring‐strain induced destabilization of the anionic states that are characterized by a reduced length of the PBI unit along its long axis.^[23]^ The localization and shapes of the lowest unoccupied and highest occupied molecular orbital (LUMO/HOMO) of the neutral **(5T)_2_‐PBI** (Figure 2 a,b) suggest very little electronic communication between electron donor oligothiophene chain and PBI acceptor segment which is also true for **5T‐PBI** (Figure S9). Due to the nodes which are located at the imide nitrogen atoms, conjugation of PBI to the residues is interrupted. Likewise, due to the *meta*‐connectivity, the two oligothiophene units are rather independent π‐conjugated units. This interpretation is supported by the almost invariant redox properties as well as by time‐dependent density functional theory (TDDFT) calculations (Table S2), where the oscillator strengths for HOMO–LUMO transitions were determined to be close to zero, which agrees well with the observed UV/Vis absorption spectra (vide infra), that show now charge transfer (CT) band.

**Table 1:** Photophysical and electrochemical properties of macrocycles **5T‐PBI**, **(5T)_2_‐PBI** as well as **Ref‐PBI** and **5T** ^[20]^ in CH_2_Cl_2_ at room temperature.

	*λ*_max_^[a]^ [nm]	*ϵ*_max_^[a]^ [M^−1^ cm^−1^]	*E*_ox,1_^[b]^ [V]	*E*_ox,2_^[b]^ [V]	*E*_red,1_^[b]^ [V]	*E*_red,2_^[b]^ [V]
**Ref‐PBI**	526	72 100	–^[c]^	–^[c]^	−0.99	−1.19
**5T** ^[20]^	422	53 500	0.41	0.59	–^[c]^	–^[c]^
**5T‐PBI**	531	64 800	0.34	0.48	−1.07	−1.29
**(5T)_2_‐PBI**	536	54 000	0.39	0.56	−1.09	−1.35

[a] *c*_0_=10^−6^ M. [b] *c*_0_=10^−4^  M, Bu_4_NPF_6_ was used as electrolyte; all half‐wave potentials are referenced against the ferrocenium/ferrocene (Fc^+^/Fc) redox couple. [c] Not observed.

While all reduction waves correspond to one‐electron processes of the PBI subunit, the waves for the oxidations of both macrocycles have to be differentiated. For **5T‐PBI** the oxidation waves also reflect one‐electron processes (*E*_ox,1_=+0.34 V and *E*_ox,2_=+0.48 V), whereas two bridge moieties are simultaneously oxidized in **(5T)_2_‐PBI**. Accordingly, the two oxidation potentials *E*_ox,1_=+0.39 V and *E*_ox,2_=+0.56 V each represent the transfer of two electrons (Figure S8). Therefore, due to the installation of a second semi‐oligothiophene circle in **(5T)_2_‐PBI** tri‐and tetracationic states are accessible. With a better resolution of the peaks in DPV (Figure 2 d) a broadening of the second oxidation wave of **(5T)_2_‐PBI** can be observed. Thus, upon formation of (**(5T)_2_‐PBI**)^2+^ the increased quinoidal character of the oligothiophene chain calls for an almost stepwise oxidation to the tricationic (**(5T)_2_‐PBI**)^3+^ and tetracationic (**(5T)_2_‐PBI**)^4+^ state due to severe Coulomb interactions between the charges in both bridging units. This can be interpreted as an electronic intramolecular cation‐cation communication between the bridged thiophene chains.^[24]^ The described oxidation features of **(5T)_2_‐PBI** do not resemble those of structurally closely related and fully conjugated α‐cyclo[10]thiophene of Bäuerle and co‐workers^[9]^ where four reversible oxidation waves with a very low energy first oxidation potential of 0.03 V were found but rather the cyclophane type alkylene bridged quinquethiophene structures of Sakai et al.^[24c]^ In accordance with the observed electronic phenomena for **(5T)_2_‐PBI** also in this structure both oligothiophenes show two reversible oxidation events of which the high energy oxidation process splits in to two CV waves. Thus, the multi‐oxidation process for **(5T)_2_‐PBI** involving four electrons presumably occurs stepwise. Furthermore, the oxidations are in the same range compared to the linear all‐*anti* oligothiophene analogue **5T** (*E*_ox,1_=+0.41 V/*E*_ox,2_=+0.59 V).^[20]^ Besides small contortion related reduction or oxidation potential changes of about 0.1 V and the above mentioned cation‐cation interaction the electronic properties of **5T‐PBI** and **(5T)_2_‐PBI** are nearly a sum of the reference structures **Ref‐PBI** and **5T**. Thus, as envisioned, by connecting both PBI's imide positions with oligothiophene chains a covalently linked donor‐acceptor dyad with independent subunits has been be achieved.

This lack of electronic communication between donor and acceptor is further illustrated in the SEC (Figures 2 e and f). Upon electrochemical reduction of **5T‐PBI** the PBI segment is independently reduced and spectral changes occur only for this part of the spectrum (450–550 nm) while the oligothiophene region (300–450 nm) mainly remains unchanged. In contrast, electrochemical oxidation leads to the selective emerging of oligothiophene cation and dication related bands. The spectral signature for anion and dianion of **5T‐PBI** depicted in Figure 2 e compares well to similar literature known bay‐unsubstituted reference PBI structures.^[23]^ Typical spectral PBI signatures comprising three sharp bands at 719 nm, 802 nm and 962 nm corresponding to the anion **5T‐PBI^.−^** (Figure 2 e, red line) which decrease upon further reduction and give rise of a band at 572 nm (maroon line) belonging to the dianion **5T‐PBI^2−^**. Onefold oxidation of **5T‐PBI** leads to the simultaneous formation of two peaks at 795 nm and 1480 nm indicating the formation of the cationic species **5T^.+^‐PBI** (Figure 2 f, blue line). Further oxidation to the dicationic species **5T^2+^‐PBI** causes submerging of the afore mentioned cationic signals and emerging of a strong signal at 1043 nm (Figure 2 f, violet line). Also the cationic and dicationic band shapes (Figure 2 f) look much alike linear quinquethiophene analogues in similar solvents[^9^, ^25^] but both respective near‐IR peaks (1480 nm and 1043 nm) undergo a red shift of about 200 nm (1031 cm^−1^ and 2108 cm^−1^) due to the two additional phenyl units and the consequent enhanced conjugation length. This band shape similarity of both macrocyclic subunits in comparison with the free donor and acceptor analogues further underlines the fact that both π‐systems have to be regarded as electronically decoupled.

### Optical Properties

**Figure 3:**
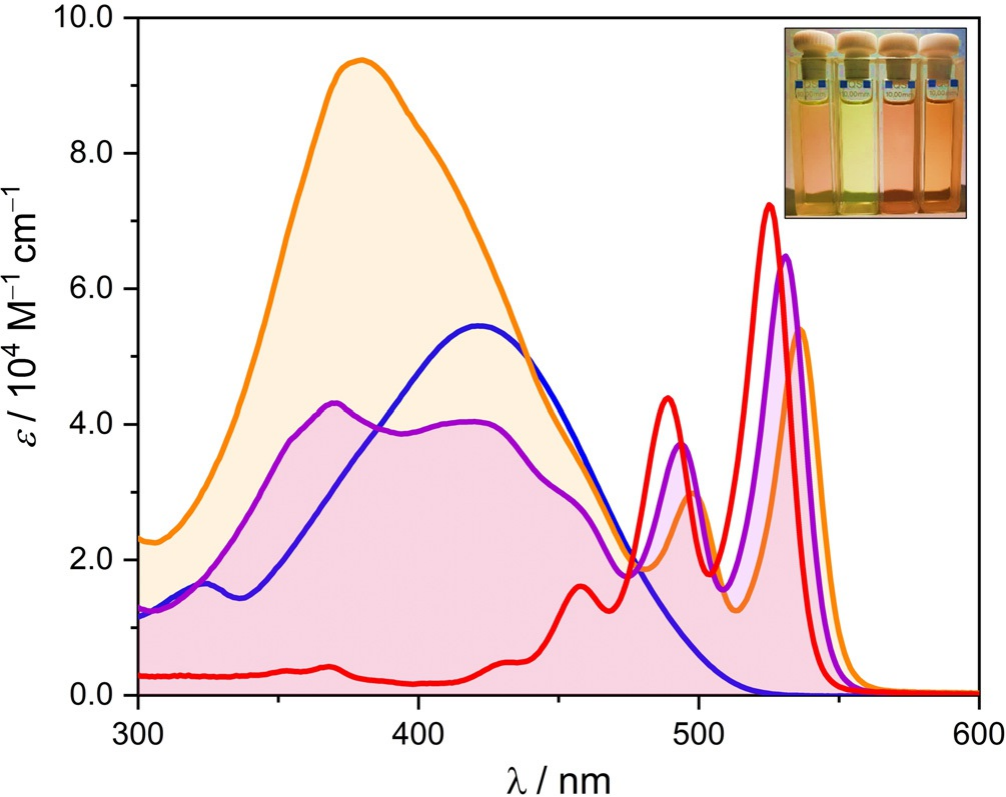
UV/Vis absorption spectra (*c*_0_=10^−6^ M) of **5T‐PBI** (purple line), **(5T)_2_‐PBI** (orange line), **5T** (blue line) and **Ref‐PBI** (red line) in CH_2_Cl_2_ at room temperature. Inset: Photograph of **Ref‐PBI**, **5T**, **5T‐PBI** and **(5T)_2_‐PBI** (from left to right) in CH_2_Cl_2_.

Spectroscopic investigations of **5T‐PBI** and **(5T)_2_‐PBI** were performed in CH_2_Cl_2_ (Figure 3). The UV/Vis spectra show a superposition of a high energy oligothiophene absorption below 450 nm as well as the characteristic S_0_–S_1_ transition of the PBI at around 530 nm with well‐resolved vibronic progression. In contrast to **Ref‐PBI** (*λ*_max_=526 nm, *ϵ*_max_=72 100 M^−1^ cm^−1^) the absorption maxima of the macrocyclic PBIs in **5T‐PBI** and **(5T)_2_‐PBI** experience a slight bathochromic shift of 6 nm (215 cm^−1^) and 11 nm (390 cm^−1^) with an additional decrease of their respective molar extinction coefficients (*ϵ*_max_, Table 1). Similar red‐shifted absorbances were recently reported by Nuckolls and co‐workers for highly bent PBIs.^[26]^

Compared to the structureless absorption of the phenyl end‐capped α‐oligothiophene **5T** counterpart's^[20]^ π‐π* transition (Figure 3), rigidification of the oligothiophene bridge within the *syn*‐macrocyclic architectures leads to distinct vibronic fine structures in the higher energy region of the UV/Vis spectrum for **5T‐PBI** and **(5T)_2_‐PBI**. While only one oligothiophene absorption maximum can be found for the latter macrocycle (*λ*_Thio_=380 nm, *ϵ*_max, Thio_=93 900 M^−1^ cm^−1^) the monobridged analogue shows two distinct maxima at 369 nm and 420 nm, respectively. According to the absorption spectrum of **(5T)_2_‐PBI** both bridges are not in full conjugation but rather have to be considered as photophysically independent. Further, the spectral signatures with most intense absorption bands at rather high energy indicate a loss in π‐conjugation due to the rotational twists of the oligothiophene backbone. While **Ref‐PBI** exhibits fluorescence quantum yields (*Φ*_fl_) close to unity in many solvents^[27]^ and **5T** is a strong emitter with 53 %^[20]^ as well, both donor–acceptor dyads **5T‐PBI** and **(5T)_2_‐PBI** show no emission in polar solvents such as dichloromethane (for a comparison of reference structures and macrocycles see Figure S10), which favor photoinduced electron transfer (PET, vide infra).

Even in cyclohexane only extremely low *Φ*_fl_ below 0.1 % were determined (Table S3, Figure S11). Due to the distinguishable spectral regions of the oligothiophene bridge donor and PBI core acceptor both subunits can be excited separately. Upon selective excitation of the PBI acceptor at 480 nm in cyclohexane, typical PBI emission with mirror‐image behavior can be observed for **5T‐PBI**, whereas **(5T)_2_‐PBI** shows similar but broadened vibronic patterns (Figure S11). Donor chain excitation at 340 nm and 310 nm for both macrocycles **5T‐PBI** and **(5T)_2_‐PBI** affords likewise a PBI emission which is attributed to a fast Förster Resonance Energy Transfer to the PBI acceptor unit.

### Transient Absorption Spectroscopy

**Figure 4:**
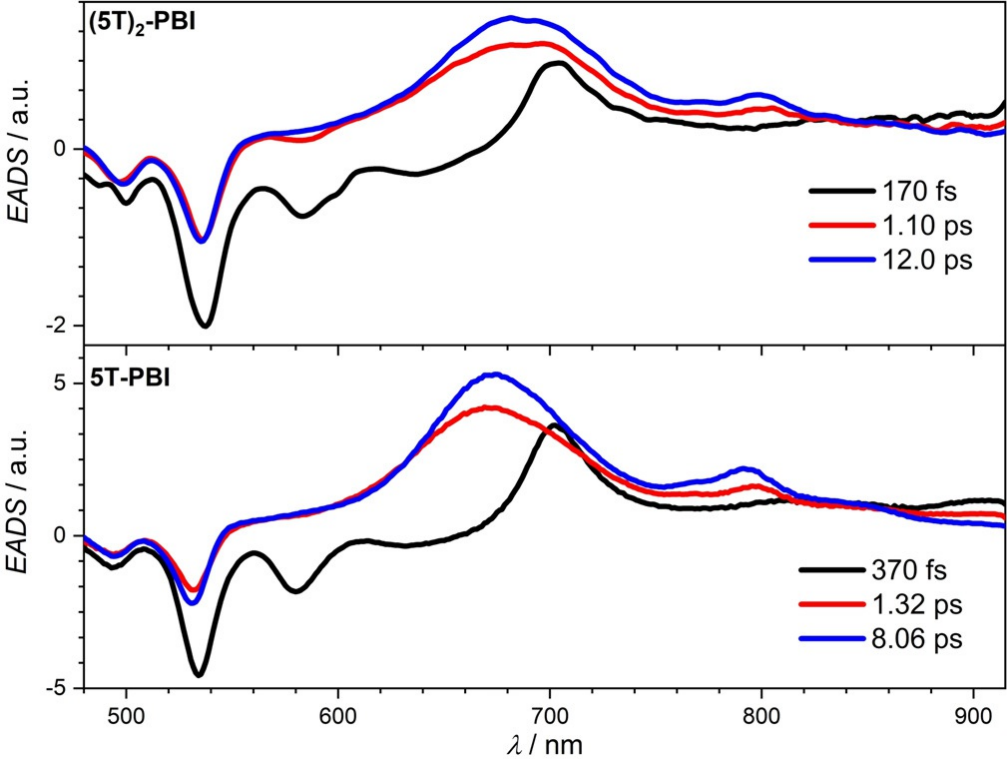
Evolution associated difference spectra (EADS) and lifetimes from a global fit analysis of the transient spectra of macrocycles **5T‐PBI** and **(5T)_2_‐PBI** obtained by excitation at 530 nm in CH_2_Cl_2_ (*c*_0_=10^−4^ M) at room temperature.

For a full understanding of the excited state dynamics, transient absorption (TA) spectra were measured upon excitation at 530 nm in CH_2_Cl_2_ (Figures S12 and S13). The thereof derived evolution associated difference spectra (EADS) reveal a similar behavior for **5T‐PBI** and **(5T)_2_‐PBI** (Figure 4). Thus, with the rise time of the IRF (instrument response function <ca. 80 fs) a ground state bleaching (GSB) at 530 nm and stimulated emission (SE) at 580 nm is observed, confirming the exclusive excitation of the PBI chromophore in the macrocycles. These spectra of the PBI's S_1_ state are followed by two subsequent similar TA spectra that show a superposition of the PBI radical anion and oligothiophene radical cation covering the spectral range from 500 nm to 850 nm (Figure 2 e and f). This is best viewed by comparison of the EADS with a radical anion and cation spectra received from SEC (Figure S14). The first EADS is assigned to a hot state that converts to the cold state by molecular and solvent relaxation within ca. 1 ps. The ultrafast population of this cold charge transfer state consisting of a PBI radical anion and oligothiophene radical cation is followed by a fast charge recombination (Figure 4) within 8 ps for the singly bridged and 12 ps for the doubly bridged derivative, respectively. The species giving rise to the first EADS possess lifetimes of 0.2 ps and 0.4 ps, respectively, thus are limited by the charge separation process with *k*_CS_=(0.4 ps)^−1^ and (0.2 ps)^−1^ for **5T‐PBI** and **(5T)_2_‐PBI**, respectively. Thus, the rate of the **(5T)_2_‐PBI**’s charge separation is two times faster which is presumably due to the second oligothiophene bridge which increases the charge separation probability and therefore also the rate. In closely related but less rigid and non‐cyclic PBI dyads functionalized with oligothiophenes the charge separation process of a PBI‐octithiophene dyad^[28]^ takes up to 13 times longer than for the macrocycles **5T‐PBI** and **(5T)_2_‐PBI**. To the best of our knowledge, the unique cyclic molecules **5T‐PBI** and **(5T)_2_‐PBI** allow for one of the fastest PET in an imide functionalized PBI‐oligothiophene dyad known in literature which is probably due the rigid structure and the extremely close proximity of donor and acceptor subunits.^[29]^

## Conclusion

In summary we synthesized two novel types of dyads comprising a central accepting PBI core which is bridged by one or two oligothiophene donor chains. Single crystal X‐ray analysis provided unambiguous proof for the complete enclosure of the PBI acceptor by the 12‐membered donor macrocycle. Rigidification and bending of each quinquethiophene semicircle leads to distinct high energy bands in the absorption spectra of both macrocycles in comparison to the linear phenyl‐end‐capped oligothiophene analogue. The imide functionalization of the PBI with oligothiophene(s) ensures the intended electronic and photophysical independency of the PBI and oligothiophene units. Upon photo‐excitation both macrocyclic dyads showed an ultrafast photoinduced electron transfer from the oligothiophene to the perylene bisimide subunit to afford a charge separated state for which both the radical anionic PBI and the radical cationic oligothiophene could be assigned by the comparison of the transient absorption spectra with those obtained by spectroelectrochemistry.

## Conflict of interest

The authors declare no conflict of interest.

## Supporting information

As a service to our authors and readers, this journal provides supporting information supplied by the authors. Such materials are peer reviewed and may be re‐organized for online delivery, but are not copy‐edited or typeset. Technical support issues arising from supporting information (other than missing files) should be addressed to the authors.

Supporting Information

Supporting Information
