# Supplementary material for: Macrocyclic Donor–Acceptor Dyads Composed of a Perylene Bisimide Dye Surrounded by Oligothiophene Bridges
Source: Angew Chem Int Ed Engl. 2021 Nov 23;61(1):e202113598. doi: 10.1002/anie.202113598 (PMC9299635; doi:10.1002/anie.202113598)

checkCIF/PLATON report

You have not supplied any structure factors. As a result the full set of tests cannot be run.

THIS REPORT IS FOR GUIDANCE ONLY. IF USED AS PART OF A REVIEW PROCEDURE FOR PUBLICATION, IT SHOULD NOT REPLACE THE EXPERTISE OF AN EXPERIENCED CRYSTALLOGRAPHIC REFEREE.

No syntax errors found. CIF dictionary Interpreting this report

Datablock: KB138m12_a

```
Bond precision:  C-C = 0.0046 A          Wavelength=0.61992

Cell:          a=15.385(10)      b=17.342(3)     c=31.216(5)
               alpha=74.973(5)   beta=89.82(2)   gamma=85.158(16)
Temperature:   100 K
```

	Calculated	Reported
Volume	8014(6)	8014(5)
Space group	P -1	P -1
Hall group	-P 1	-P 1
Moiety formula	C112.01 H106.03 N2 O4 S10, 1.438(C6 H5 Cl)	C112 H106 N2 O4 S10, 1.438(C6 H5 Cl)
Sum formula	C120.64 H113.21 Cl1.44 N2 O4 S10	C120.64 H113.21 Cl1.44 N2 O4 S10
Mr	2026.55	2026.55
Dx,g cm-3	1.260	1.260
Z	3	3
Mu (mm-1)	0.203	0.203
F000	3202.4	3202.4
F000’	3206.40	
h,k,lmax	23,25,46	22,25,46
Nref	56309	43791
Tmin,Tmax	0.980,0.980	
Tmin’	0.980	

```
Correction method= Not given

Data completeness= 0.778          Theta(max)= 27.653

R(reflections)= 0.0848( 26483)    wR2(reflections)= 0.3036( 43791)

S = 1.109                   Npar= 2852
```

The following ALERTS were generated. Each ALERT has the format
test-name_ALERT_alert-type_alert-level.
Click on the hyperlinks for more details of the test.

Alert level C

```
PLAT077_ALERT_4_C Unitcell Contains Non-integer Number of Atoms ..     Please Check
PLAT084_ALERT_3_C High wR2 Value (i.e. > 0.25) ...................       0.30 Report
PLAT213_ALERT_2_C Atom C88A_1          has ADP max/min Ratio .....        3.1 prolat
PLAT213_ALERT_2_C Atom C312_1          has ADP max/min Ratio .....        3.6 prolat
PLAT220_ALERT_2_C NonSolvent   Resd 1  C   Ueq(max)/Ueq(min) Range        5.9 Ratio
PLAT220_ALERT_2_C NonSolvent   Resd 2  C   Ueq(max)/Ueq(min) Range        3.8 Ratio
PLAT222_ALERT_3_C NonSolvent Resd 1  H   Uiso(max)/Uiso(min) Range        6.3 Ratio
PLAT241_ALERT_2_C High   'MainMol' Ueq as Compared to Neighbors of      C67_1 Check
PLAT241_ALERT_2_C High   'MainMol' Ueq as Compared to Neighbors of      C74_1 Check
PLAT241_ALERT_2_C High   'MainMol' Ueq as Compared to Neighbors of       S5_2 Check
PLAT241_ALERT_2_C High   'MainMol' Ueq as Compared to Neighbors of       C1_2 Check
PLAT241_ALERT_2_C High   'MainMol' Ueq as Compared to Neighbors of       C2_2 Check
PLAT241_ALERT_2_C High   'MainMol' Ueq as Compared to Neighbors of      C50_2 Check
PLAT242_ALERT_2_C Low    'MainMol' Ueq as Compared to Neighbors of      C10_2 Check
PLAT242_ALERT_2_C Low    'MainMol' Ueq as Compared to Neighbors of      C56_2 Check
PLAT250_ALERT_2_C Large U3/U1 Ratio for Average U(i,j) Tensor ....        2.2 Note
PLAT260_ALERT_2_C Large Average Ueq of Residue Including      S1_1      0.115 Check
PLAT260_ALERT_2_C Large Average Ueq of Residue Including      S1_2      0.115 Check
PLAT340_ALERT_3_C Low Bond Precision on  C-C Bonds ...............    0.00459 Ang.
PLAT410_ALERT_2_C Short Intra H...H Contact  H29_2    ..H55_2    .       1.97 Ang.
                                                1-x,1-y,1-z  =      2_666 Check
PLAT721_ALERT_1_C Bond    Calc    1.41(11), Rep     1.39000 Dev...       0.02 Ang.
              C3_115   -C4_115          1_555   1_555 ........     #  879 Check
PLAT723_ALERT_1_C Torsion Calc      -2(11), Rep        0.00 Dev...       2.00 Sigma
              C1_1-C2_1-C3_1-C4_1  1_555  1_555  1_555  1_555      #  759 Check
PLAT723_ALERT_1_C Torsion Calc       2(13), Rep        0.00 Dev...       2.00 Sigma
              C2_1-C3_1-C4_1-C5_1  1_555  1_555  1_555  1_555      #  760 Check
```

Alert level G

```
FORMU01_ALERT_1_G  There is a discrepancy between the atom counts in the
            _chemical_formula_sum and _chemical_formula_moiety. This is
            usually due to the moiety formula being in the wrong format.
            Atom count from _chemical_formula_sum:   C120.64 H113.21 Cl1.44 N2 O4
            Atom count from _chemical_formula_moiety:C120.6279 H113.19 Cl1.438 N2
ABSMU01_ALERT_1_G  Calculation of _exptl_absorpt_correction_mu
                not performed for this radiation type.
PLAT002_ALERT_2_G Number of Distance or Angle Restraints on AtSite        260 Note
PLAT003_ALERT_2_G Number of Uiso or Uij Restrained non-H Atoms ...        130 Report
PLAT042_ALERT_1_G Calc. and Reported Moiety Formula Strings Differ     Please Check
PLAT092_ALERT_4_G Check: Wavelength Given is not Cu,Ga,Mo,Ag,In Ka    0.61992 Ang.
PLAT171_ALERT_4_G The CIF-Embedded .res File Contains EADP Records          1 Report
PLAT172_ALERT_4_G The CIF-Embedded .res File Contains DFIX Records         42 Report
PLAT173_ALERT_4_G The CIF-Embedded .res File Contains DANG Records         39 Report
PLAT174_ALERT_4_G The CIF-Embedded .res File Contains FLAT Records          3 Report
PLAT175_ALERT_4_G The CIF-Embedded .res File Contains SAME Records         18 Report
PLAT176_ALERT_4_G The CIF-Embedded .res File Contains SADI Records         15 Report
PLAT177_ALERT_4_G The CIF-Embedded .res File Contains DELU Records         14 Report
PLAT178_ALERT_4_G The CIF-Embedded .res File Contains SIMU Records          7 Report
PLAT179_ALERT_4_G The CIF-Embedded .res File Contains CHIV Records          4 Report
PLAT186_ALERT_4_G The CIF-Embedded .res File Contains ISOR Records         15 Report
PLAT187_ALERT_4_G The CIF-Embedded .res File Contains RIGU Records         22 Report
PLAT301_ALERT_3_G Main Residue  Disorder .............(Resd  1  )        27% Note
PLAT301_ALERT_3_G Main Residue  Disorder .............(Resd  2  )        14% Note
PLAT302_ALERT_4_G Anion/Solvent/Minor-Residue Disorder (Resd  3  )       100% Note
```

```
PLAT302_ALERT_4_G Anion/Solvent/Minor-Residue Disorder (Resd  4  )       100% Note
PLAT302_ALERT_4_G Anion/Solvent/Minor-Residue Disorder (Resd  5  )       100% Note
PLAT302_ALERT_4_G Anion/Solvent/Minor-Residue Disorder (Resd  6  )       100% Note
PLAT302_ALERT_4_G Anion/Solvent/Minor-Residue Disorder (Resd  7  )       100% Note
PLAT302_ALERT_4_G Anion/Solvent/Minor-Residue Disorder (Resd  8  )       100% Note
PLAT302_ALERT_4_G Anion/Solvent/Minor-Residue Disorder (Resd  9  )       100% Note
PLAT302_ALERT_4_G Anion/Solvent/Minor-Residue Disorder (Resd 10  )       100% Note
PLAT302_ALERT_4_G Anion/Solvent/Minor-Residue Disorder (Resd 11  )       100% Note
PLAT302_ALERT_4_G Anion/Solvent/Minor-Residue Disorder (Resd 12  )       100% Note
PLAT302_ALERT_4_G Anion/Solvent/Minor-Residue Disorder (Resd 13  )       100% Note
PLAT302_ALERT_4_G Anion/Solvent/Minor-Residue Disorder (Resd 14  )       100% Note
PLAT302_ALERT_4_G Anion/Solvent/Minor-Residue Disorder (Resd 15  )       100% Note
PLAT302_ALERT_4_G Anion/Solvent/Minor-Residue Disorder (Resd 16  )       100% Note
PLAT302_ALERT_4_G Anion/Solvent/Minor-Residue Disorder (Resd 17  )       100% Note
PLAT304_ALERT_4_G Non-Integer Number of Atoms in ..... (Resd  1  )     234.02 Check
PLAT304_ALERT_4_G Non-Integer Number of Atoms in ..... (Resd  2  )     234.04 Check
PLAT304_ALERT_4_G Non-Integer Number of Atoms in ..... (Resd  3  )       1.52 Check
PLAT304_ALERT_4_G Non-Integer Number of Atoms in ..... (Resd  4  )       0.90 Check
PLAT304_ALERT_4_G Non-Integer Number of Atoms in ..... (Resd  5  )       2.71 Check
PLAT304_ALERT_4_G Non-Integer Number of Atoms in ..... (Resd  6  )       2.62 Check
PLAT304_ALERT_4_G Non-Integer Number of Atoms in ..... (Resd  8  )       1.85 Check
PLAT304_ALERT_4_G Non-Integer Number of Atoms in ..... (Resd  9  )       1.24 Check
PLAT304_ALERT_4_G Non-Integer Number of Atoms in ..... (Resd 10  )       1.14 Check
PLAT304_ALERT_4_G Non-Integer Number of Atoms in ..... (Resd 11  )       1.70 Check
PLAT304_ALERT_4_G Non-Integer Number of Atoms in ..... (Resd 12  )       0.95 Check
PLAT304_ALERT_4_G Non-Integer Number of Atoms in ..... (Resd 13  )       2.32 Check
PLAT304_ALERT_4_G Non-Integer Number of Atoms in ..... (Resd 14  )       3.37 Check
PLAT304_ALERT_4_G Non-Integer Number of Atoms in ..... (Resd 15  )       1.68 Check
PLAT304_ALERT_4_G Non-Integer Number of Atoms in ..... (Resd 16  )       2.11 Check
PLAT304_ALERT_4_G Non-Integer Number of Atoms in ..... (Resd 17  )       0.78 Check
PLAT333_ALERT_2_G Large Aver C6-Ring C-C Dist C5_1     -C20_1    .       1.44 Ang.
PLAT333_ALERT_2_G Large Aver C6-Ring C-C Dist C5_2     -C10_2    .       1.44 Ang.
PLAT367_ALERT_2_G Long?  C(sp?)-C(sp?) Bond  C28_1    - C77_1    .       1.52 Ang.
PLAT410_ALERT_2_G Short Intra H...H Contact  H46_1    ..H89D_1   .       1.98 Ang.
                                                      x,y,z  =      1_555 Check
PLAT410_ALERT_2_G Short Intra H...H Contact  H62_1    ..H70F_1   .       2.14 Ang.
                                                      x,y,z  =      1_555 Check
PLAT410_ALERT_2_G Short Intra H...H Contact  H66_1    ..H30E_1   .       2.09 Ang.
                                                      x,y,z  =      1_555 Check
PLAT410_ALERT_2_G Short Intra H...H Contact  H66_1    ..H90E_1   .       2.09 Ang.
                                                      x,y,z  =      1_555 Check
PLAT410_ALERT_2_G Short Intra H...H Contact  H67_1    ..H50S_1   .       1.97 Ang.
                                                      x,y,z  =      1_555 Check
PLAT410_ALERT_2_G Short Intra H...H Contact  H71_1    ..H30S_1   .       1.70 Ang.
                                                      x,y,z  =      1_555 Check
PLAT410_ALERT_2_G Short Intra H...H Contact  H77B_1   ..H78C_1   .       1.93 Ang.
                                                      x,y,z  =      1_555 Check
PLAT410_ALERT_2_G Short Intra H...H Contact  H51_2    ..H96B_2   .       2.14 Ang.
                                                      x,y,z  =      1_555 Check
PLAT410_ALERT_2_G Short Intra H...H Contact  H89A_2   ..H46A_2   .       1.69 Ang.
                                                      x,y,z  =      1_555 Check
PLAT411_ALERT_2_G Short Inter H...H Contact  H17_1    ..H49I_1   .       1.98 Ang.
                                                   -1+x,y,z  =      1_455 Check
PLAT411_ALERT_2_G Short Inter H...H Contact  H2_2     ..H85B_1   .       2.08 Ang.
                                                   x,-1+y,z  =      1_545 Check
PLAT413_ALERT_2_G Short Inter XH3 .. XHn     H1_1     ..H82E_1   .       2.00 Ang.
                                                 1-x,1-y,-z  =      2_665 Check
PLAT413_ALERT_2_G Short Inter XH3 .. XHn     H7_2     ..H51E_1   .       2.07 Ang.
                                                1-x,1-y,1-z  =      2_666 Check
PLAT413_ALERT_2_G Short Inter XH3 .. XHn     H94C_2   ..H69C_1   .       2.11 Ang.
                                                      x,y,z  =      1_555 Check
PLAT432_ALERT_2_G Short Inter X...Y Contact  S7_1     ..C2_101           3.04 Ang.
```

```
                                                     x,y,z  =      1_555 Check
PLAT432_ALERT_2_G Short Inter X...Y Contact  S7_1     ..C3_114          3.15 Ang.
                                                     x,y,z  =      1_555 Check
PLAT432_ALERT_2_G Short Inter X...Y Contact  S7_1     ..C3_113          3.30 Ang.
                                                     x,y,z  =      1_555 Check
PLAT432_ALERT_2_G Short Inter X...Y Contact  S8_1     ..C4_113          3.28 Ang.
                                                     x,y,z  =      1_555 Check
PLAT432_ALERT_2_G Short Inter X...Y Contact  S8_1     ..C4_114          3.29 Ang.
                                                     x,y,z  =      1_555 Check
PLAT432_ALERT_2_G Short Inter X...Y Contact  S9_1     ..C5_113          2.97 Ang.
                                                     x,y,z  =      1_555 Check
PLAT432_ALERT_2_G Short Inter X...Y Contact  S9_1     ..C5_114          3.25 Ang.
                                                     x,y,z  =      1_555 Check
PLAT432_ALERT_2_G Short Inter X...Y Contact  Cl1_101  ..C20_1           3.20 Ang.
                                                     x,y,z  =      1_555 Check
PLAT432_ALERT_2_G Short Inter X...Y Contact  Cl1_102  ..C10_2           2.76 Ang.
                                                     x,y,z  =      1_555 Check
PLAT432_ALERT_2_G Short Inter X...Y Contact  Cl1_102  ..C9_2            2.76 Ang.
                                               1-x,1-y,1-z  =      2_666 Check
PLAT432_ALERT_2_G Short Inter X...Y Contact  Cl1_102  ..C1_2            3.02 Ang.
                                                     x,y,z  =      1_555 Check
PLAT432_ALERT_2_G Short Inter X...Y Contact  Cl1_102  ..C8_2            3.08 Ang.
                                               1-x,1-y,1-z  =      2_666 Check
PLAT432_ALERT_2_G Short Inter X...Y Contact  Cl1_104  ..C74_1           3.17 Ang.
                                                -x,2-y,1-z  =      2_576 Check
PLAT432_ALERT_2_G Short Inter X...Y Contact  Cl1_105  ..C2_2            3.16 Ang.
                                               1-x,1-y,1-z  =      2_666 Check
PLAT432_ALERT_2_G Short Inter X...Y Contact  O1_2     ..C7_1            2.89 Ang.
                                                     x,y,z  =      1_555 Check
PLAT432_ALERT_2_G Short Inter X...Y Contact  C1_1     ..C2_114          3.18 Ang.
                                                     x,y,z  =      1_555 Check
PLAT432_ALERT_2_G Short Inter X...Y Contact  C5_1     ..C3_113          3.01 Ang.
                                                     x,y,z  =      1_555 Check
PLAT432_ALERT_2_G Short Inter X...Y Contact  C5_1     ..C3_114          3.02 Ang.
                                                     x,y,z  =      1_555 Check
PLAT432_ALERT_2_G Short Inter X...Y Contact  C5_1     ..C1_101          3.13 Ang.
                                                     x,y,z  =      1_555 Check
PLAT432_ALERT_2_G Short Inter X...Y Contact  C5_1     ..C2_113          3.17 Ang.
                                                     x,y,z  =      1_555 Check
PLAT432_ALERT_2_G Short Inter X...Y Contact  C5_1     ..C2_101          3.18 Ang.
                                                     x,y,z  =      1_555 Check
PLAT432_ALERT_2_G Short Inter X...Y Contact  C8_1     ..C3_101          2.85 Ang.
                                                     x,y,z  =      1_555 Check
PLAT432_ALERT_2_G Short Inter X...Y Contact  C8_1     ..C2_101          3.12 Ang.
                                                     x,y,z  =      1_555 Check
PLAT432_ALERT_2_G Short Inter X...Y Contact  C9_1     ..C3_113          3.01 Ang.
                                                     x,y,z  =      1_555 Check
PLAT432_ALERT_2_G Short Inter X...Y Contact  C9_1     ..C2_101          3.03 Ang.
                                                     x,y,z  =      1_555 Check
PLAT432_ALERT_2_G Short Inter X...Y Contact  C9_1     ..C4_113          3.11 Ang.
                                                     x,y,z  =      1_555 Check
PLAT432_ALERT_2_G Short Inter X...Y Contact  C9_1     ..C3_101          3.13 Ang.
                                                     x,y,z  =      1_555 Check
PLAT432_ALERT_2_G Short Inter X...Y Contact  C10_1    ..C4_113          2.98 Ang.
                                                     x,y,z  =      1_555 Check
PLAT432_ALERT_2_G Short Inter X...Y Contact  C10_1    ..C5_101          3.06 Ang.
                                                     x,y,z  =      1_555 Check
PLAT432_ALERT_2_G Short Inter X...Y Contact  C10_1    ..C6_101          3.20 Ang.
                                                     x,y,z  =      1_555 Check
PLAT432_ALERT_2_G Short Inter X...Y Contact  C11_1    ..C5_113          3.04 Ang.
                                                     x,y,z  =      1_555 Check
PLAT432_ALERT_2_G Short Inter X...Y Contact  C11_1    ..C6_101          3.13 Ang.
```

```
                                                     x,y,z  =      1_555 Check
PLAT432_ALERT_2_G Short Inter X...Y Contact  C11_1    ..C5_114          3.17 Ang.
                                                     x,y,z  =      1_555 Check
PLAT432_ALERT_2_G Short Inter X...Y Contact  C12_1    ..C5_101          3.13 Ang.
                                                     x,y,z  =      1_555 Check
PLAT432_ALERT_2_G Short Inter X...Y Contact  C12_1    ..C4_101          3.15 Ang.
                                                     x,y,z  =      1_555 Check
PLAT432_ALERT_2_G Short Inter X...Y Contact  C19_1    ..C6_113          3.19 Ang.
                                                     x,y,z  =      1_555 Check
PLAT432_ALERT_2_G Short Inter X...Y Contact  C20_1    ..C2_113          3.10 Ang.
                                                     x,y,z  =      1_555 Check
PLAT432_ALERT_2_G Short Inter X...Y Contact  C20_1    ..C3_114          3.14 Ang.
                                                     x,y,z  =      1_555 Check
PLAT432_ALERT_2_G Short Inter X...Y Contact  C20_1    ..C2_114          3.20 Ang.
                                                     x,y,z  =      1_555 Check
PLAT432_ALERT_2_G Short Inter X...Y Contact  C1_2     ..C5_102          3.04 Ang.
                                               1-x,1-y,1-z  =      2_666 Check
PLAT432_ALERT_2_G Short Inter X...Y Contact  C1_2     ..C4_107          3.18 Ang.
                                               1-x,1-y,1-z  =      2_666 Check
PLAT432_ALERT_2_G Short Inter X...Y Contact  C5_2     ..C6_102          2.87 Ang.
                                               1-x,1-y,1-z  =      2_666 Check
PLAT432_ALERT_2_G Short Inter X...Y Contact  C5_2     ..C2_102          3.02 Ang.
                                                     x,y,z  =      1_555 Check
PLAT432_ALERT_2_G Short Inter X...Y Contact  C5_2     ..C6_107          3.11 Ang.
                                                     x,y,z  =      1_555 Check
PLAT432_ALERT_2_G Short Inter X...Y Contact  C5_2     ..C1_102          3.13 Ang.
                                                     x,y,z  =      1_555 Check
PLAT432_ALERT_2_G Short Inter X...Y Contact  C5_2     ..C5_107          3.15 Ang.
                                               1-x,1-y,1-z  =      2_666 Check
PLAT432_ALERT_2_G Short Inter X...Y Contact  C5_2     ..C1_102          3.17 Ang.
                                               1-x,1-y,1-z  =      2_666 Check
PLAT432_ALERT_2_G Short Inter X...Y Contact  C5_2     ..C3_105          3.19 Ang.
                                                     x,y,z  =      1_555 Check
PLAT432_ALERT_2_G Short Inter X...Y Contact  C8_2     ..C3_102          3.17 Ang.
                                                     x,y,z  =      1_555 Check
PLAT432_ALERT_2_G Short Inter X...Y Contact  C9_2     ..C1_102          3.06 Ang.
                                               1-x,1-y,1-z  =      2_666 Check
PLAT432_ALERT_2_G Short Inter X...Y Contact  C9_2     ..C2_102          3.12 Ang.
                                                     x,y,z  =      1_555 Check
PLAT432_ALERT_2_G Short Inter X...Y Contact  C9_2     ..C6_107          3.20 Ang.
                                                     x,y,z  =      1_555 Check
PLAT432_ALERT_2_G Short Inter X...Y Contact  C10_2    ..C6_102          2.97 Ang.
                                               1-x,1-y,1-z  =      2_666 Check
PLAT432_ALERT_2_G Short Inter X...Y Contact  C10_2    ..C4_107          3.01 Ang.
                                               1-x,1-y,1-z  =      2_666 Check
PLAT432_ALERT_2_G Short Inter X...Y Contact  C10_2    ..C1_102          3.06 Ang.
                                                     x,y,z  =      1_555 Check
PLAT432_ALERT_2_G Short Inter X...Y Contact  C10_2    ..C5_102          3.08 Ang.
                                               1-x,1-y,1-z  =      2_666 Check
PLAT432_ALERT_2_G Short Inter X...Y Contact  C10_2    ..C5_107          3.12 Ang.
                                               1-x,1-y,1-z  =      2_666 Check
PLAT432_ALERT_2_G Short Inter X...Y Contact  C10_2    ..C1_105          3.19 Ang.
                                               1-x,1-y,1-z  =      2_666 Check
PLAT720_ALERT_4_G Number of Unusual/Non-Standard Labels ..........       853 Note
PLAT722_ALERT_1_G Angle   Calc      108.00, Rep      106.90 Dev...       1.10 Degree
              C903_1  -C902_1  -H90G_1     1_555  1_555  1_555     #  808 Check
PLAT722_ALERT_1_G Angle   Calc      111.00, Rep      109.70 Dev...       1.30 Degree
              C310_1  -C311_1  -H31C_1     1_555  1_555  1_555     #  899 Check
PLAT722_ALERT_1_G Angle   Calc      111.00, Rep      109.50 Dev...       1.50 Degree
              C511_1  -C512_1  -H51F_1     1_555  1_555  1_555     #  940 Check
PLAT722_ALERT_1_G Angle   Calc      108.00, Rep      109.50 Dev...       1.50 Degree
              H51E_1  -C512_1  -H51G_1     1_555  1_555  1_555     #  943 Check
```

```
PLAT722_ALERT_1_G Angle   Calc      111.00, Rep      109.50 Dev...       1.50 Degree
              H71E_1  -C712_1  -H71G_1     1_555  1_555  1_555     #  979 Check
PLAT789_ALERT_4_G Atoms with Negative _atom_site_disorder_group  #         79 Check
PLAT790_ALERT_4_G Centre of Gravity not Within Unit Cell: Resd.  #          8 Note
              C6 H5 Cl
PLAT811_ALERT_5_G No ADDSYM Analysis: Too Many Excluded Atoms ....          ! Info
PLAT860_ALERT_3_G Number of Least-Squares Restraints ............       4155 Note
```

```
   0 ALERT level A = Most likely a serious problem - resolve or explain
   0 ALERT level B = A potentially serious problem, consider carefully
  23 ALERT level C = Check. Ensure it is not caused by an omission or oversight
 135 ALERT level G = General information/check it is not something unexpected

  11 ALERT type 1 CIF construction/syntax error, inconsistent or missing data
  93 ALERT type 2 Indicator that the structure model may be wrong or deficient
   6 ALERT type 3 Indicator that the structure quality may be low
  47 ALERT type 4 Improvement, methodology, query or suggestion
   1 ALERT type 5 Informative message, check
```

It is advisable to attempt to resolve as many as possible of the alerts in all categories. Often the minor alerts point to easily fixed oversights, errors and omissions in your CIF or refinement strategy, so attention to these fine details can be worthwhile. In order to resolve some of the more serious problems it may be necessary to carry out additional measurements or structure refinements. However, the purpose of your study may justify the reported deviations and the more serious of these should normally be commented upon in the discussion or experimental section of a paper or in the "special_details" fields of the CIF. checkCIF was carefully designed to identify outliers and unusual parameters, but every test has its limitations and alerts that are not important in a particular case may appear. Conversely, the absence of alerts does not guarantee there are no aspects of the results needing attention. It is up to the individual to critically assess their own results and, if necessary, seek expert advice.

Publication of your CIF in IUCr journals

A basic structural check has been run on your CIF. These basic checks will be run on all CIFs submitted for publication in IUCr journals (*Acta Crystallographica*, *Journal of Applied Crystallography*, *Journal of Synchrotron Radiation*); however, if you intend to submit to *Acta Crystallographica Section C* or *E* or *IUCrData*, you should make sure that full publication checks are run on the final version of your CIF prior to submission.

Publication of your CIF in other journals

Please refer to the *Notes for Authors* of the relevant journal for any special instructions relating to CIF submission.

PLATON version of 03/06/2021; check.def file version of 02/06/2021

Datablock KB138m12_a - ellipsoid plot

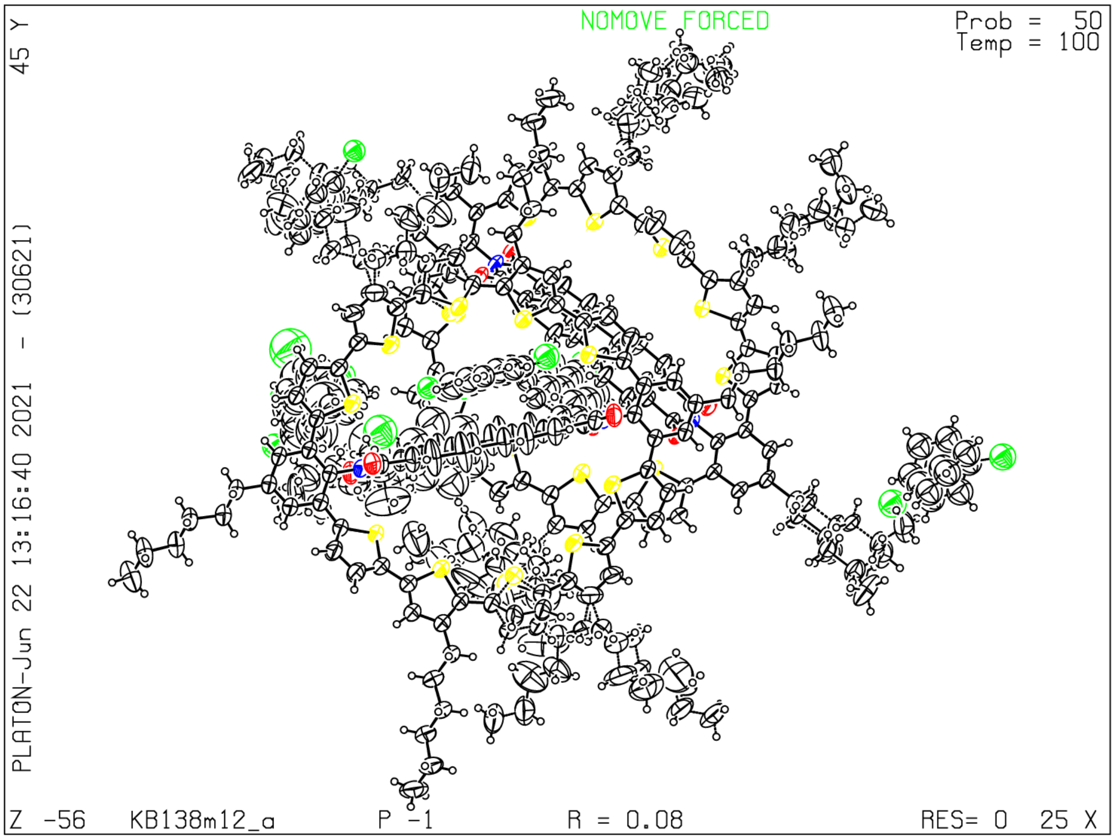

Supplement: Supplementary file 1 — Supporting Information [file ANIE-61-0-s002.zip › CCDC2102595_check-cif.pdf]
