# Supplementary material for: Macrocyclic Donor–Acceptor Dyads Composed of a Perylene Bisimide Dye Surrounded by Oligothiophene Bridges
Source: Angew Chem Int Ed Engl. 2021 Nov 23;61(1):e202113598. doi: 10.1002/anie.202113598 (PMC9299635; doi:10.1002/anie.202113598)
Supplement: Supplementary file 2 — Supporting Information [file ANIE-61-0-s001.pdf]

Angewandte Chemie
Eine Zeitschrift der Gesellschaft Deutscher Chemiker

Supporting Information

Macrocyclic Donor–Acceptor Dyads Composed of a Perylene Bisimide Dye Surrounded by Oligothiophene Bridges

*Kevin Bold, Matthias Stolte, Kazutaka Shoyama, Marco Holzapfel, Alexander Schmiedel, Christoph Lambert, and Frank Würthner**

anie_202113598_sm_miscellaneous_information.pdf
anie_202113598_sm_cif.zip

Table of Contents

Experimental Section S2

Synthetic Procedure S5

^{1}H NMR Spectra Comparison S15

Single Crystal X-ray Analysis S16

DFT Calculations S18

Electrochemistry S21

Molecular Orbital DFT Calculations S22

Spectroscopy in CH_2Cl_2 .... S22

Spectroscopy in Cyclohexane S23

Transient Absorption S23

NMR Spectra S25

Mass Spectra S35

Cartesian Coordinates Received from DFT Calculations S40

Supporting References S53

Experimental Section

General Methods

All reactions were performed in standard glass equipment. All used chemicals were purchased from commercial suppliers (*abcr/carbolution chemicals*, *Acros Organics*, *Alfa Asear*, *Merck*, *Sigma Aldrich*, *TCI* and *VWR*) and applied without further purification. CH_2Cl_2, THF and toluene were purified and dried with the commercial purification system PureSolv MD from *Innovative Technology*. Preparative column chromatography was performed with self-packed glass columns of several sizes filled with silica gel 60 M (particle size 0.040-0.063 mm, *Merc*k). The solvents CH_2Cl_2 and methanol were freshly distilled prior to use.
Flash column chromatography was performed on a PuriFLash XS-420 from *Interchim* using columns of the sizes 0012, 0025 and 0040. Silica gel deactivation was achieved by flushing the columns with a solvent mixture of cyclohexane/trimethylamine = 20:1 for two column volumes and subsequent purging with pure cyclohexane for five to ten column volumes prior to the actual purification method.

High-resolution MALDI-TOF mass spectra were measured with a ultrafleXtreme mass spectrometer from *Bruker Daltonics GmbH* using *trans*-2-[3-(4-*tert*-butylphenyl)-2-methyl-2-propenylidene]malononitrile (DCTB) as a matrix material. High-resolution ESI-TOF mass spectroscopy was carried out using a microTOF focus instrument from *Bruker Daltonics GmbH*. For melting point measurements an *Olympus* BX41 polarisation microscope with a temperature regulator TP84 from *Linkam Scientific* was used. The reported values are uncorrected. The purification by gel permeation chromatography was performed on a *Shimadzu* instrument (LC-20AD Prominence Pump, SPD-MA20A Prominence Diode Array Detector) with two preparative columns (*Japan Analytical Industries Co*., *Ltd)*. Ethanol stabilized $CHCl_3$ (Chromasolv®, *Sigma Aldrich*) was used as eluent.

^{1}H and ^{13}C NMR spectra were recorded on *Bruker* Avance III HD 400 or 600 MHz instruments using deuterated solvents. ^{13}C NMR spectra are broad band proton decoupled. Chemical shifts (δ) are listed in parts per million (ppm). Coupling constants (J) are stated in Hertz (Hz). The spectra are referenced internally to residual proton

solvent resonances or natural abundance carbon resonances. Multiplicities are reported as s = singlet, brs = broad singlet, d = doublet, dd = doublet of doublets, t = triplet, dt = doublet of triplets, q = quartet, quin = quintet, sex = sextet, m = multiplet with the chemical shift in the center of the signal.

UV/Vis absorption spectra were recorded for solutions in cuvettes (SUPRASIL®, Hellma® Analytics) on a Jasco V-670 or V-770 spectrometer and fluorescence spectra on a FLS980-D2D2-ST fluorescence spectrometer (*Edinburgh Instruments*) and were corrected against the photomultiplier sensitivity and the lamp intensity.

CV and DPV experiments were carried out with a *BASi* Epsilon potentiostat connected to a microcell apparatus from *rhd instruments* involving a 1.6 mL sample container, a platinum counter- and pseudo-reference electrode as well as a glassy carbon working electrode.

Single crystal X-ray diffraction data were collected at the P11 beamline at DESY. The diffraction data were collected by a single 360° scan ϕ sweep at 100 K. The diffraction data were indexed, integrated, and scaled using the XDS program package.[S1] In order to compensate low completeness due to single-axis measurement, two data sets were merged using the XPREP program from *Bruker*.[S2] The structures were solved using SHELXT, expanded with Fourier techniques and refined using the SHELX software package.[S3] Hydrogen atoms were assigned at idealized positions and were included in the calculation of structure factors. All non-hydrogen atoms in the major disorder part of main residues were refined anisotropically. In the crystal structures some of the side chains were disordered and modelled with restraints and constraints using standard SHELX commands RIGU, DELU, ISOR, SADI, SAME, DFIX, DANG, FLAT, SIMU, CHIV and EADP. The solvent molecules in the solvent accessible voids also had disorder and were restrained and/or constrained by a similar set of instructions.

The transient absorption spectrometer setup is based on a femtosecond laser "Solstice" from *Newport-Spectra Physics* with a fundamental wavelength of 800 nm which provides 100 fs long pulses with a repetition rate of 1 kHz. This laser source was used to pump a NOPA to generate the excitation pulses at 530 nm with a pulse length of around 50 fs. The FWHM-bandwidth of the excitation pulse was 8.5 nm and the pulse energy was set to 20 nJ (**(5T)$_2$-PBI**) and 15 nJ (**5T-PBI**). Wire grid polarizers

were used to set the pump pulse polarization to 54.7° in relation to the horizontal polarized white light continuum to achieve magic angle conditions. Another part of the laser beam was guided to a TOPAS-C from *Light-Conversion* to obtain a wavelength from 1260 nm (**(5T)$_2$-PBI**) and 1000 nm (**5T-PBI**) which was used to generate the probing white light continuum within a moving CaF_2 (**(5T)$_2$-PBI**) or sapphire crystal (**5T-PBI**). To achieve the probe range from 450 nm to 915 nm a dielectrically coated quartz glass short pass filter with 950 nm, thickness 3 mm, from *Edmund-Optics* were used. The sample was dissolved in spectroscopic grade dichloromethane from ACROS organics and the solution was filled in a quartz glass cuvette with an optical path length of 0.2 mm (**(5T)$_2$-PBI**) and 2 mm (**5T-PBI**). The optical density at the excitation wavelength was set to 0.055 for **(5T)$_2$-PBI** and 0.50 for **5T-PBI**. The IRF was ca. 80 fs as measured for stimulated Raman signals of the solvent. Further details on this spectrometer setup are provided in ref[S4].

Spectroelectrochemical experiments were performed on a Cary 5000 UV/Vis/NIR Spectrometer from *Agilent* in combination with a sample compartment consisting of a custom-made cylindrical PTFE cell with a sapphire window and an adjustable three in one electrode (6 mm platinum disc working electrode, 1 mm platinum counter and Ag/AgCl leak free reference electrode) in reflection mode. The optical path was adjusted to 100 µm with a micrometer screw. Potentials were applied with a reference potentiostat PAR 283 from *Princeton Applied Research.* Upon applying a new potential to the solution an equilibration time of 20 seconds between each measurement was employed.

DFT and TD-DFT calculations were performed by Gaussian 16[S5] using B3LYP/6-31G(d) level of theory.

Stannylated precursor compound **10**[S6] and **Ref-PBI**[S7] were synthesized according to literature known procedures. The synthesis of **5T** was recently reported.[S8]

Synthetic Procedure

4-Hexyl-2-(thiophen-2-yl)aniline (2)

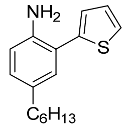

2

A solution of 2-bromo-4-hexylaniline (3.71 g, 14.5 mmol, 1.00 eq.), 2-thienylboronic acid (5.00 g, 39.1 mmol, 2.70 eq.) and $Pd(PPh_3)_2Cl_2$ (1.52 g, 2.17 mmol, 15 mol%) in degassed dioxane (50 mL) was stirred for 30 min at room temperature. Subsequently, 20 mL of aqueous K_2CO_3 (1 M) was added and the reaction mixture was refluxed overnight. The suspension was allowed to cool down to room temperature and water (20 mL) was added. The aqueous layer was extracted three times with CH_2Cl_2 (50 mL each) and the combined organic fractions were washed with brine, dried over $MgSO_4$ and the solvent was removed under reduced pressure. The crude compound was purified by column chromatography (CH_2Cl_2/*n*-hexane = 1:1) to give compound **2**.

Yield: 3.56 g, 13.7 mmol, 95%, yellow oil.

^{1}H NMR (400 MHz, CD_2Cl_2): δ/ppm = 7.35 (dd, ^3J = 5.2 Hz, ^4J = 1.2 Hz, 1H), 7.20 (dd, ^3J = 3.5 Hz, ^4J = 1.2 Hz, 1H), 7.12 (q, ^3J = 3.6 Hz, 1H), 7.08 (dd, ^4J = 2.1 Hz, ^5J = 0.4 Hz, 1H), 6.95 (dd, ^3J = 8.1 Hz, ^4J = 2.0 Hz, 1H), 6.69 (d, ^3J = 8.1 Hz, 1H), 3.92 (brs, 2H), 2.50 (t, ^3J = 7.8 Hz, 2H), 1.60-1.51 (m, 2H), 1.38 - 1.26 (m, 6H), 0.88 (t, ^3J = 6.9 Hz, 3H).

^{13}C NMR (101 MHz, $CDCl_3$): δ/ppm = 141.8, 141.5, 133.3, 130.8, 129.1, 127.6, 125.8, 125.2, 120.0, 116.1, 35.1, 31.9, 31.8, 29.1, 22.8, 14.3.

HRMS (ESI-TOF, positive mode, MeCN/CHCl3 1:1): *m*/*z* calculated for $C_{16}H_{22}NS$ $[M+H]^+$: 260.1467, found: 260.1465.

R_f: 0.63 using CH_2Cl_2/n-hexane = 1:1 as eluent.

4-Hexyl-2,6-di(thiophen-2-yl)aniline (3)

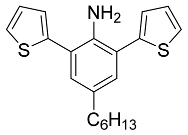

3

A solution of 4-hexylaniline (2.47 g, 7.38 mmol, 1.00 eq.), 2-thienyl boronic acid (2.83 g, 22.1 mmol, 3.00 eq.) and $Pd(PPh_3)_2Cl_2$ (777 mg, 1.11 mmol, 15 mol%) in degassed dioxane (40 mL) was stirred for 30 min at room temperature. Subsequently, 20 mL of aqueous K_2CO_3 (1 M) was added and the reaction mixture was heated to reflux for three days. The suspension was allowed to cool down to room temperature and water (20 mL) was added. The aqueous layer was extracted three times with CH_2Cl_2 (50 mL each) and the combined organic fractions were washed with brine, dried over $MgSO_4$ and the solvent was removed under reduced pressure. The crude product was purified by column chromatography (gradient of *n*-hexane/CH_2Cl_2 = 4:1 to 3:1) to give the title compound **3**.

Yield: 1.93 g, 5.67 mmol, 77%, brown oil.

^{1}H NMR (400 MHz, CD_2Cl_2): δ/ppm = 7.38 (dd, ^3J = 5.2 Hz, ^4J = 1.2 Hz, 2H), 7.24 (dd, ^3J = 3.5 Hz, ^4J = 1.2 Hz, 2H), 7.14 (dd, ^3J = 5.2 Hz, ^4J = 3.5 Hz, 2H), 7.08 (s, 2H), 4.28 (brs, 2H), 2.52 (t, ^3J = 7.6 Hz, 2H), 1.69 (quin, ^3J = 7.3 Hz, 2H), 1.40 - 1.25 (m, 6H), 0.88 (t, ^3J = 6.9 Hz, 3H).

^{13}C NMR (101 MHz, $CDCl_3$): δ/ppm = 141.3, 140.0, 132.5, 131.0, 127.7, 126.3, 125.5, 120.6, 35.0, 31.9, 31.8, 29.2, 22.8, 14.3.

HRMS (ESI-TOF, positive mode, MeCN/CHCl3 1:1): *m*/*z* calculated for $C_{20}H_{24}NS_2$ $[M+H]^+$: 342.1345, found: 342.1348.

R_f: 0.46 using CH_2Cl_2/n-hexane = 1:1 as eluent.

***N*,*N'*-Di(4-hexyl-2-(thiophen-2-yl)phenyl)-3,4:9,10-tetracarboxylic acid bisimide (4)**

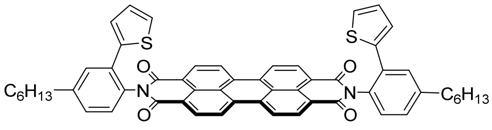

4

A suspension of perylene-3,4:9,10-tetracarboxylic dianhydride (300 mg, 765 µmol, 1.00 eq.), aniline derivate **2** (794 mg, 3.06 mmol, 4.00 eq.) and $Zn(OAc)_2$ (42.0 g, 229 µmol, 0.30 eq) in imidazole (3.0 g, 44.1 mmol) was stirred for 4 h at 120 °C under microwave irradiation. The crude solid was collected with CH_2Cl_2, adsorbed on celite and the solvent was removed under reduced pressure. The crude product-celite mixture was purified by flash column chromatography (gradient of CH_2Cl_2/*n*-hexane = 0:1 to 1:0) to give compound **4**.

Yield: 492 mg, 562 µmol, 74%, red solid.

^{1}H NMR (400 MHz, CD_2Cl_2): *δ*/ppm = 8.73 (d, ^3J = 8.0 Hz, 4H), 8.69 (d, ^3J = 8.0 Hz, 4H), 7.60 (d, ^4J = 2.0 Hz, 2H), 7.38 (dd, ^3J = 8.0 Hz, ^4J = 2.1 Hz, 2H), 7.26 (d, ^3J = 7.9 Hz, 2H), 7.15 (dd, ^3J = 3.6 Hz, ^4J = 1.2 Hz, 2H), 7.12 (dd, ^3J = 5.1 Hz, ^4J = 1.2 Hz, 2H), 6.90 (q, ^3J = 3.6 Hz, 2H), 2.77 (t, ^3J = 7.6 Hz, 4H), 1.76 (quin, ^3J = 7.5 Hz, 4H), 1.41 - 1.34 (m, 12H), 0.93 (t, ^3J = 7.0 Hz, 6H).

^{13}C NMR (101 MHz, $CDCl_3$): *δ*/ppm = 163.9, 144.6, 139.7, 135.1, 133.2, 132.1, 131.0, 130.2, 129.6, 129.3, 127.3, 126.1, 126.0, 123.5, 123.4, 35.9, 31.9, 31.3, 29.3, 22.8, 14.3.

HRMS (ESI-TOF, positive mode, MeCN/CHCl3 1:1): *m*/*z* calculated for $C_{56}H_{46}N_2NaO_4S_2$ $[M+Na]^+$: 897.2791, found: 897.2736.

M.p.: >300 °C.

R_f: 0.32 using CH_2Cl_2 as eluent.

***N*,*N'*-Tetra(4-hexyl-2-(thiophen-2-yl)phenyl)-3,4:9,10-tetracarboxylic acid bisimide (5)**

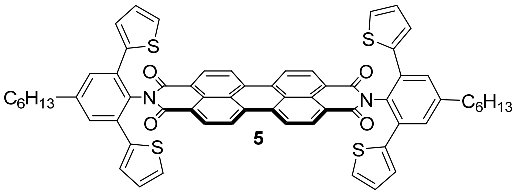

5

A suspension of perylene-3,4:9,10-tetracarboxylic dianhydride (50.0 mg, 127 μmol, 1.00 eq.), aniline derivate **3** (348 mg, 1.02 mmol, 8.00 eq.) and $Zn(OAc)_2 \cdot 2H_2O$ (42.0 mg, 229 μmol, 1.30 eq) in imidazole (600 mg, 8.81 mmol) was stirred for 14 h at 135 °C under microwave irradiation. The crude solid was collected with CH_2Cl_2, ultrasonicated, adsorbed on celite and the solvent was removed under reduced pressure. The crude product-celite mixture was purified by flash column chromatography (gradient of CH_2Cl_2/*n*-hexane = 1:1, CH_2Cl_2) to give compound **5**.

Yield: 14.6 mg, 14.1 μmol, 11%, red solid.

^{1}H NMR (400 MHz, CD_2Cl_2): *δ*/ppm = 8.57 (d, ^3J = 8.1 Hz, 4H), 8.69 (d, ^3J = 8.1 Hz, 4H), 7.55 (s, 4H), 7.13 (dd, ^3J = 3.6 Hz, ^4J = 1.1 Hz, 8H), 6.89 (dd, ^3J = 3.6 Hz, 4H), 2.80 (t, ^3J = 7.8 Hz, 4H), 1.80 (quin, ^3J = 7.1 Hz, 4H), 1.50 - 1.35 (m, 12H), 0.93 (t, ^3J = 7.0 Hz, 6H).

^{13}C NMR (101 MHz, CD_2Cl_2): *δ*/ppm = 164.0, 144.9, 139.8, 135.0, 134.6, 132.0, 131.4, 130.0, 129.0, 127.5, 127.0, 126.8, 126.5, 123.5, 123.2, 36.0, 32.1, 31.6, 29.6, 23.0, 14.3.

HRMS (MALDI-TOF, positive mode, DCTB in $CHCl_3$): *m*/*z* calculated for $C_{64}H_{50}N_2O_4S_4$ $[M]^+$: 1038.2653, found: 1038.2648.

M.p.: >300 °C.

R_f: 0.40 using CH_2Cl_2 as eluent.

***N,N'*-Di(4-hexyl-2-(5-(tributylstannyl)thiophen-2-yl)phenyl)-3,4:9,10-tetracarboxylic acid bisimide (6)**

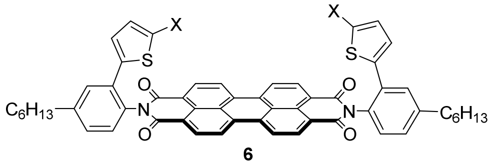

6

X = $Sn(C_4H_9)_3$

To a solution of perylene bisimide **4** (480 mg, 549 µmol, 1.00 eq.) in dry THF (100 mL) *n*-butyllithium (5.14 mL, 1.6 M in *n*-hexane, 15.0 eq.) was added dropwise under stirring at room temperature and the solution was further stirred for 2 h. Subsequently, $Sn(C_4H_9)_3Cl$ (2.53 mL, 9.32 mmol, 17.0 eq.) was added dropwise at room temperature and the solution was further stirred overnight. The reaction was quenched with water (50 mL), extracted three times with CH_2Cl_2 (50 mL each), and the combined organic layers were washed with brine, dried over $MgSO_4$ and the solvent was removed under reduced pressure. The crude residue was purified *via* flash column chromatography (deactivated silica gel, gradient of CH_2Cl_2/*n*-hexane = 0:1 to 1:0) to give the desired compound **6**.

Yield: 355 mg, 244 µmol, 45%, deep red solid.

^{1}H NMR (400 MHz, CD_2Cl_2): *δ*/ppm = 8.73 (d, ^3J = 8.0 Hz, 4H), 8.69 (d, ^3J = 8.0 Hz, 4H), 7.52 (d, ^4J = 2.0 Hz, 2H), 7.38 (dd, ^3J = 8.0 Hz, ^4J = 2.1 Hz, 2H), 7.32 (d, ^3J = 3.5 Hz, 2H), 7.27 (d, ^3J = 8.0 Hz, 2H), 6.98 (d, ^3J = 3.5 Hz, 2H), 2.77 (t, ^3J = 7.8 Hz, 4H), 1.77 (quin, ^3J = 7.8 Hz, 4H), 1.42 - 1.35 (m, 12H), 1.27 - 1.21 (m, 12H), 1.04 (sex, ^3J = 7.4 Hz, 12H), 0.93 (t, ^3J = 7.0 Hz, 6H), 0.80 (t, ^3J = 8.1 Hz, 12H), 0.64 (t, ^3J = 7.4 Hz, 18H).

^{13}C NMR (101 MHz, $CDCl_3$): *δ*/ppm = 164.0, 145.3, 144.9, 138.4, 135.8, 135.1, 133.5, 131.8, 130.3 (2 signals), 130.0, 128.9, 127.4, 126.8, 123.8, 123.7, 36.1, 32.2, 31.7, 29.6, 29.0, 27.4, 23.1, 14.3, 13.6, 10.9.

HRMS (ESI-TOF, positive mode, MeCN/CHCl3 1:1): *m*/*z* calculated $C_{80}H_{98}N_2NaO_4S_2Sn_2$ $[M+Na]^+$: 1477.4904, found: 1477.4821.

M.p.: 116-118 °C.

R_f: 0.55 using CH_2Cl_2 as eluent.

N,N'-Tetra(4-hexyl-2-(5-(tributylstannyl)thiophen-2-yl)phenyl)-3,4:9,10-tetracarboxylic acid bisimide (7)

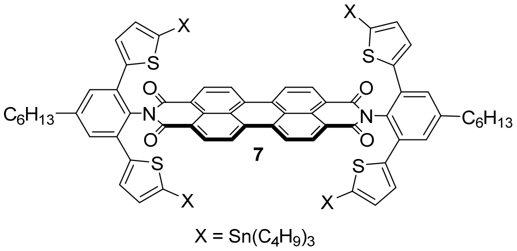

To a solution of perylene bisimide **5** (108 mg, 104 µmol, 1.00 eq.) in dry THF (22 mL) *n*-butyllithium (1.30 mL, 1.6 M in *n*-hexane, 20.0 eq.) was added dropwise under stirring at room temperature and the solution was further stirred for 1 h. Subsequently, $Sn(C_4H_9)_3Cl$ (676 µL, 2.49 mmol, 24.0 eq.) was added dropwise at room temperature and the solution was further stirred overnight. The reaction was quenched with water (15 mL), extracted three times with CH_2Cl_2 (50 mL each), and the combined organic layers were washed with brine, dried over $MgSO_4$ and the solvent was removed under reduced pressure. The crude residue was purified *via* flash column chromatography (deactivated silica gel, gradient of *n*-hexane/ CH_2Cl_2 = 1:0 to 1:1) to yield the desired compound **7**.

Yield: 45.1 mg, 20.5 µmol, 20%, deep red solid.

^{1}H NMR (400 MHz, CD_2Cl_2): δ/ppm = 8.65 (d, ^3J = 7.9 Hz, 4H), 8.62 (d, ^3J = 7.9 Hz, 4H), 7.55 (s, 4H), 7.27 (d, ^3J = 3.4 Hz, 4H), 6.94 (d, ^3J = 3.4 Hz, 4H), 2.79 (t, ^3J = 7.7 Hz, 4H), 1.79 (quin, ^3J = 7.2 Hz, 4H), 1.52-1.46 (m, 4H), 1.41-1.36 (m, 8H), 1.32 - 1.24 (m, 24H), 1.06 (sex, ^3J = 7.4 Hz, 24H), 0.93 (t, ^3J = 7.0 Hz, 6H), 0.82 (t, ^3J = 8.1 Hz, 24H), 0.67 (t, ^3J = 7.2 Hz, 36H).

^{13}C NMR (150 MHz, CD_2Cl_2): δ/ppm = 164.2, 145.3, 144.8, 138.5, 135.7, 135.3, 134.8, 132.1, 130.2, 130.1, 128.1, 127.9, 127.0, 123.8, 123.6, 36.1, 32.2, 31.6, 29.7, 29.0, 27.4, 23.0, 14.3, 13.7, 11.0.

HRMS (MALDI-TOF, positive mode, DCTB in $CHCl_3$): *m*/*z* calculated $C_{112}H_{154}N_2NaO_4S_4Sn_4$ $[M+Na]^+$: 2221.6772, found: 2221.6771.

M.p.: 183-185 °C.

R$_f$: 0.73 using CH_2Cl_2/cyclohexane = 2:1 as eluent.

***N,N'*-Di(4-hexyl-2-(5-(chloro(1,5-cyclooctadiene)platinum)thiophen-2-yl)phenyl)-3,4:9,10-tetracarboxylic acid bisimide (8)**

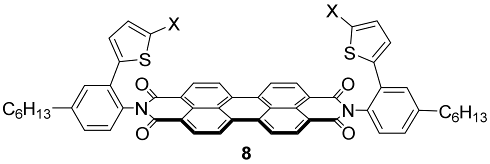

8

X = Pt(cod)Cl

A solution of **6** (50.0 mg, 34.4 µmol, 1.0 eq.) and Pt(COD)Cl_2 (28.3 mg, 75.5 µmol, 2.2 eq.) in degassed toluene (10 mL) was stirred for 2 h at 95 °C. The solvent was removed under reduced pressure and the crude product was purified *via* flash column chromatography (gradient of CH_2Cl_2/acetone = 1:0 to 20:1) to yield compound **8**.

Yield: 31.0 mg, 20.0 µmol, 58%, deep red solid.

^{1}H NMR (400 MHz, $C_2D_2Cl_4$): *δ*/ppm = 8.70 (brs, 8H), 7.59 (d, ^3J = 1.9 Hz, 2H), 7.32 (dd, ^3J = 1.9 Hz, ^3J = 8.0 Hz, 2H), 7.24 (^3J = 8.0 Hz), 7.08 (d, ^3J = 3.7 Hz, 2H), 6.77 (d, ^3J = 3.7 Hz, 2H), 5.60-5.52 (m, 4H), 4.96-4.89 (m, 4H), 2.75 (t, ^3J = 7.7 Hz, 4H), 2.47-2.12 (m, 16H), 1.74 (quin, ^3J = 7.3 Hz, 4H), 1,38-1.26 (m, 12H), 0.93 (t, ^3J = 7.0 Hz, 6H).

^{13}C NMR (101 MHz, $C_2D_2Cl_4$): *δ*/ppm = 163.6, 144.3, 141.1, 138.5, 134.7, 133.3, 131.9, 130.3, 129.7, 129.4, 129.2, 128.1, 126.3, 126.0, 123.2, 120.2, 112.9, 90.1, 35.7, 31.6, 31.5, 31.2, 30.8, 29.1, 28.3, 22.6, 14.2.

HRMS (MALDI-TOF, positive mode, DCTB in $CHCl_3$): *m*/*z* calculated for $C_{72}H_{68}Cl_2N_2O_4Pt_2S_4$ $[M]^+$:1548.3293, found: 1548.3288.

M.p.: >300 °C.

R$_f$: 0.29 using CH_2Cl_2/acetone = 20:1 as eluent.

***N*,*N'*-Tetra(4-hexyl-2-(5-(chloro(1,5-cyclooctadiene)platinum)thiophen-2-yl)phenyl)-3,4:9,10-tetracarboxylic acid bisimide (9)**

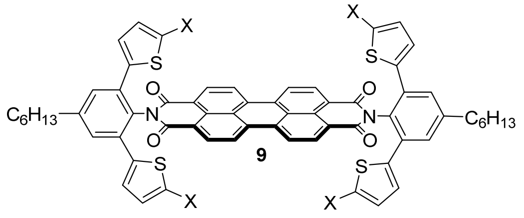

A solution of **7** (49.6 mg, 22.6 µmol, 1.0 eq.) and Pt(cod)Cl_2 (169 mg, 452 µmol, 20.0 eq.) in degassed toluene (25 mL) was stirred overnight at 80 °C. The solvent was removed under reduced pressure and the crude product was purified *via* flash column chromatography (gradient of CH_2Cl_2/MeOH = 1:0 to 99:1) to yield compound **9**.

Yield: 44.4 mg, 217 µmol, 82%, deep red solid.

^{1}H NMR (400 MHz, $C_2D_2Cl_4$): *δ*/ppm = 8.66 (brs, 8H), 7.48 (brs, 4H), 7.10 (brs, 4H), 6.77 (brs, 4H), 5.55 (brs, 8H), 4.85 (brs, 8H), 2.77-2.70 (m, 4H), 2.44-2.36 (m, 8H), 2.32-2.24 (m, 4H), 2.15-2.10 (m, 4H), 1.78-1.71 (m, 4H), 1.39-1.34 (m, 12H), 0.96-0.91 (m, 6H) .

^{13}C NMR (150 MHz, $C_2D_2Cl_4$): *δ*/ppm = 163.7, 141.2, 138.4, 134.5, 134.2, 132.1, 130.2, 126.5, 123.4, 120.2, 116.7, 116.5, 116.3, 112.9, 100.3, 99.4, 90.0, 35.7, 31.5, 30.8, 29.6, 29.2, 28.3, 22.6, 14.2.

HRMS (MALDI-TOF, positive mode, DCTB in $CHCl_3$): *m*/*z* calculated $C_{96}H_{94}Cl_4N_2O_4Pt_4S_4$ $[M]^+$: 2386.3441, found: 2386.3437.

M.p.: >300 °C.

R_f: 0.44 using CH_2Cl_2/MeOH = 20:1 as eluent.

5T-PBI

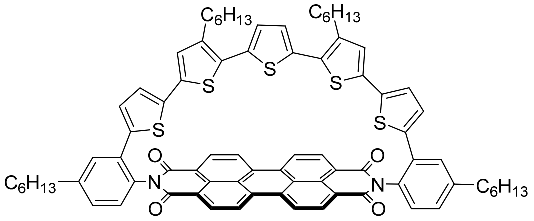

5T-PBI

To a stirred solution of **8** (31.0 mg, 20.0 µmol, 1.00 eq.) in degassed toluene (40 mL) was added dropwise the stannylated oligothiophene **10** (21.9 mg, 37.8 µmol, 1.10 eq.) in degassed toluene (1.0 mL) *via* a syringe pump over 15 h and the reaction mixture was stirred overnight at 75 °C. The solvent was removed *in vacuo* and the crude residue was washed with *n*-hexane. The crude product was redissolved in degassed CH_2Cl_2 (40 mL) and 1,1'-bis(diphenylphosphino)ferrocene (24.4 mg, 75.5 µmol, 2.20 eq.) was added. The solution was stirred for 6 h at room temperature. The solvent was removed *in vacuo* and the residue was dissolved in degassed *m*-xylene (40 mL) and stirred overnight at 120 °C. The solvent was removed under reduced pressure and the crude product was purified *via* flash column chromatography (CH_2Cl_2/cyclohexane = 1:1 to 1:0) and gel permeation chromatography ($CHCl_3$) to give the desired compound.

Yield: 7.71 mg, 5.99 µmol, 30%, red orange solid.

^{1}H NMR (600 MHz, CD_2Cl_2): δ/ppm = 8.70 (s, 8H), 7.79 (d, ^4J = 1.8 Hz, 2H), 7.45 (d, ^3J = 4.0 Hz, 2H), 7.36 (dd, ^3J = 8.0 Hz, ^4J = 1.8 Hz, 2H), 7.30 (d, ^3J = 8.0 Hz, 2H), 7.19 (d, ^3J = 4.0 Hz, 2H), 7.03 (s, 2H), 6.88 (s, 2H), 2.80 (t, ^3J = 7.7 Hz, 4H), 2.58 (t, ^3J = 7.9 Hz, 4H), 1.79 (quin, ^3J = 7.6 Hz, 4H), 1.35-1.42 (m, 8H), 1.22-1.31 (m, 20H), 0.94 (t, ^3J = 7.0 Hz, 6H), 0.83 (t, ^3J = 7.0 Hz 6H).

^{13}C NMR (150 MHz, CD_2Cl_2): δ/ppm = 164.2, 145.0, 141.5, 138.1, 137.8, 135.7, 135.5, 135.1, 132.2, 132.0, 130.7, 130.1, 129.5, 129.3, 129.2, 128.6, 127.7, 127.0, 126.9, 126.8, 124.1, 123.8, 123.6, 36.2, 32.2, 32.0, 31.7, 30.7, 29.6, 29.5, 23.1, 22.9, 14.3, 14.2.

HRMS (MALDI-TOF, positive mode, DCTB in $CHCl_3$): *m*/*z* calculated $C_{80}H_{74}N_2O_4S_5$ $[M]^+$: 1286.4252, found: 1286.4247.

UV/Vis λ_{max} (ε_{max}): CH_2Cl_2: 531 nm (64.8 × 10^3 L mol^{-1} cm^{-1}).

Fluorescence λ_{max} (λ_{ex}): Cyclohexane: 528 nm (480 nm). Φ_{fl} = <0.1%.

R$_f$: 0.32 using CH_2Cl_2 as eluent.

$(5T)_2$-PBI

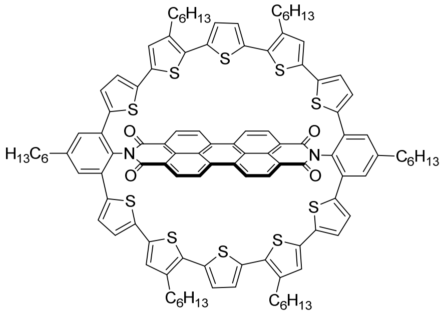

$(5T)_2$-PBI

To a stirred solution of **9** (44.4 mg, 18.5 μmol, 1.00 eq.) in degassed toluene (25 mL) was added dropwise the stannylated oligothiophene **10** (40.7 mg, 40.9 μmol, 2.20 eq.) in degassed toluene (1.0 mL) *via* a syringe pump over 15 h and the reaction mixture was stirred overnight at 75 °C. The solvent was removed *in vacuo* and the crude residue was washed with *n*-hexane. The crude product was redissolved in degassed CH_2Cl_2 (25 mL) and 1,1'-bis(diphenylphosphino)ferrocene (45.3 mg, 81.7 μmol, 4.40 eq.) was added. The solution was stirred for 6 h at room temperature. The solvent was removed *in vacuo* and the residue was dissolved in degassed *m*-xylene (25 mL) and stirred overnight at 120 °C. The solvent was removed under reduced pressure and the crude product was purified *via* flash column chromatography (cyclohexane / CH_2Cl_2 = 1:0 to 1:1) and gel permeation chromatography ($CHCl_3$) to give the desired compound.

Yield: 1.26 mg, 676 nmol, 4%, red orange solid.

^{1}H NMR (400 MHz, CD_2Cl_2): δ/ppm = 8.84 (d, ^3J = 8.4 Hz, 4H), 8.74 (d, ^3J = 7.9 Hz, 4H), 7.73 (s, 4H), 7.43 (d, ^3J = 3.9 Hz, 4H), 7.20 (d, ^3J = 3.9 Hz, 4H), 7.04 (s, 4H), 6.90 (s, 4H), 2.85 (t, ^3J = 7.8 Hz, 4H), 2.60 (t, ^3J = 7.6 Hz, 8H), 1.84 (quin, ^3J = 7.3 Hz, 4H), 1.50-1.27 (m, 44H), 0.95 (t, ^3J = 7.1 Hz, 6H), 0.88 (t, ^3J = 6.7 Hz, 12H).

^{13}C NMR (150 MHz, CD_2Cl_2): δ/ppm = 164.6, 145.1, 141.3, 138.3, 138.1, 135.9, 135.7, 134.9, 134.0, 132.3, 130.3, 129.7, 129.2, 128.9, 127.2, 127.1, 126.5, 125.8, 124.5, 123.8, 123.7, 36.2, 32.2, 32.0, 31.6, 30.7, 30.1, 29.7, 29.5, 23.1, 23.0, 14.3, 14.2.

HRMS (MALDI-TOF, positive mode, DCTB in $CHCl_3$): *m*/*z* calculated for $C_{112}H_{106}N_2O_4S_{10}$ $[M+H]^+$: 1862.5360, found: 1862.5354.

UV/Vis λ_{max} (ε_{max}): CH_2Cl_2: 380 nm (93.9 × 10^3 L mol^{-1} cm^{-1}).

Fluorescence λ_{max} (λ_{ex}): Cyclohexane: 528 nm (480 nm). Φ_{fl} = <0.1%

R_f: 0.81 using CH_2Cl_2/cyclohexane = 2:1 as eluent.

^{1}H NMR Spectra Comparison

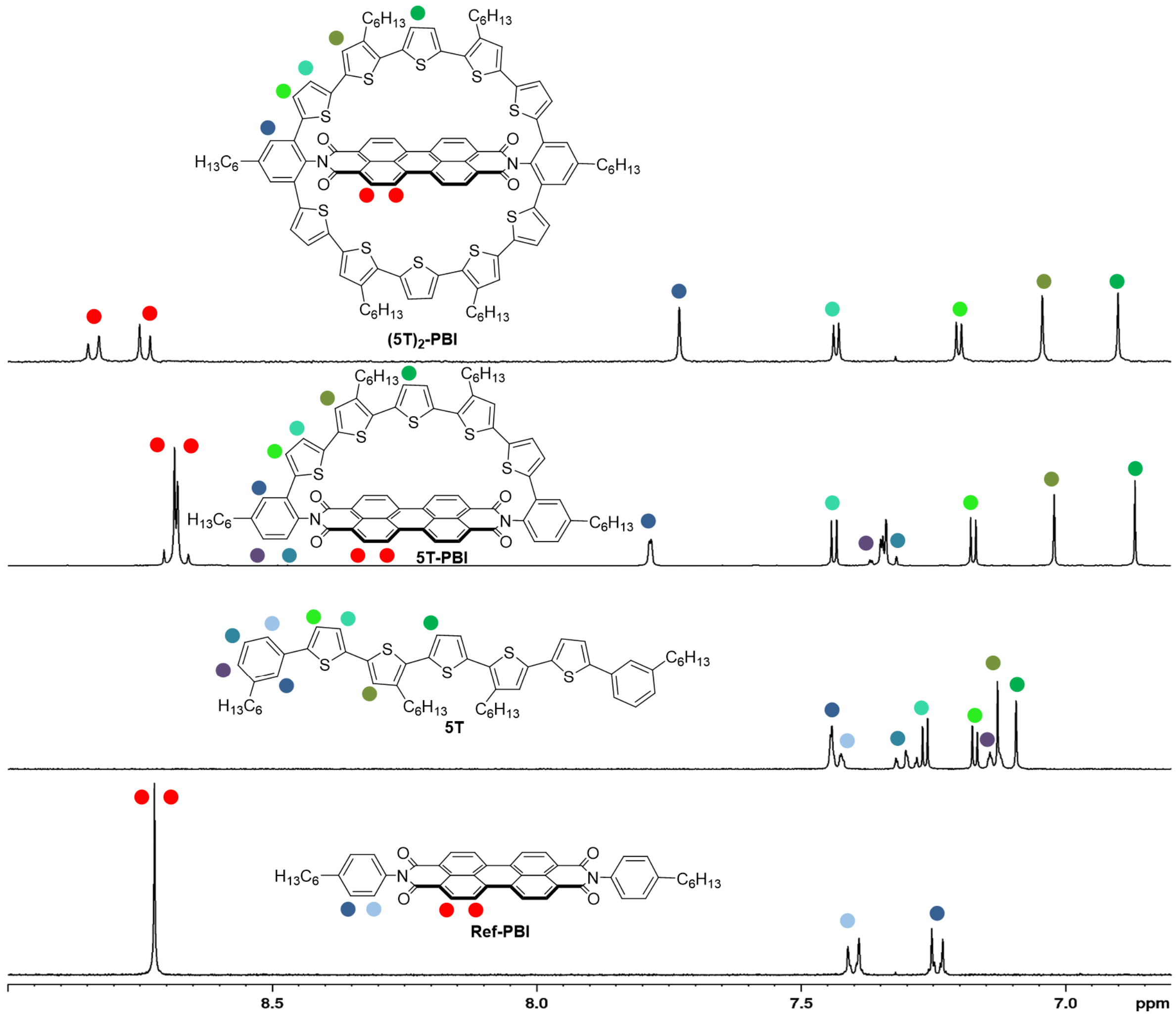

Figure S1. Aromatic region of the ^{1}H NMR spectra (400 MHz) of **Ref-PBI**, **5T**, **5T-PBI** and **(5T)₂-PBI** (from bottom to top) in CD_2Cl_2 at 298 K.

Single Crystal X-ray Analysis

Table S1. Crystal data and structure refinement for **(5T)$_2$-PBI**

CCDC Number	2102595	
Empirical formula	$C_{120.64}H_{113.21}Cl_{1.44}N_2O_4S_{10}$	
Formula weight	2026.55	
Temperature	100(2) K	
Wavelength	0.61992 Å	
Crystal system	Triclinic	
Space group	P-1	
Unit cell dimensions	a = 15.385(10) Å	α = 74.973(5)°
	b = 17.342(3) Å	β = 89.82(2)°
	c = 31.216(5) Å	γ = 85.158(16)°
Volume	8014(5) Å^3	
Z	3	
Density (calculated)	1.260 mg/m^3	
Absorption coefficient	0.203 mm^{-1}	
F(000)	3202.4	
Crystal size	0.100 x 0.100 x 0.100 mm^3	
Theta range for data collection	0.589 to 27.653°.	
Index ranges	22 ≤ h ≤ 22, 25 ≤ k ≤ 24, 45 ≤ l ≤ 46	
Reflections collected	264918	
Independent reflections	43791 [R_{int} = 0.0914]	
Completeness to theta = 21.836°	98.8%	
Absorption correction	None	
Refinement method	Full-matrix least-squares on F^2	
Data / restraints / parameters	43791 / 4155 / 2852	
Goodness-of-fit on F^2	1.109	
Final R indices [I > 2sigma(I)]	R_1 = 0.0848, wR_2 = 0.2698	
R indices (all data)	R_1 = 0.1117, wR_2 = 0.3036	
Extinction coefficient	n/a	
Largest diff. peak and hole	0.624 and −0.664 e·Å^{-3}	

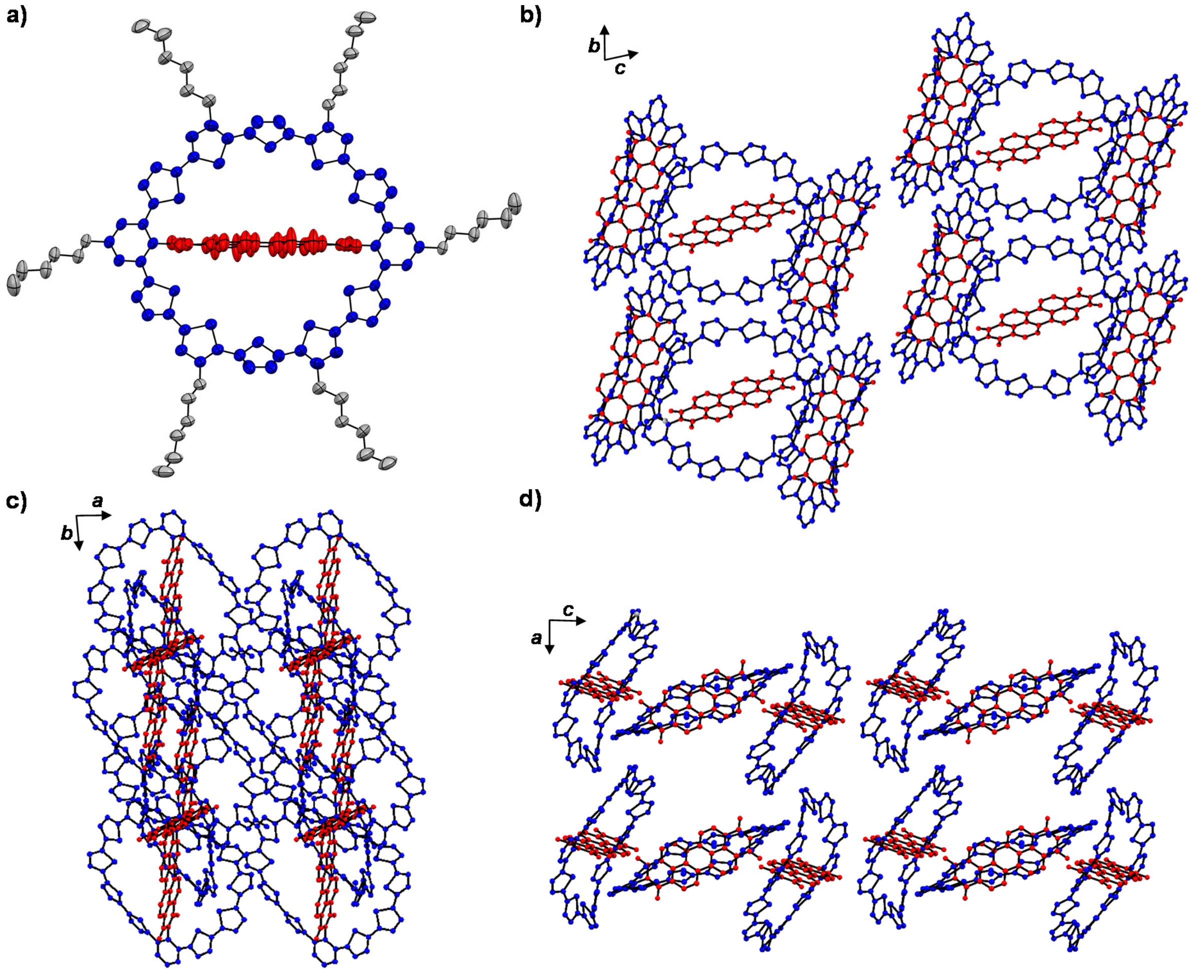

Figure S2. a) Front view of a single **(5T)$_2$-PBI** centrosymmetric molecule A (ORTEP drawing in 50% probability for thermal ellipsoids). PBI chromophore is coloured in red, macrocycle in blue and solubilizing alkyl chains in grey. Crystal packing seen approximately along the *a*-, *c*-, and *b*-axes for b), c) and d), respectively. Heavily disordered aliphatic chains as well as solvent molecules were omitted for clarity.

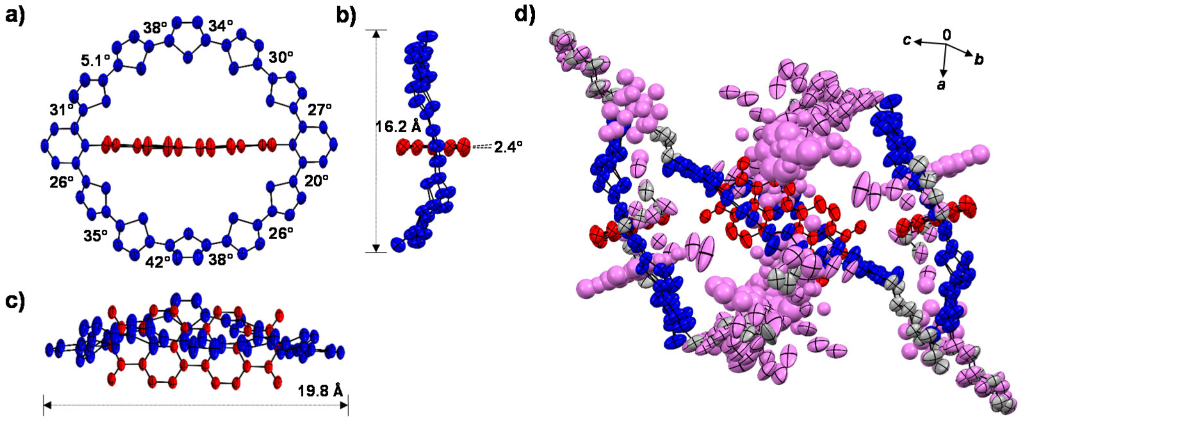

Figure S3. a) Front, b) side and c) top view onto the unsymmetric molecule B of **(5T)$_2$-PBI**. Heavily disordered aliphatic chains as well as solvent molecules were omitted for clarity. d) Unit cell including all structural disorder (violet) and aliphatic chains (grey). The ellipsoids are set to 50% probability.

DFT Calculations

Rotational Barrier:

To estimate the rotational barrier (Figure S4) of the imide substituent of **8**, calculations were conducted only on one half-segment, namely the naphthalene imide part (Figure S4b). In order to estimate the energy cost of this rotation a dihedral angle scan of α in 0.5° intervals was performed (Figure S4a). Here, the change of the total energies ΔE depending on the torsion angle α is plotted. This angle α, which was modified during the scan, is highlighted in Figure S4c. The initial α of 90° between the phenyl substituent and the naphthalene monoimide core was readily reduced until complete rotation of the substituent. In the starting geometry (Figure S4c) the sulphur atom points away from the naphthalene imide core, whereas during the rotation this subunit undergoes a conformational change at α = 59° (Figure S4d) towards the core due to the repulsive hydrogen-core interaction. Further rotation up to –26° leads to an outer plane uplifting of the nitrogen atom (Figure S4e) and an almost perpendicular angle between the thiophene and the phenyl group. This geometry also resembles the structure with the highest total energy level during the entire rotation process and therefore the closest structure to the "real" transition state (TS). This geometry was the basis for the TS calculation of which the result is shown in Figure S4f. The energy difference between this TS geometry and the fully relaxed monoimide is 114 kJ mol^{-1} and can therefore be considered as the rotational barrier or the Gibbs free energy of activation $\Delta G^{\ddagger}$. To determine the half life time of the rotation event the reaction rate k_{rot} according to Eyring has to be determined first (Eq. 1)

$$k_{\mathrm{rot}} = \frac{k_{\mathrm{B}}T}{h} \cdot e^{-\frac{\Delta G^{\ddagger}}{RT}} \quad . \qquad (1)$$

Here k_B is the Boltzmann constant, T the temperature R and h the Planck constant. For T = 298.15 K (room temperature) and 348.15 K (macrocyclization reaction temperature) the resulting k_{rot} values are $6.60 \cdot 10^{-8}$ s^{-1} and $5.70 \cdot 10^{-5}$ s^{-1}, respectively. The half life time $t_{1/2}$ (Eq. 2) can be calculated by the following equation:

$$t_{1/2} = \frac{\ln(2)}{4k_{\mathrm{rot}}}. \qquad (2)$$

The results of $t_{1/2}$ = 30 days at room temperature (25 °C) and around 51 min at 75 °C show the importance of elevated temperatures during the final macrocyclization reaction towards **5T-PBI**. [S9]

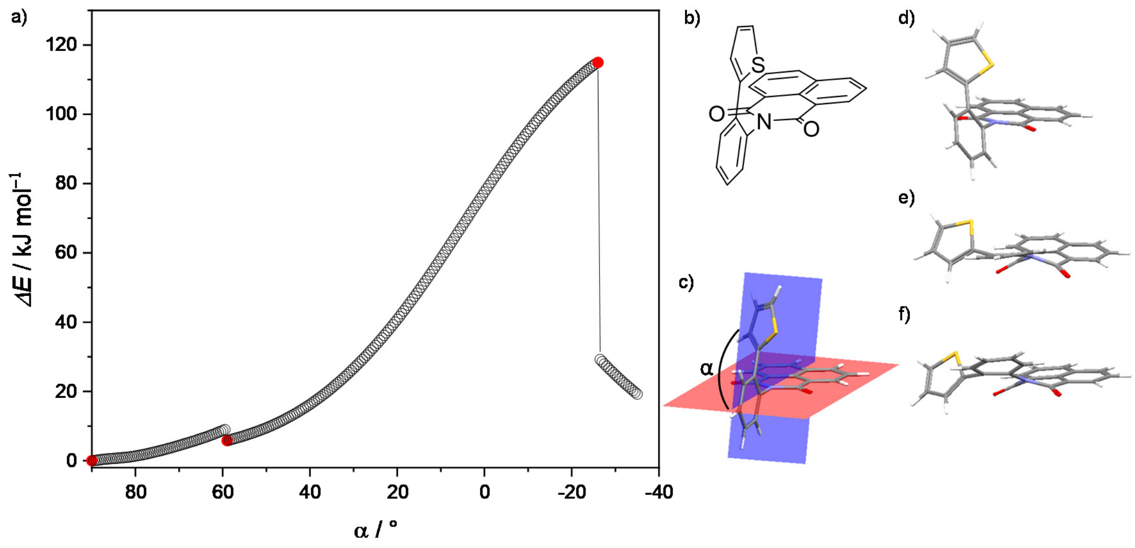

Figure S4. a) Plot of the change in total energy Δ*E* against the dihedral angle α. b) Chemical structure of the molecular fragment used for the calculations. c) Geometry optimized structure of the starting geometry for the rotational scan and the starting angle α incorporated by the planes of the naphthalene (red) and phenylene (blue) subunit. d) Geometry with α = 59 °e) Highest energy geometry with α = –26 °. f) Geometry of the TS. All calculations were conducted with DFT at the B3LYP/6-31G(d) level of theory.

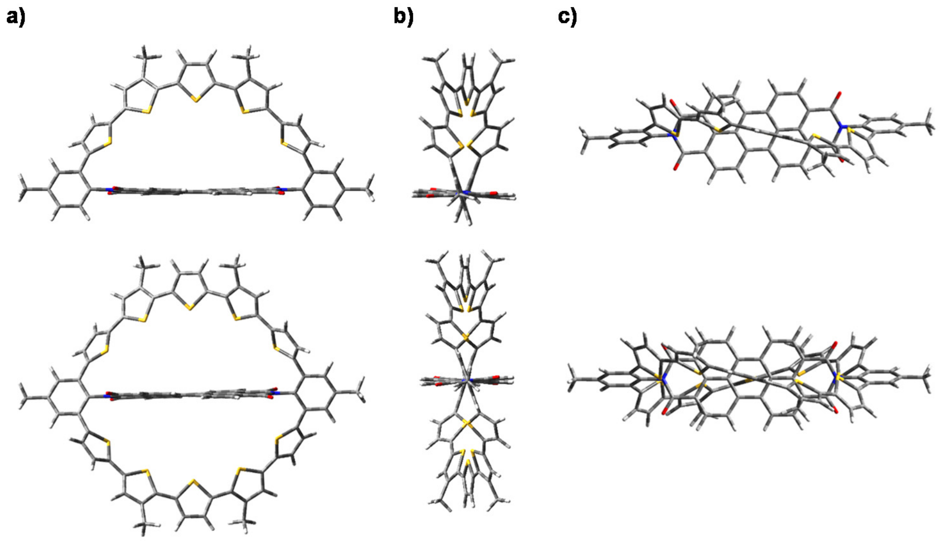

Figure S5. Side view (a), view along the *N,N´*-axis (b) and top view (c) onto the PBI π-surface of geometry optimized structures of **5T-PBI** and **(5T)$_2$-PBI** (from top to bottom). The quantum mechanics calculations were carried out on the level of B3LYP density functional with the 6-31G(d) basis set as implemented in with Gaussian 16. Aliphatic chains were replaced by methyl groups. Color code: carbon = light grey, hydrogen = white, nitrogen = blue, oxygen = red, sulfur = yellow.

Strain energies:

The strain energies of the macrocycles **(5T)$_2$-PBI** and **5T-PBI** were calculated as follows: The connecting C-C bonds between two thiophene units of the bridges were removed virtually from the optimized geometries of **(5T)$_2$-PBI** and **5T-PBI** and the obtained radicals were saturated by thiophene capping molecules to retain the local

environment of the two ends. Geometry optimization leads to the lowest energy conformation of the resulting structures and complete macrocyclic induced strain release of both subunits. Figure S6 shows the optimized geometries of these open macrocycles **11** and **12** as well as capping bithiophene **13**.

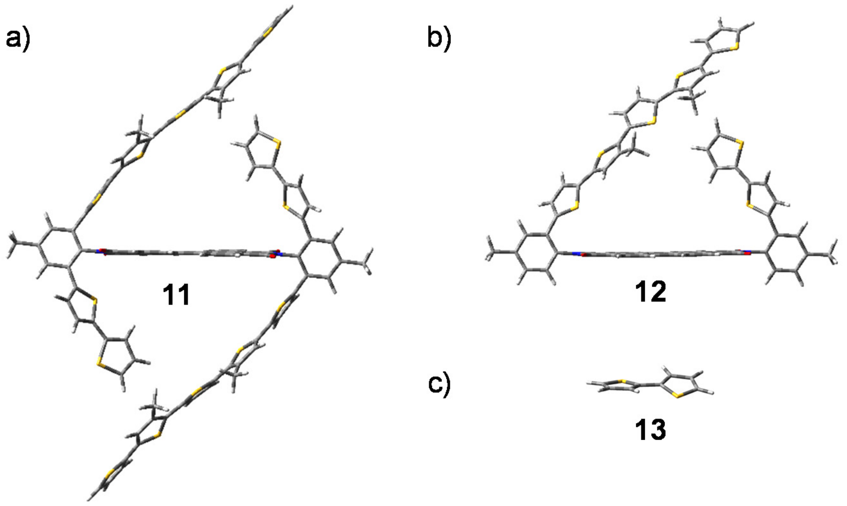

Figure S6. Front view of the optimized geometries of the non-cyclic structures **11** and **12** as well as bithiophene **13.** The quantum mechanics calculations were carried out on the level of B3LYP density functional with the 6-31G(d) basis set as implemented in with Gaussian 16. Aliphatic chains were replaced by methyl groups. Color code: carbon = light grey, hydrogen = white, nitrogen = blue, oxygen = red, sulfur = yellow.

The strain energies of the respective macrocycles (E_{Strain}) were determined by comparing the lowest energy conformation of the respective macrocycles ($E_{\mathbf{5T\text{-}PBI}}$ or $E_{\mathbf{(5T)2\text{-}PBI}}$) to the homodesmic reaction product[S10] of the linear structures **11** and **12** ($E_{\mathbf{11}}$ or $E_{\mathbf{12}}$) and the bithiophene cap **13** ($E_{\mathbf{13}}$):

$$E_{\mathrm{Strain},\ \mathbf{(5T)2\text{-}PBI}} = (E_{\mathbf{5T2\text{-}PBI}} + 2E_{\mathbf{13}}) - E_{\mathbf{11}} = 30.6\ \mathrm{kJ\ mol^{-1}} \quad (3)$$

$$E_{\mathrm{Strain},\ \mathbf{5T\text{-}PBI}} = (E_{\mathbf{5T\text{-}PBI}} + E_{\mathbf{13}}) - E_{\mathbf{12}} = 13.9\ \mathrm{kJ\ mol^{-1}} \quad (4)$$

Table S2: First excited state (S_1) energy predictions of **5T-PBI** and **(5T)$_2$-PBI** with TDDFT at the B3LYP/6-31G(d) level of theory (H = HOMO, L = LUMO).

**Compound**	**Excitation Energy** / eV	**Wavelength** / nm	**Osc. Strength**	**Contribution**
**5T-PBI**	1.18	1051	0.0000	H → L (100%)
**(5T)$_2$-PBI**	1.32	937	0.0001	H → L (100%)

Electrochemistry

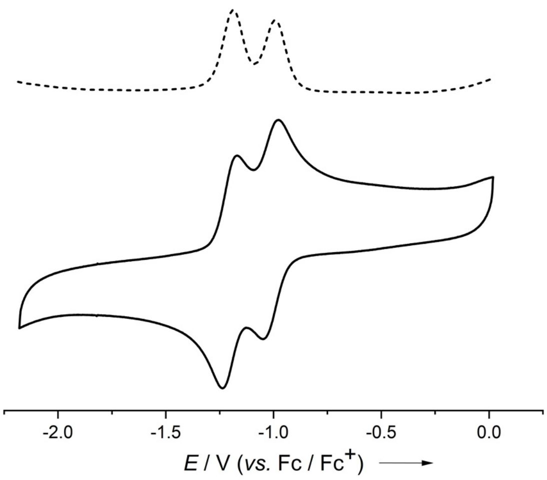

Figure S7 Cyclic voltammogram (solid line) initiated in the forward (positive-going) scan direction (marked by an arrow) at a scan rate of 100 mV s^{-1} and differential pulse voltammogram (dashed line) of **Ref-PBI** in CH_2Cl_2 with Bu_4NPF_6 at room temperature (c_0 = 10^{-4} M).

In order to demonstrate the involvement of four electrons in the entire oxidation process of **(5T)2-PBI** we decided to utilize the baseline (recorded prior to the actual measurement) corrected DPV data which was compared to those of **5T-PBI**. It is evident that for respective reduction of both macrocyclic PBI subunits two electrons are transferred. By comparing the PBI's DPV reduction to the oligothiophene's oxidation wave integrals the relative amount of transported charges can be assigned (Figure S8).

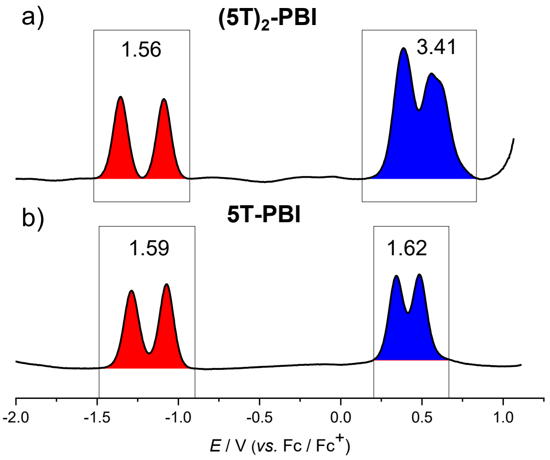

Figure S8. DPV measurements of a) **(5T)2-PBI** and b) **5T-PBI** in CH_2Cl_2 solutions with Bu_4NPF_6 at room temperature (c_0 = 10^{-4} M). The wave integrals for PBI reduction and oligothiophene oxidation are highlighted in red and blue, respectively. The straight black lines mark the integration limits and the values above the waves represent the absolute integral in arbitrary units. The graphs are baseline corrected to ease the integration.

The ratio of both signals in reduction and oxidation determined by integration for **5T-PBI** is 1.62/1.59 = 1.02 ≈ 1 and for **(5T)$_2$-PBI** 3.41/1.56 = 2.19 ≈ 2, respectively. The ratios prove that approximately double the amount of charges was transferred in the oxidation process of **(5T)$_2$-PBI** in comparison to the reduction. For **5T-PBI** an equal amount of charges are involved in reduction and oxidation.

Molecular Orbital DFT Calculations

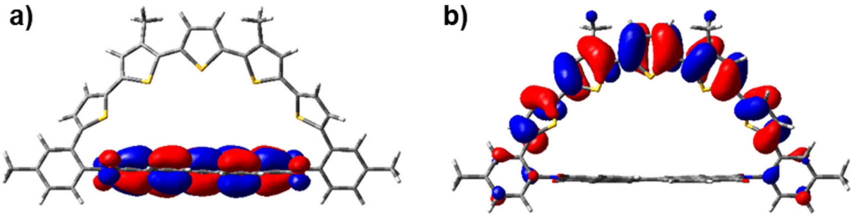

Figure S9. a) LUMO and b) HOMO of **5T-PBI** based on geometry optimized structures from DFT calculations. The quantum mechanics calculations were carried out on the level of B3LYP density functional with the 6-31G(d) basis set as implemented in with Gaussian 16.

Spectroscopy in CH_2Cl_2

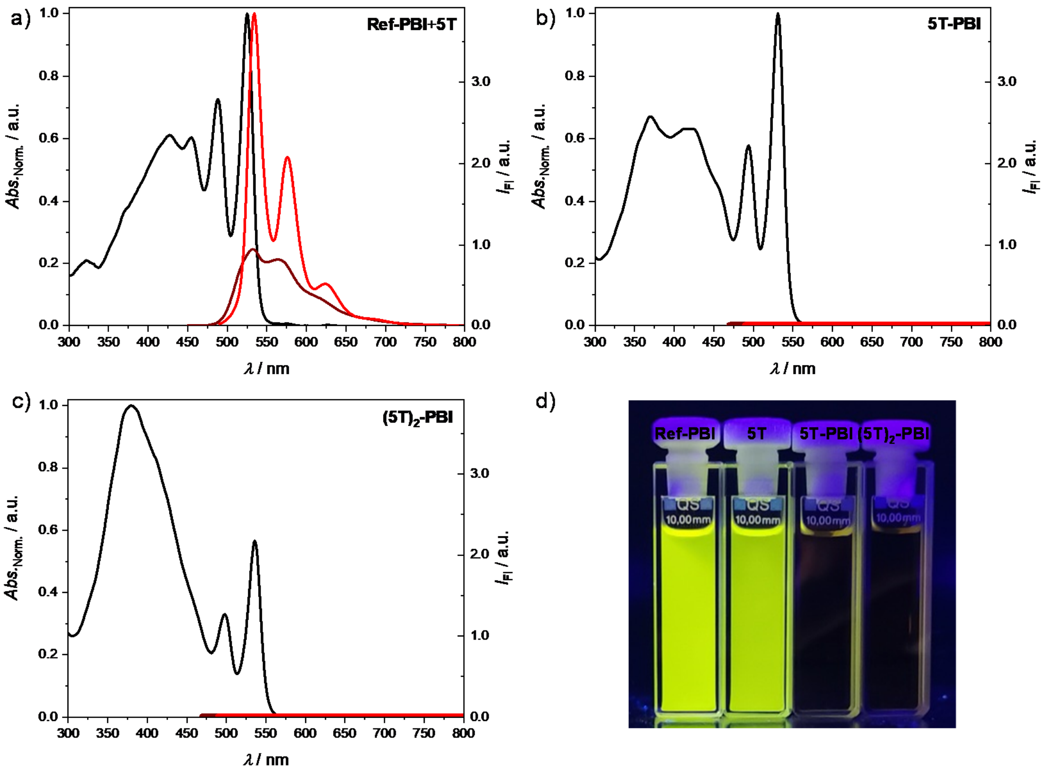

Figure S10. Normalized UV/Vis spectra (black lines) and emission spectra with the excitation wavelengths λ_{ex} = 400 nm (maroon lines) and λ_{ex} = 480 nm (red lines) of a) a 1:1 mixture of **Ref-PBI + 5T**, b) **5T-PBI** and c) **(5T)$_2$-PBI**. All UV/Vis and emission (c_0 = 10^{-7} M) measurements were carried out in CH_2Cl_2 at room temperature. d) Photograph of **Ref-PBI**, **5T**, **5T-PBI** and **(5T)$_2$-PBI** (from left to right) in CH_2Cl_2 under 365 nm UV light irradiation.

Spectroscopy in Cyclohexane

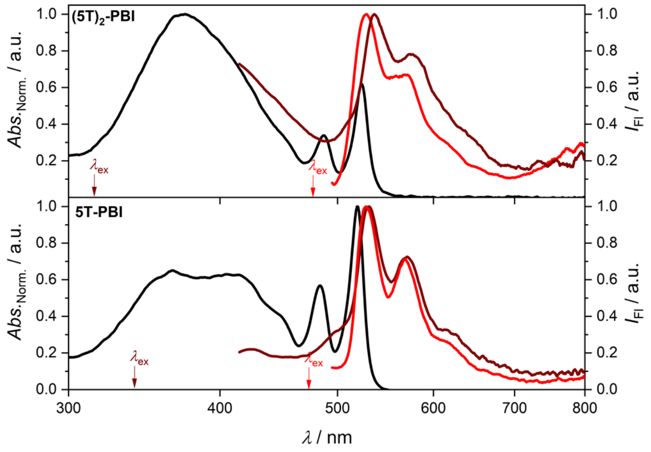

Figure S11. Normalized UV/Vis absorption (black solid) and emission (red: λ_{ex} = 480 nm, maroon: λ_{ex} = 340/310 nm) spectra of **5T-PBI** (bottom) and **(5T)$_2$-PBI** (top) in cyclohexane at room temperature (c_0 = 10^{-7} M). The wavelengths for excitation to obtain the fluorescence spectra are highlighted by arrows.

Table S3. Spectroscopic properties of **5T-PBI** and **(5T)$_2$-PBI** in cyclohexane at room temperature.

	$\lambda_{abs,max}$ [a] / nm	$\lambda_{em,max}$ [a], [b] / nm	$\lambda_{em,max}$ [a], [c] / nm	$\Delta\tilde{\nu}_{Stokes}$ (PBI) [a] / cm^{-1}	Φ_{fl} [a],[d] / %
**5T-PBI**	519	531	528	329	<< 0.1
**(5T)$_2$-PBI**	374	536	528	145	<< 0.1

[a] c_0 = 10^{-7} M. [b] λ_{ex} = 340/310 nm. [c] λ_{ex} = 480 nm [d] The fluorescence quantum yields of the PBI were measured relative to *N,N'*-bis(2,6-diisopropylphenyl)-1,6,7,12-tetraphenoxy-perylenebis(dicarboximide)[S11] (96% in $CHCl_3$) as a reference at four different excitation wavelengths in the spectral region of the PBI absorption band.

Transient Absorption

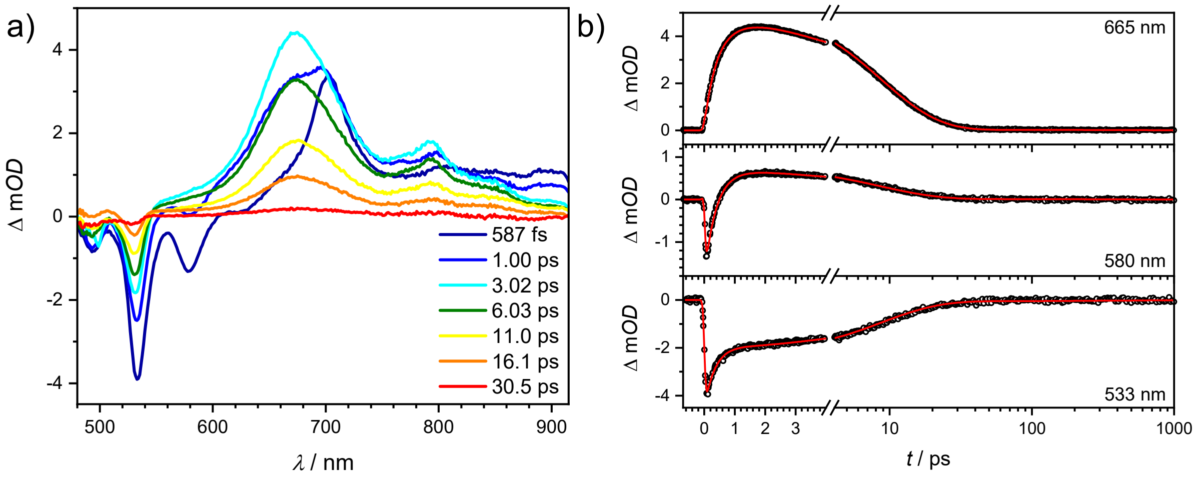

Figure S12. a) Transient absorption spectra of **5T-PBI** in CH_2Cl_2 after excitation at 530 nm and b) time scans and fit (red line) at selected wavelengths.

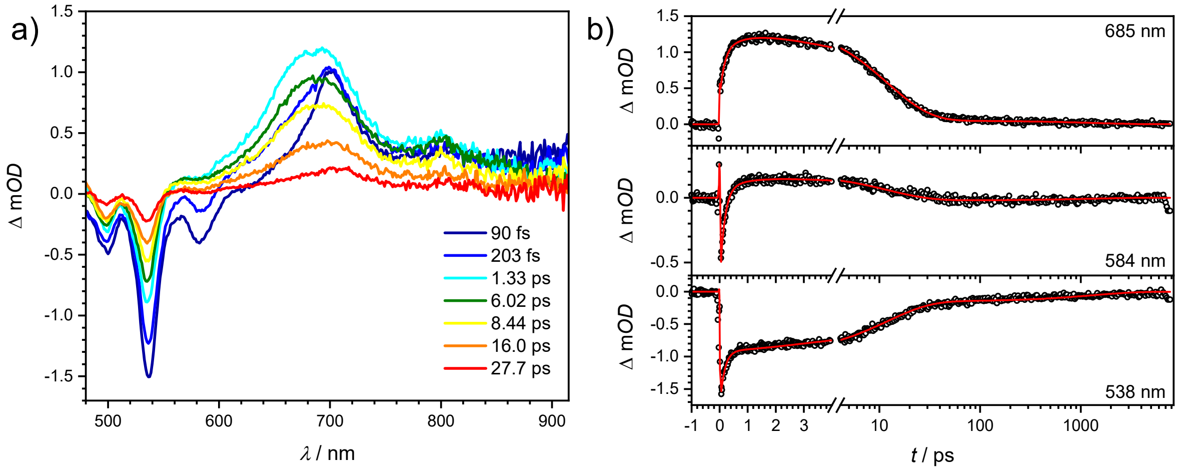

Figure S13. a) Transient absorption spectra of **(5T)$_2$-PBI** in CH_2Cl_2 after excitation at 530 nm and b) time scans and fit (red line) at selected wavelengths.

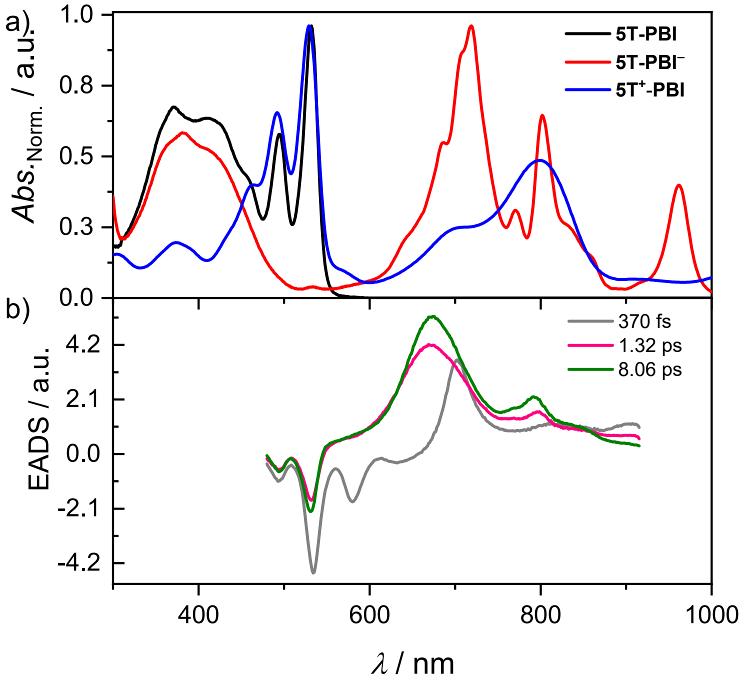

Figure S14. a) Normalized UV/Vis/NIR absorption spectra of **5T-PBI** (black line) upon electrochemical reduction to **5T-PBI**$^{\bullet-}$ (red line) and electrochemical oxidation to **5T**$^{\bullet+}$**-PBI** (blue line) in CH_2Cl_2 solutions with Bu_4NPF_6 at room temperature (c_0 = 10^{-4} M). b) Evolution associated difference spectra (EADS) and lifetimes from a global fit analysis of the transient spectra of **5T-PBI** obtained by excitation at 530 nm in CH_2Cl_2 (c_0 = 10^{-4} M) at room temperature.

NMR Spectra

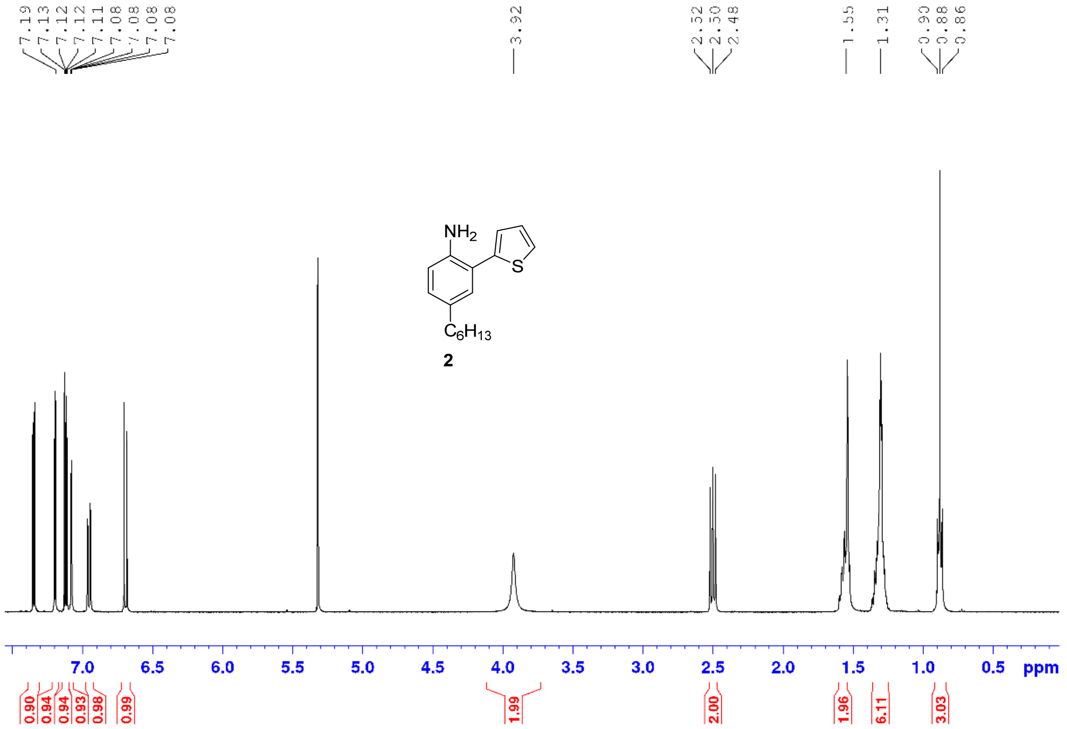

Figure S15. ^{1}H NMR spectrum of **2** in CD_2Cl_2 at 298 K.

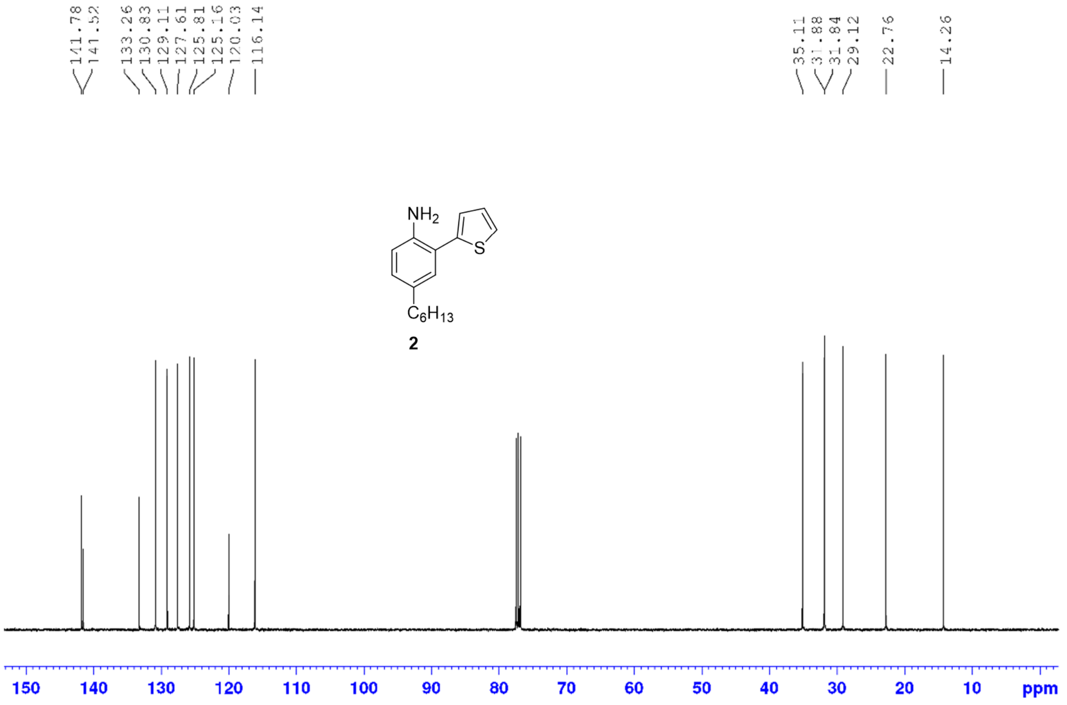

Figure S16. ^{13}C NMR spectrum of **2** in $CDCl_3$ at 298 K.

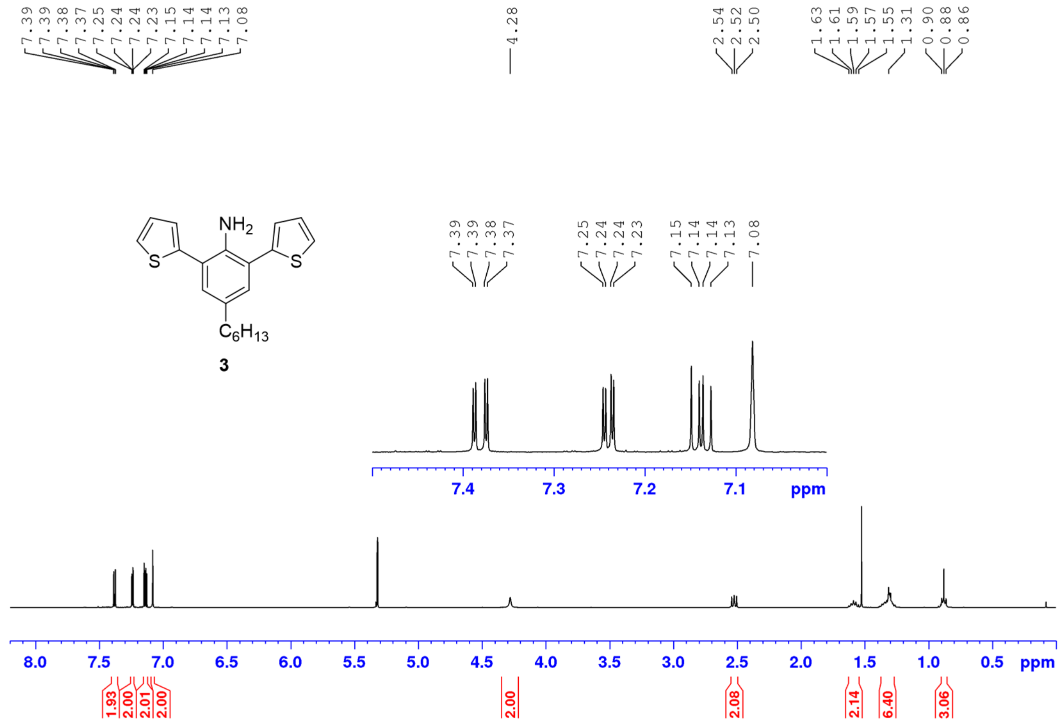

Figure S17. ^{1}H NMR spectrum of **3** in CD_2Cl_2 at 298 K.

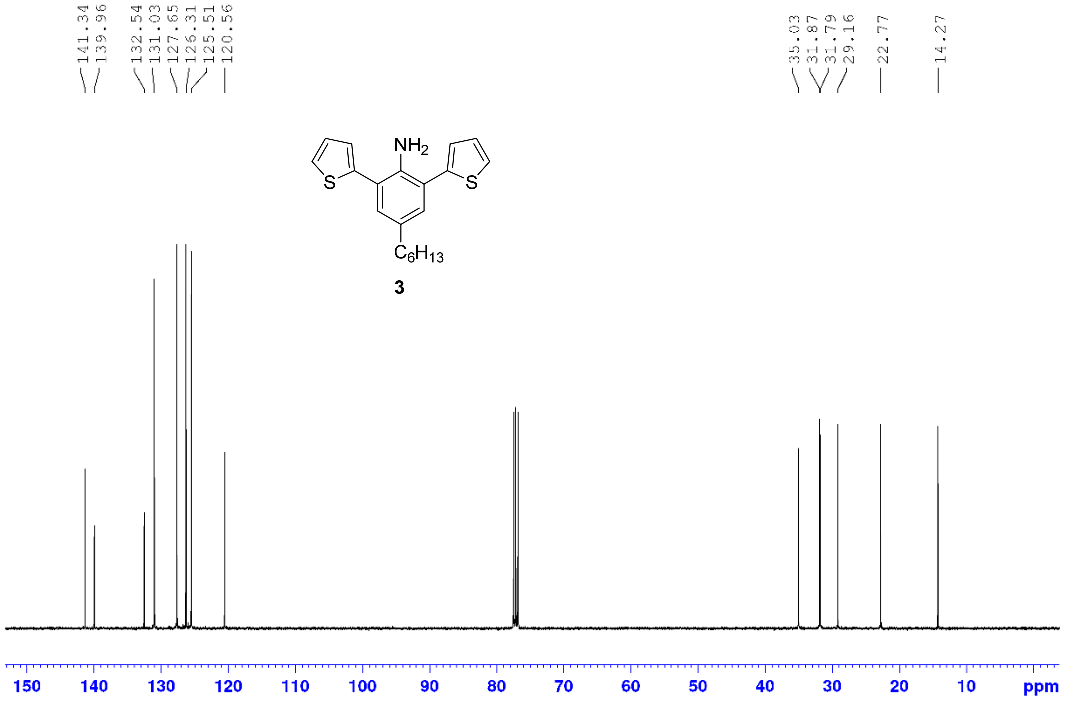

Figure S18. ^{13}C NMR spectrum of **3** in $CDCl_3$ at 298 K.

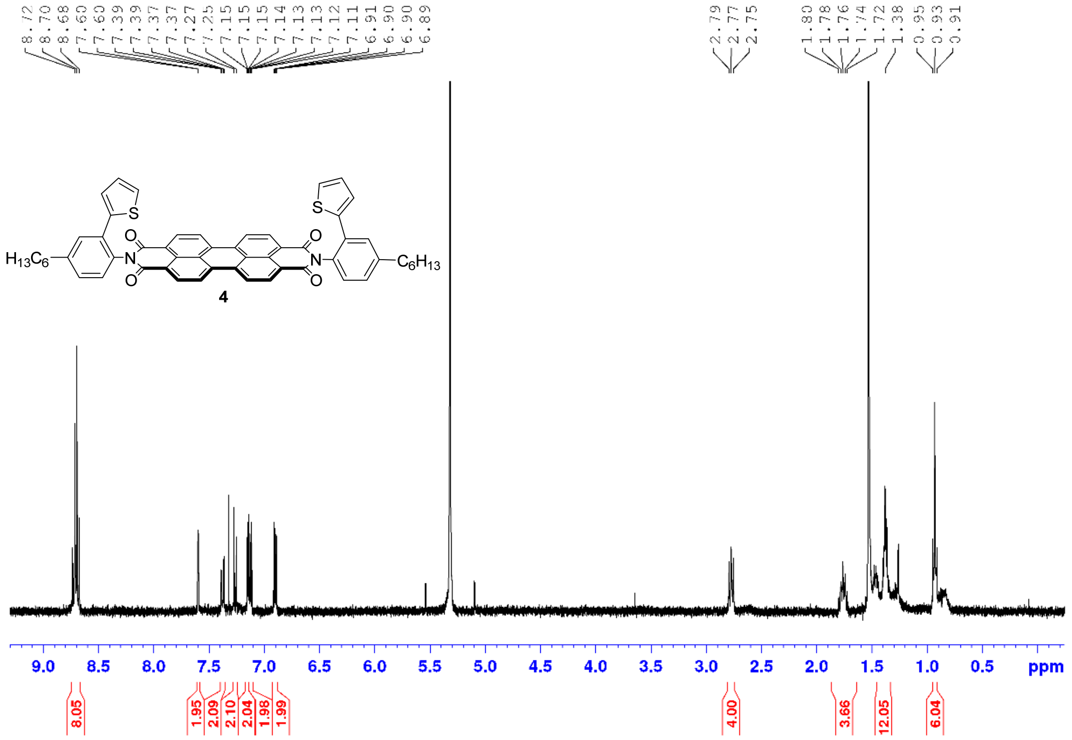

Figure S19. ^{1}H NMR spectrum of **4** in CD_2Cl_2 at 298 K.

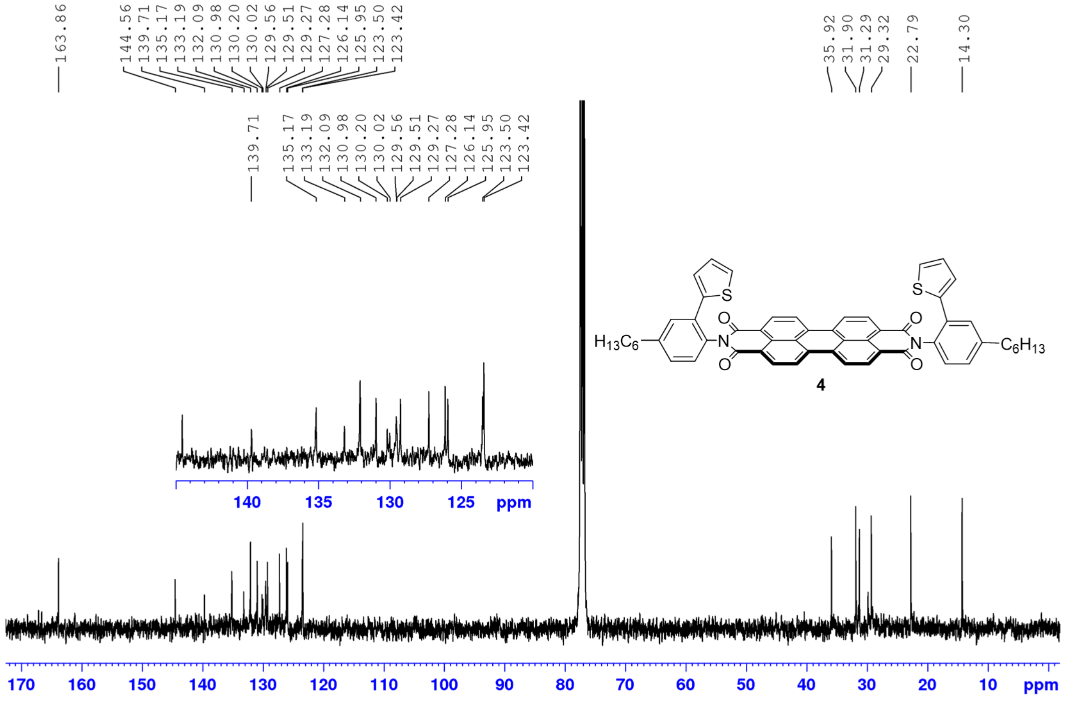

Figure S20. ^{13}C NMR spectrum of **4** in $CDCl_3$ at 298 K.

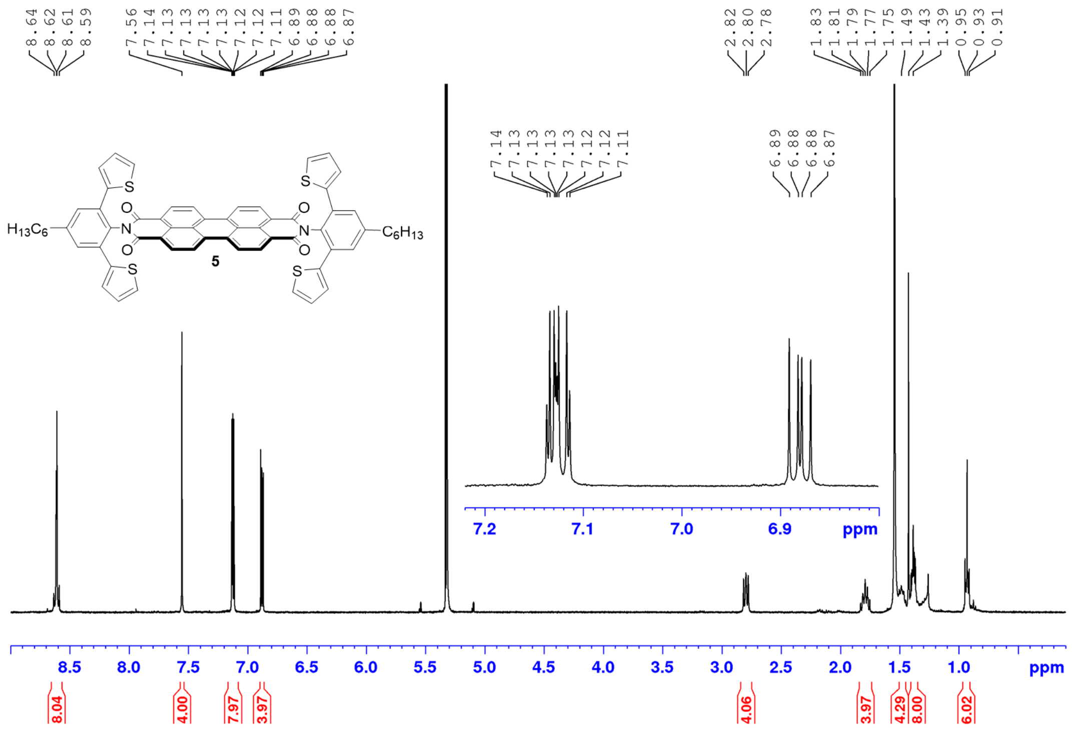

Figure S21. ^{1}H NMR spectrum of **5** in CD_2Cl_2 at 298 K.

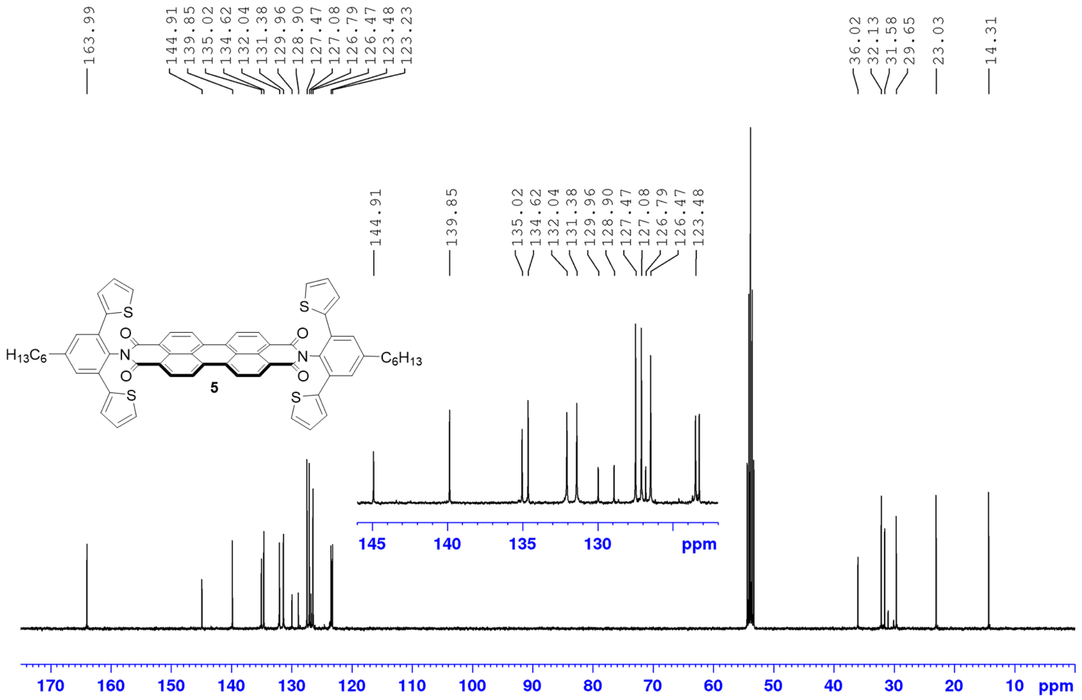

Figure S22. ^{13}C NMR spectrum of **5** in CD_2Cl_2 at 298 K.

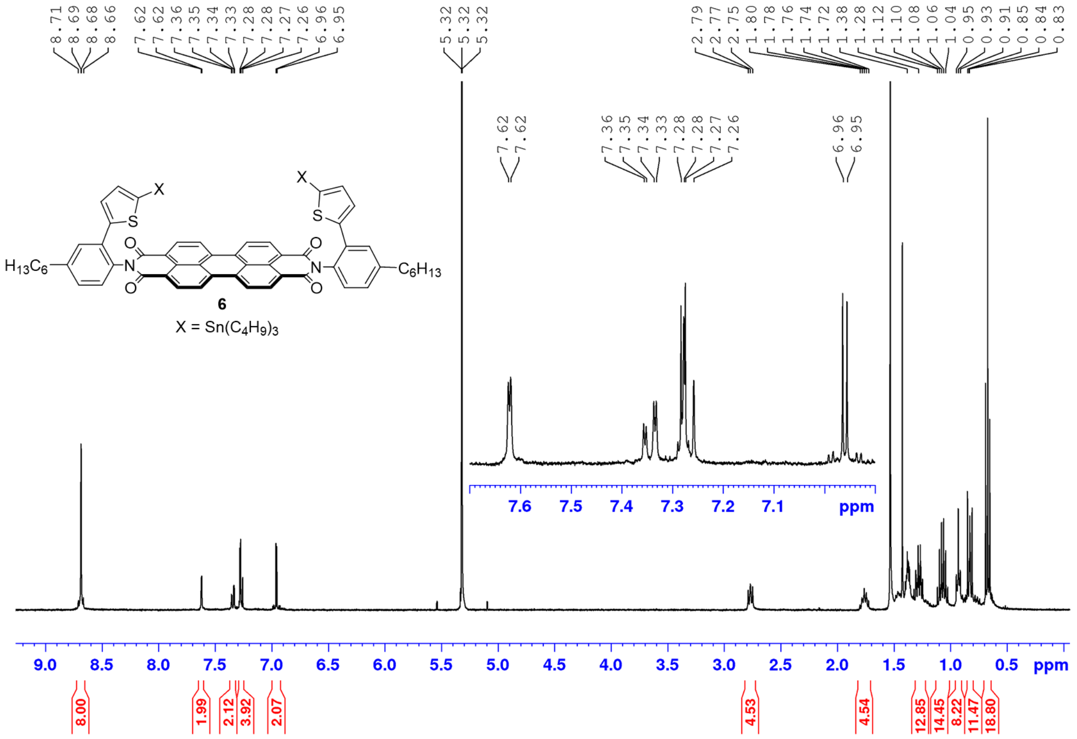

Figure S23. ^{1}H NMR spectrum of **6** in CD_2Cl_2 at 298 K.

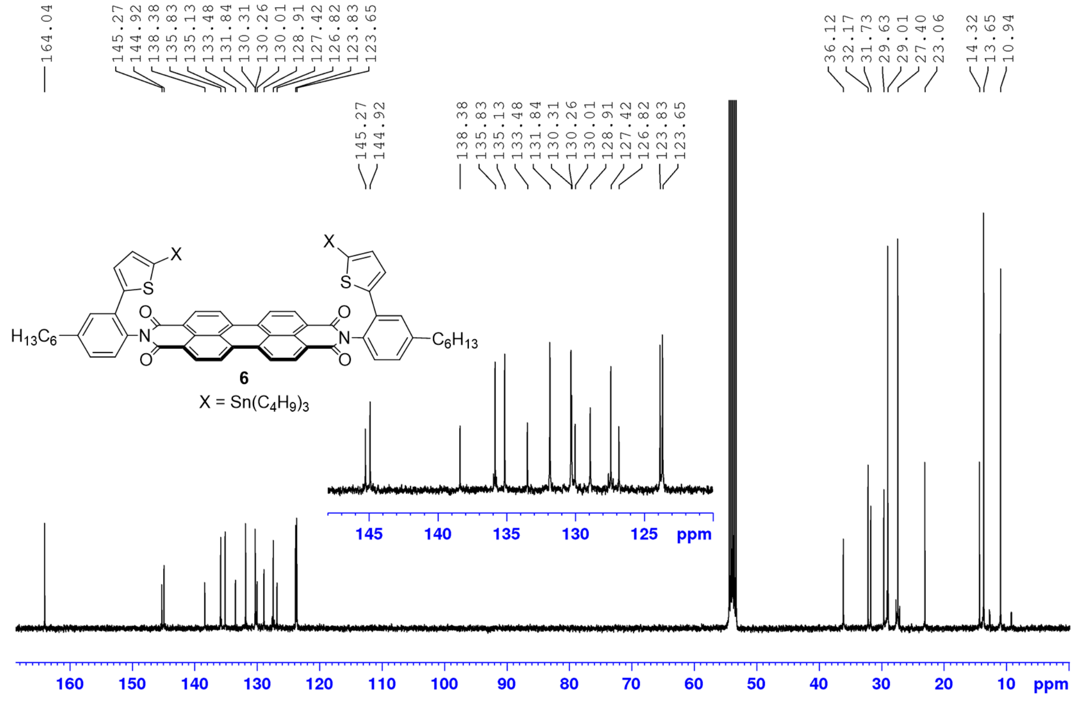

Figure S24. ^{13}C NMR spectrum of **6** in CD_2Cl_2 at 298 K.

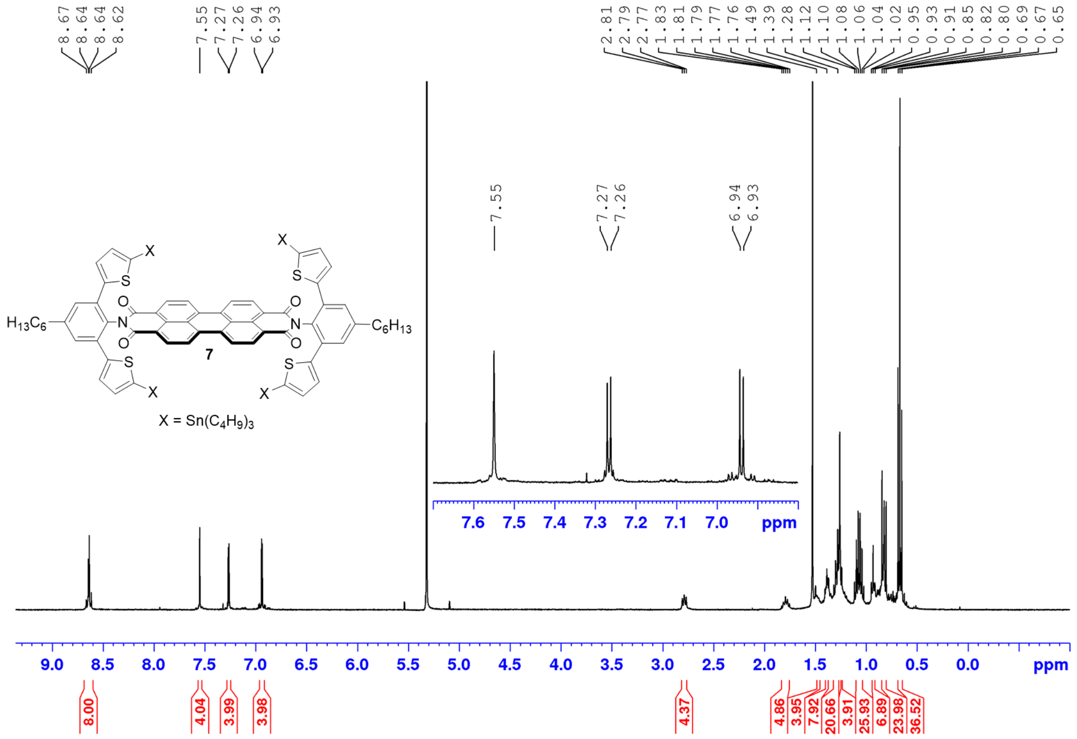

Figure S25. ^{1}H NMR spectrum of **7** in CD_2Cl_2 at 298 K.

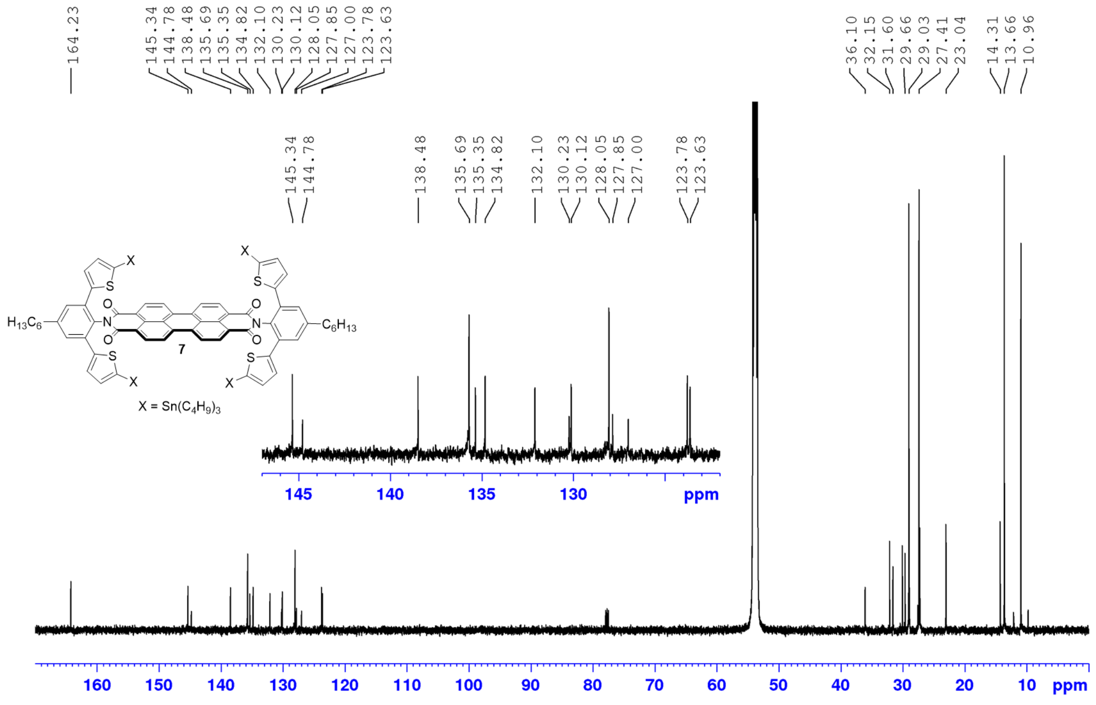

Figure S26. ^{13}C NMR spectrum of **7** in CD_2Cl_2 at 298 K.

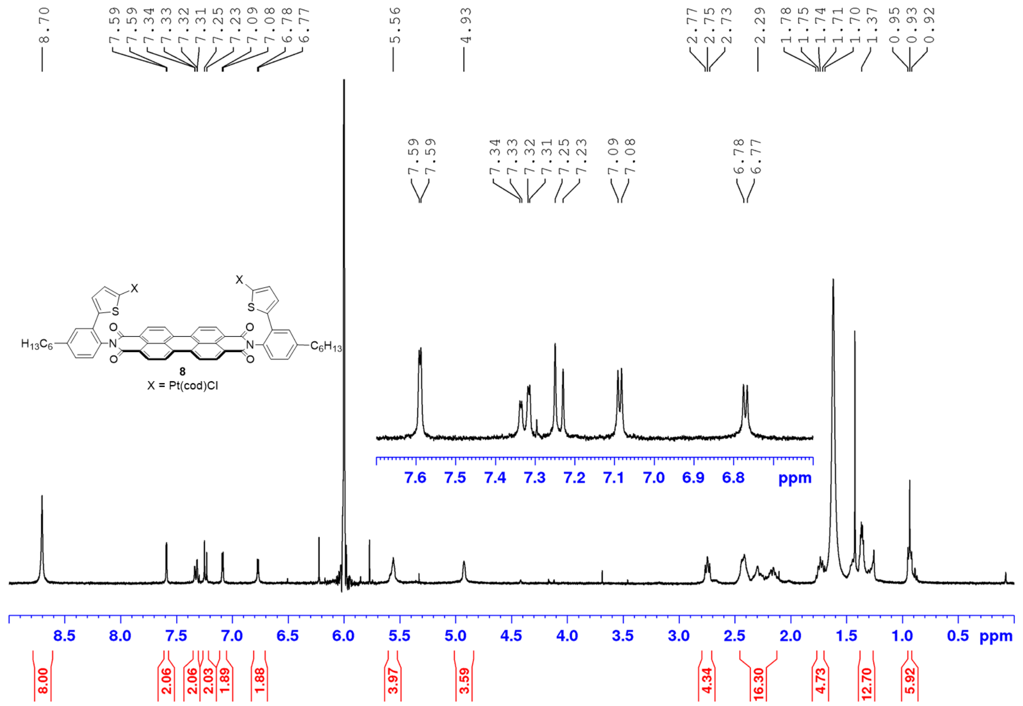

Figure S27. ^{1}H NMR spectrum of **8** in $C_2D_2Cl_4$ at 298 K.

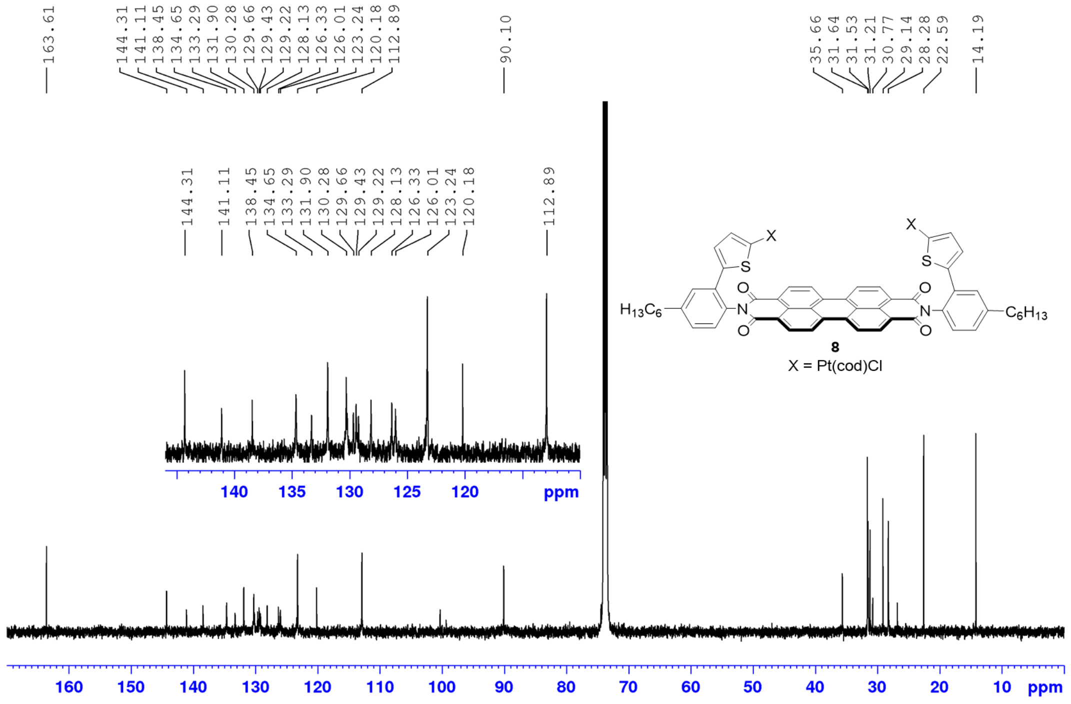

Figure S28. ^{13}H NMR spectrum of **8** in $C_2D_2Cl_4$ at 298 K.

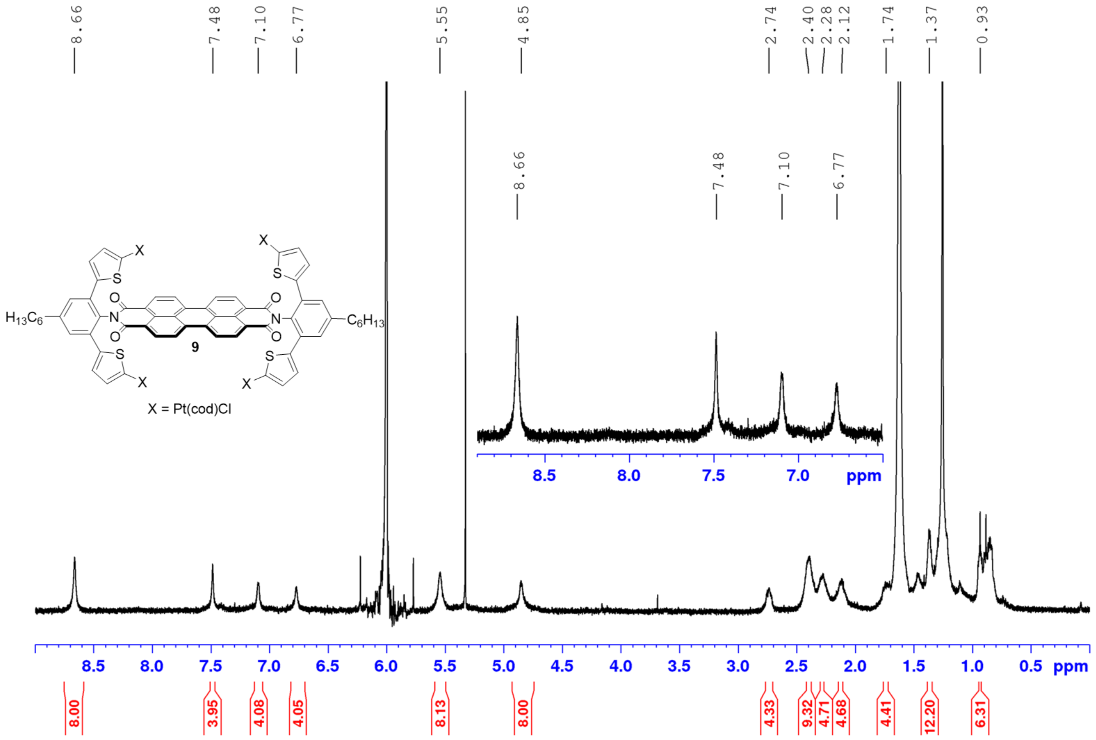

Figure S29. ^{1}H NMR spectrum of **9** in $C_2D_2Cl_4$ at 298 K.

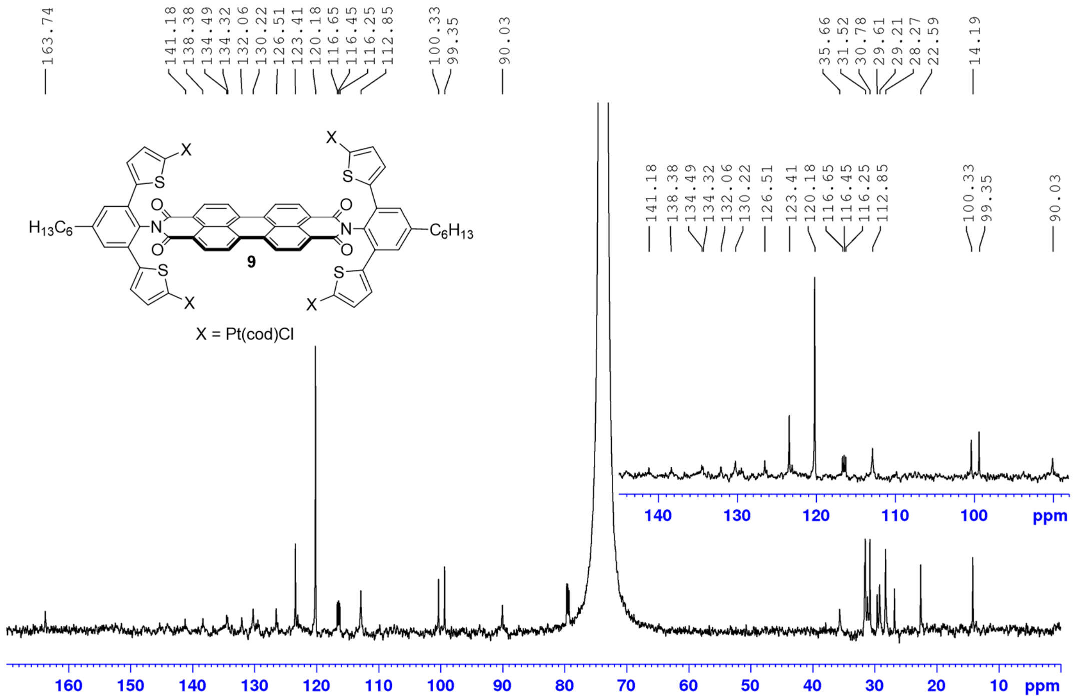

Figure S30. ^{13}C NMR spectrum of **9** in $C_2D_2Cl_4$ at 298 K. Residual signals of $CHCl_3$ (79.5 ppm), H-grease (31.1 pm) and cyclohexane (26.8 ppm).

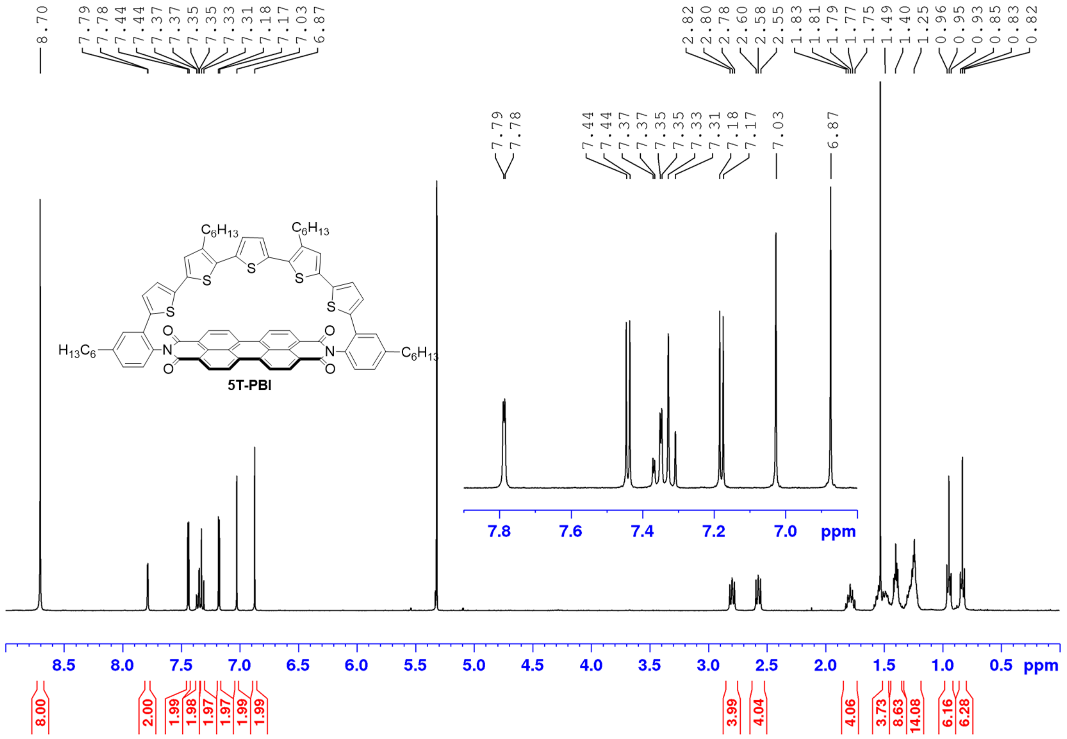

Figure S31. ^{1}H NMR spectrum of **5T-PBI** in CD_2Cl_2 at 298 K.

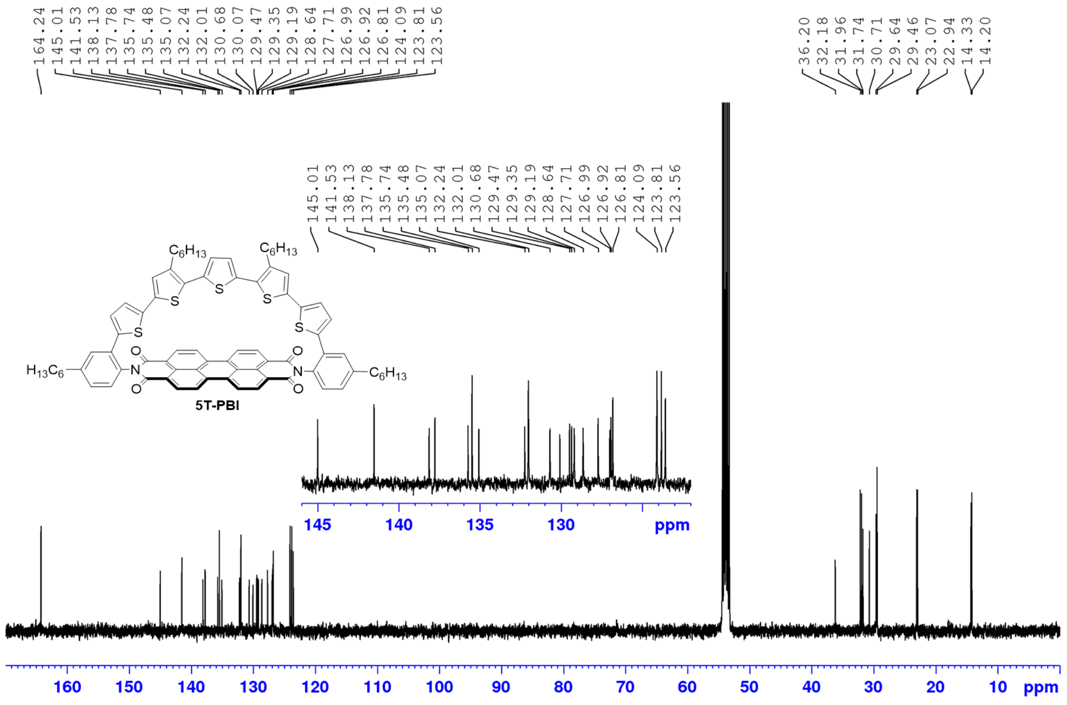

Figure S32. ^{13}C NMR spectrum of **5T-PBI** in CD_2Cl_2 at 298 K.

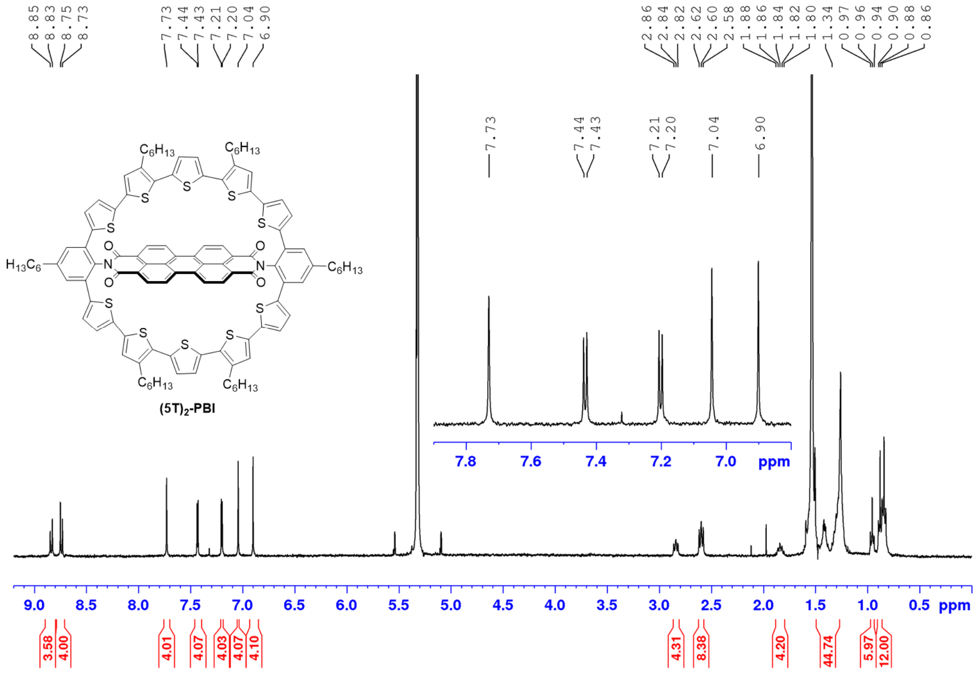

Figure S33. ^{1}H NMR spectrum of **(5T)$_2$-PBI** in CD_2Cl_2 at 298 K.

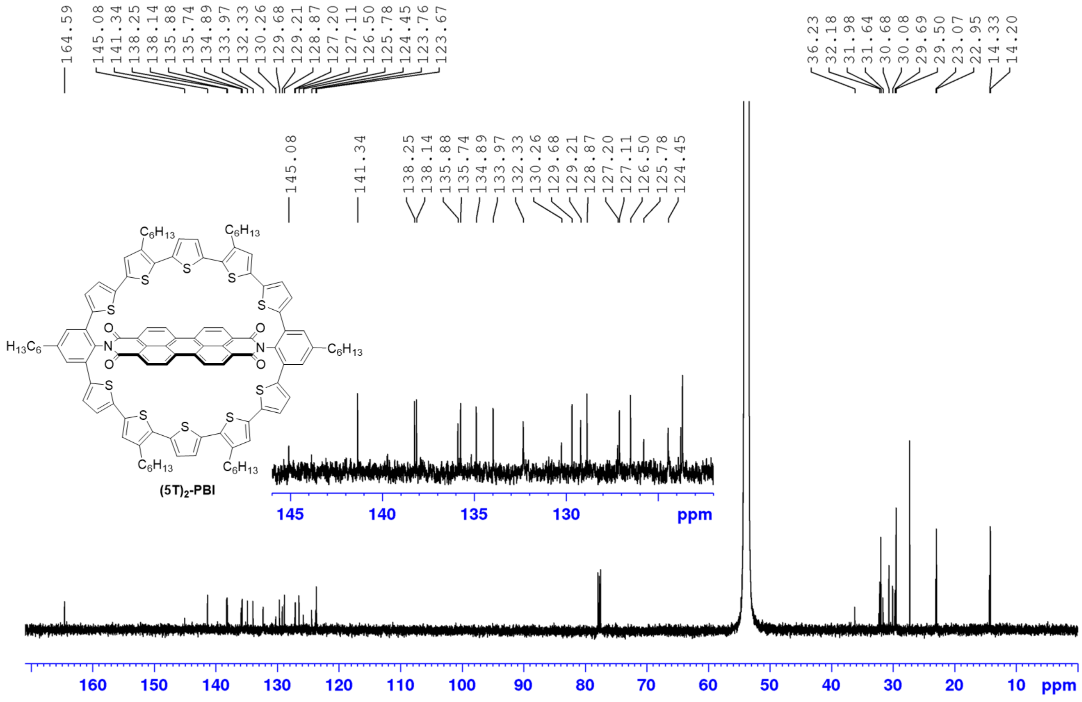

Figure S34. ^{13}C NMR spectrum of **(5T)$_2$-PBI** in CD_2Cl_2 at 298 K.

Mass Spectra

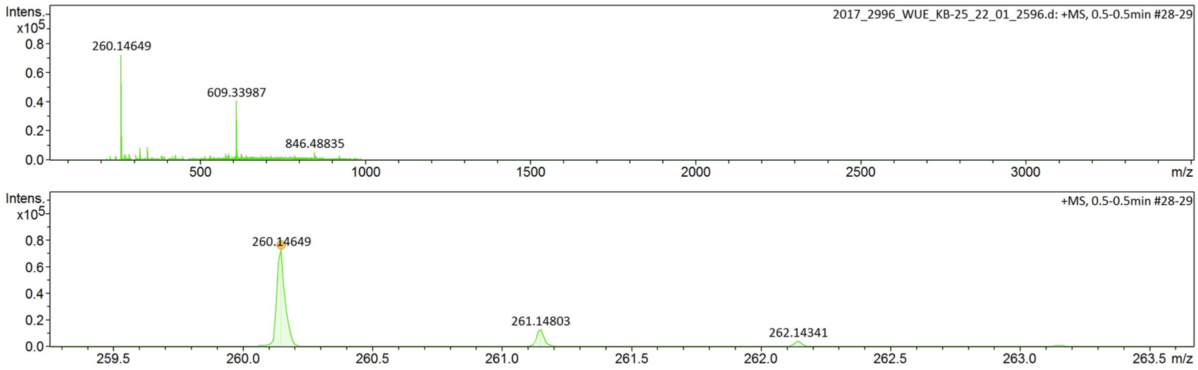

Meas. m/z	#	Ion Formula	m/z	err [ppm]	mSigma	# mSigma	Score	rdb	e⁻ Conf	N-Rule
260.14649	1	C16H22NS	260.14675	1.00	7.6	1	100.00	6.5	even	ok

Figure S35. HRMS (ESI-TOF, pos. mode, acetonitrile/chloroform 1/1) spectra of **2**.

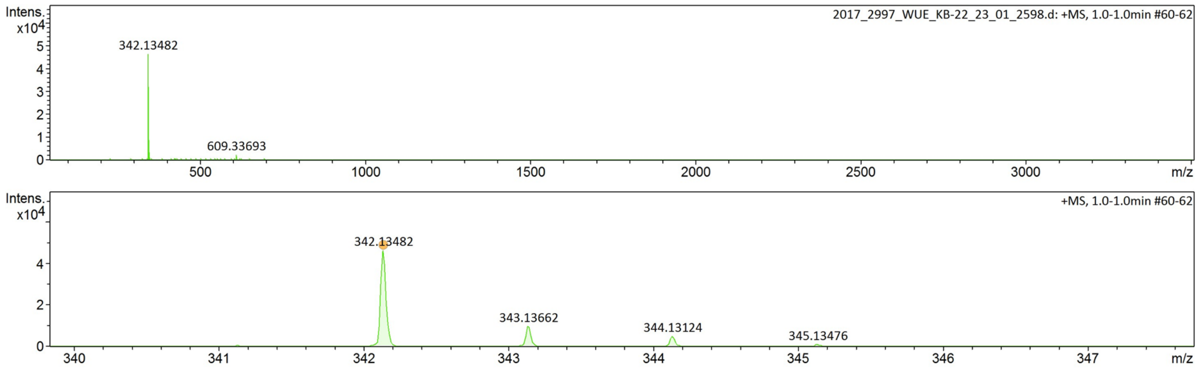

Meas. m/z	#	Ion Formula	m/z	err [ppm]	mSigma	# mSigma	Score	rdb	e⁻ Conf	N-Rule
342.13482	1	C20H24NS2	342.13447	-1.04	15.9	1	100.00	9.5	even	ok

Figure S36. HRMS (ESI-TOF, pos. mode, acetonitrile/chloroform 1/1) spectra of **3**.

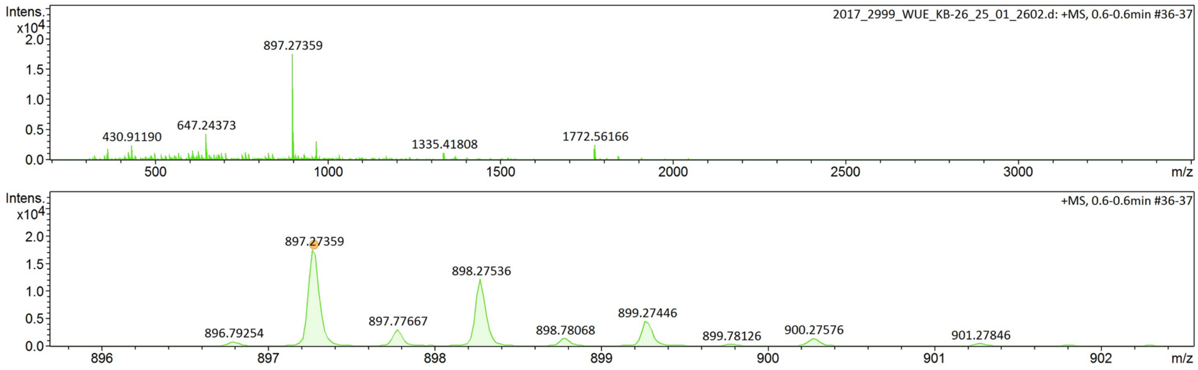

Meas. m/z	#	Ion Formula	m/z	err [ppm]	mSigma	# mSigma	Score	rdb	e⁻ Conf	N-Rule
897.27359	1	C56H46N2NaO4S2	897.27912	6.16	33.5	1	100.00	34.5	even	ok

Figure S37. HRMS (ESI-TOF, pos. mode, acetonitrile/chloroform 1/1) spectra of **4**.

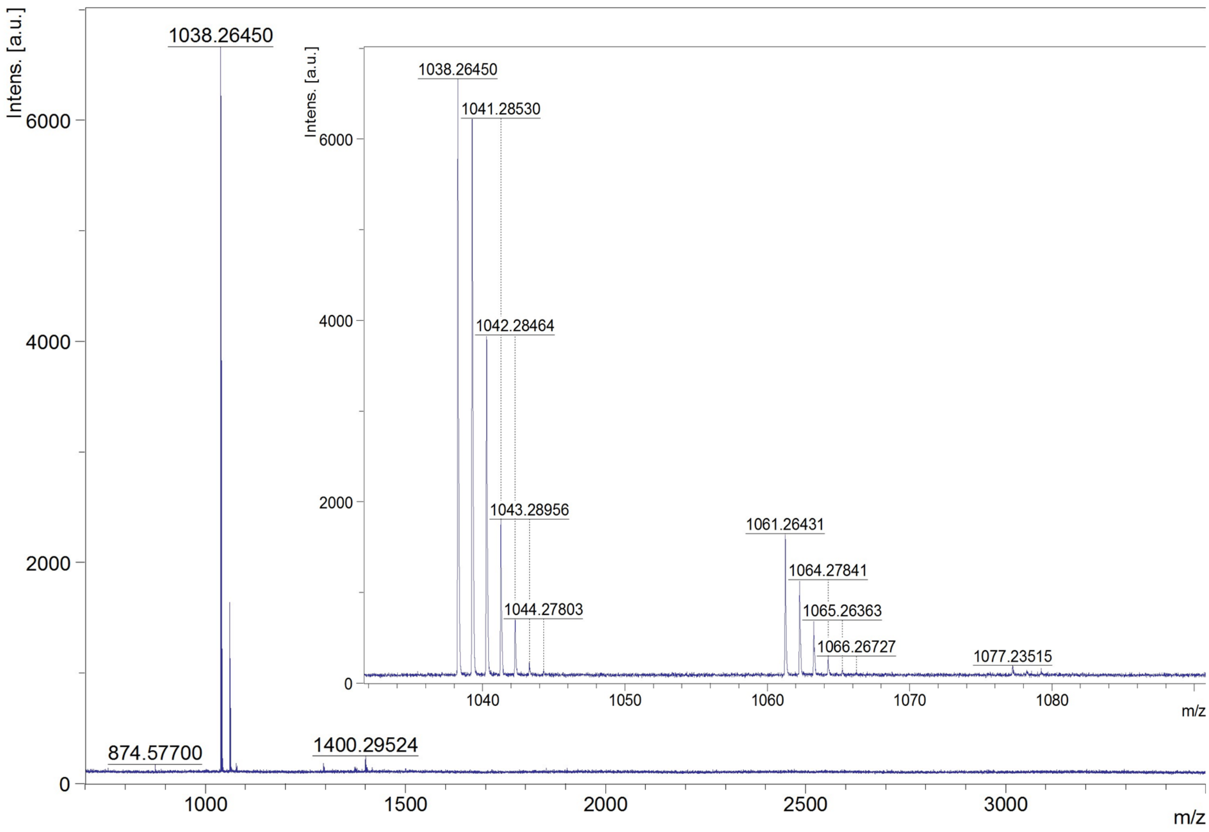

Formula	Mass	Error	mSigma	DblEq	N rule	Electron Configuration
C 64 H 50 N 2 O 4 S 4	1,038.2648	0.2864	91.7609	41.00	ok	odd

Figure S38. HRMS (MALDI-TOF, pos. mode, DCTB in $CHCl_3$) spectra of **5**.

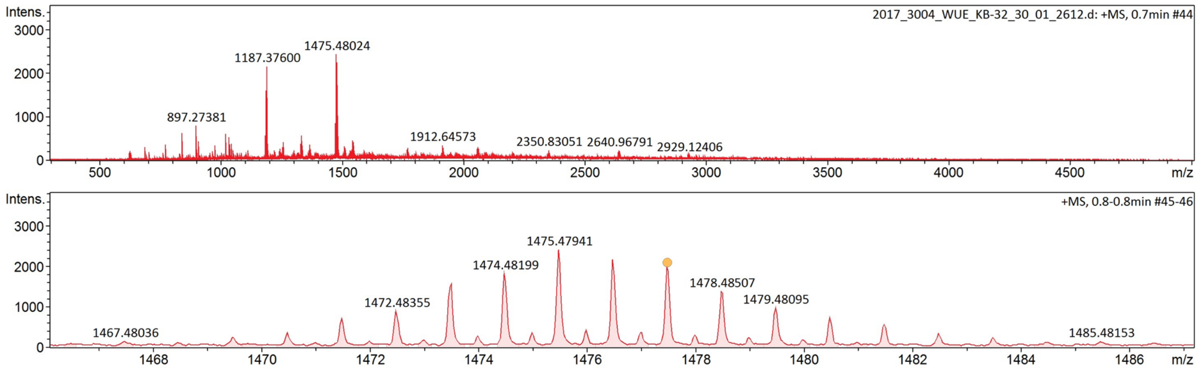

Meas. m/z	#	Ion Formula	m/z	err [ppm]	mSigma	# mSigma	Score	rdb	e⁻ Conf	N-Rule
1477.48205	1	C80H98N2NaO4S2Sn2	1477.49041	7.52	44.2	1	100.00	34.5	even	ok

Figure S39. HRMS (MALDI-TOF, pos. mode, DCTB in $CHCl_3$) spectra of **6**.

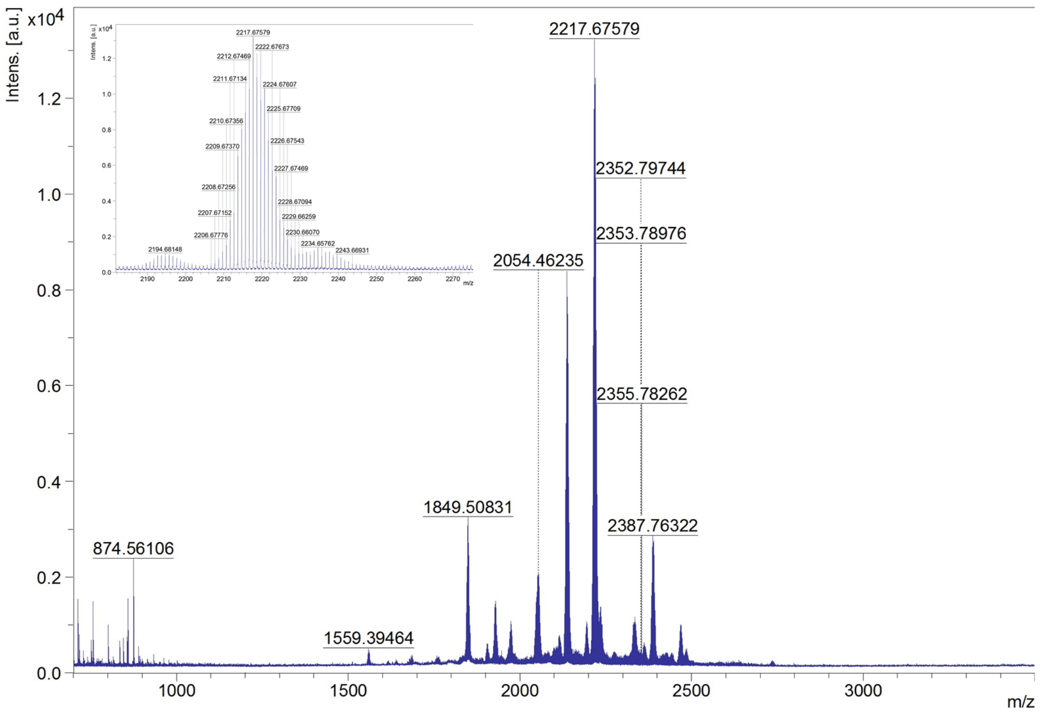

Formula	Mass	Error	mSigma	DblEq	N rule	Electron Configuration
C 112 H 154 N 2 Na O 4 S 4 Sn 4	2,221.6771	0.2617	50.9627	40.50	ok	even

Figure S40. HRMS (MALDI-TOF, pos. mode, DCTB in $CHCl_3$) spectra of **7**.

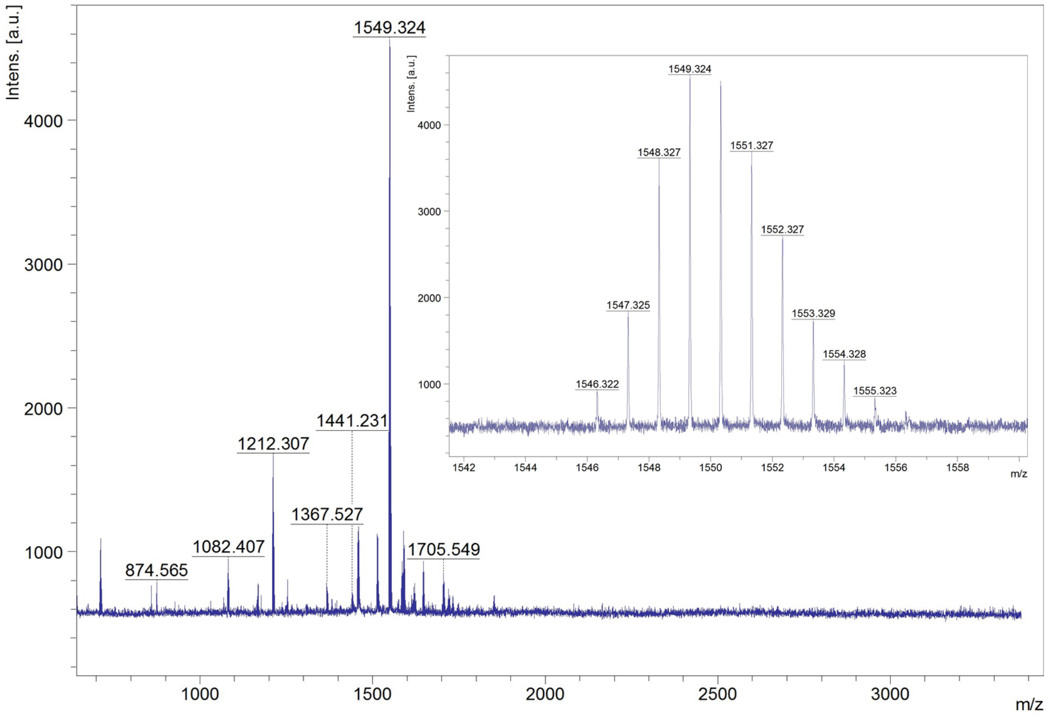

Formula	Mass	Error	mSigma	DblEq	N rule	Electron Configuration
C 72 H 68 Cl 2 N 2 O 4 Pt 2 S 2	1,548.3288	1.4653	34.7120	39.00	ok	odd

Figure S41. HRMS (MALDI-TOF, pos. mode, DCTB in $CHCl_3$) spectra of **8**.

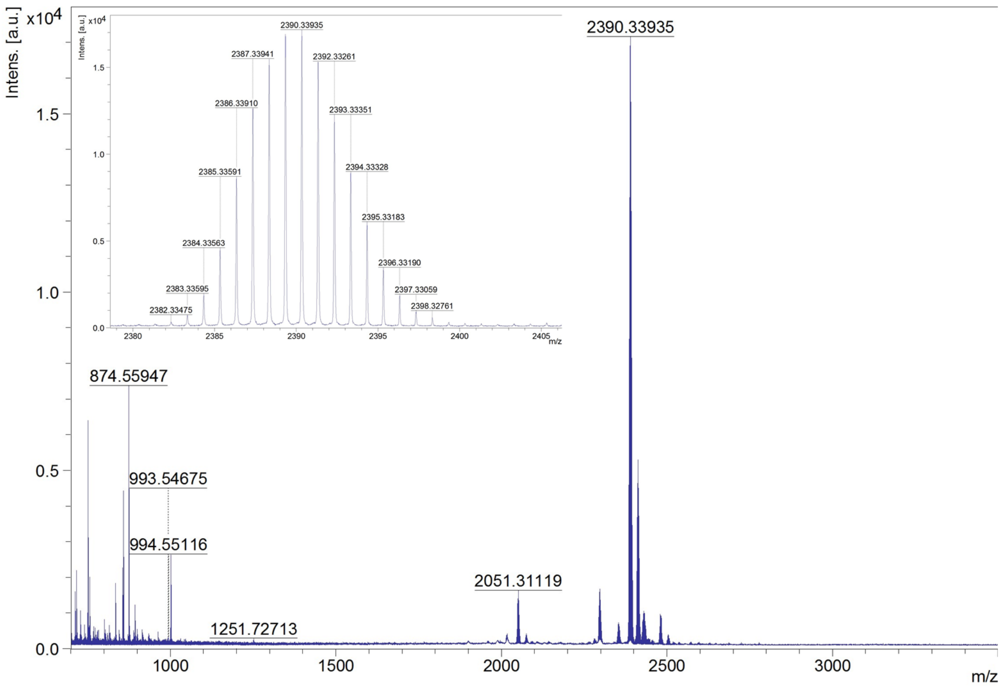

Formula	Mass	Error	mSigma	DblEq	N rule	Electron Configuration
C 96 H 94 Cl 4 N 2 O 4 Pt 4 S 4	2,386.3437	1.9164	28.3167	49.00	ok	odd

Figure S42. HRMS (MALDI-TOF, pos. mode, DCTB in $CHCl_3$) spectra of **9**.

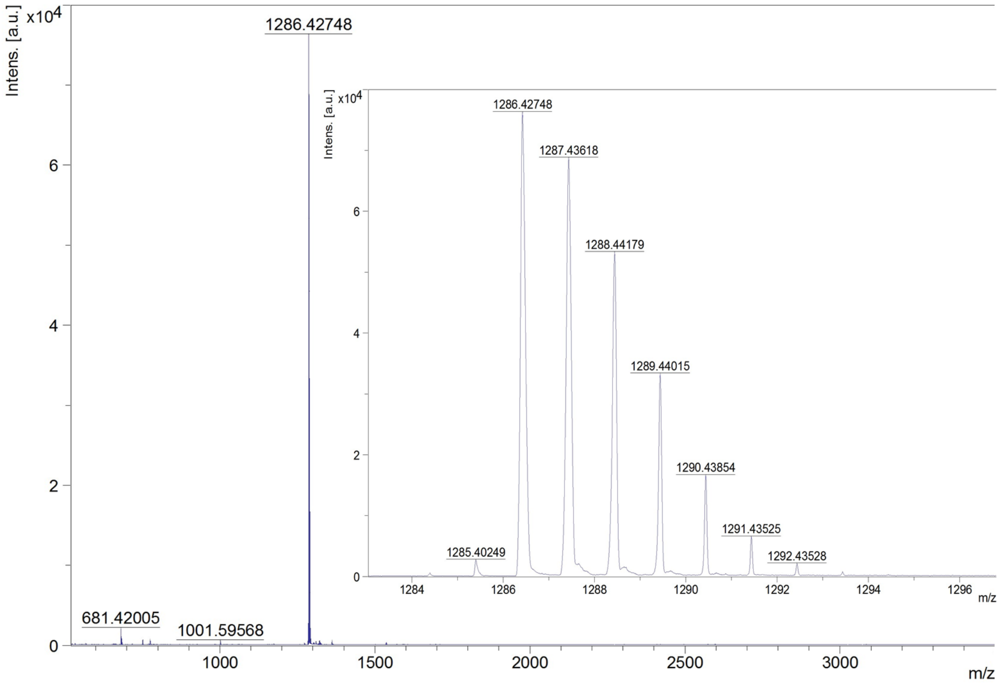

Formula	Mass	Error	mSigma	DblEq	N rule	Electron Configuration
C 80 H 74 N 2 O 4 S 5	1,286.4247	2.1853	48.1584	45.00	ok	odd

Figure S43. HRMS (MALDI-TOF, pos. mode, DCTB in $CHCl_3$) spectra of **5T-PBI**.

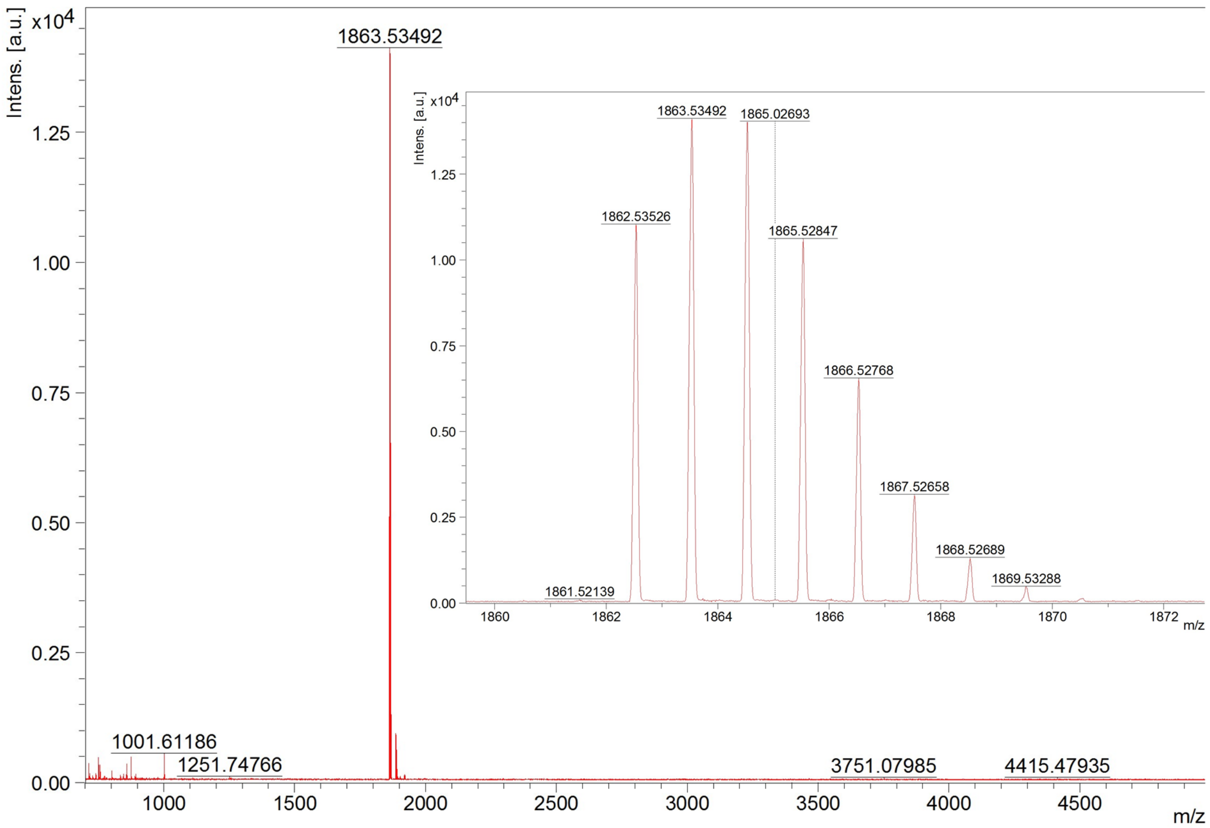

Formula	Mass	Error	mSigma	DblEq	N rule	Electron Configuration
C 112 H 106 N 2 O 4 S 10	1,862.5354	0.0846	13.9310	61.00	ok	odd

Figure S44. HRMS (MALDI-TOF, pos. mode, DCTB in $CHCl_3$) spectra of **$(5T)_2$-PBI**.

Cartesian Coordinates Received from DFT Calculations

Final geometry:

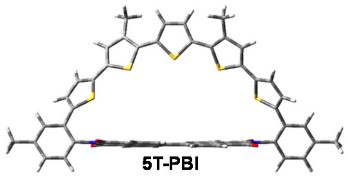

5T-PBI

Total energy: −4708.34654570 Hartrees

C1	3.0379300	-2.5972200	2.2171600
C2	1.6430100	-2.5910200	2.3218800
C3	0.8187400	-2.5326100	1.1964300
C4	1.4289000	-2.5046600	-0.0976600
C5	2.8550800	-2.4869600	-0.1948300
C6	3.6491100	-2.5346100	0.9767800
C7	0.6476300	-2.4889500	-1.2965900
C8	1.3097700	-2.4262600	-2.5242200
C9	2.7053000	-2.3863200	-2.6099900
C10	3.4804400	-2.4197700	-1.4635800
C11	5.1297100	-2.5217600	0.9018100
C12	4.9560900	-2.3587000	-1.5897800
C13	-0.6476200	-2.4888000	1.2968900
C14	-0.8187400	-2.5327500	-1.1961300
C15	-1.4288900	-2.5046500	0.0979600
C16	-2.8550700	-2.4869400	0.1951300
C17	-3.6491100	-2.5347200	-0.9764800
C18	-3.0379300	-2.5974800	-2.2168500
C19	-1.6430100	-2.5912900	-2.3215700
C20	-1.3097700	-2.4259700	2.5245100
C21	-2.7053000	-2.3860100	2.6102800
C22	-3.4804400	-2.4196000	1.4638700
C23	-4.9560900	-2.3585200	1.5900700
C24	-5.1297000	-2.5218700	-0.9015100
N25	5.6928700	-2.3926100	-0.3869500
N26	-5.6928700	-2.3925600	0.3872300
O27	-5.8410700	-2.6076100	-1.8887600
O28	-5.5217400	-2.2728500	2.6687400
O29	5.5217400	-2.2731600	-2.6684700
O30	5.8410700	-2.6073900	1.8890700
C31	-7.1342600	-2.3364100	0.4818700
C32	7.1342700	-2.3364700	-0.4815900
C33	-7.8123600	-3.4770100	0.9076600
C34	-9.1998400	-3.4902300	0.9894600
C35	-9.9424400	-2.3543600	0.6397000
C36	-9.2496200	-1.2176800	0.2228900
C37	-7.8453900	-1.1737600	0.1305100
C38	7.8453900	-1.1737800	-0.1303600
C39	9.2496200	-1.2177000	-0.2227400
C40	9.9424500	-2.3544300	-0.6394200

C41	9.1998500	-3.4903400	-0.9890400
C42	7.8123600	-3.4771100	-0.9072400
C43	-1.2445200	4.8224300	-0.2615100
C44	-0.6942000	6.0856000	-0.1413600
C45	0.6942000	6.0856100	0.1406200
C46	1.2445100	4.8224700	0.2609200
S47	-0.0000020	3.6072400	-0.0002190
S48	3.1870500	2.8454200	0.0661100
C49	2.6052000	4.4302100	0.5582600
C50	3.5979700	5.1285600	1.2354700
C51	4.8031100	4.3826800	1.3429500
C52	4.7596400	3.1238100	0.7810500
S53	5.7834400	0.7714300	-0.3502600
C54	5.7921200	2.1094100	0.7823500
C55	6.8759700	2.0051100	1.6310700
C56	7.6772200	0.8626000	1.3869600
C57	7.2203200	0.0635900	0.3611200
C58	-4.8031200	4.3825200	-1.3434800
C59	-3.5979700	5.1284100	-1.2360900
C60	-2.6052100	4.4301500	-0.5588000
S61	-3.1870500	2.8454200	-0.0664600
C62	-4.7596400	3.1237100	-0.7814300
C63	-7.6772300	0.8624300	-1.3870600
C64	-6.8759800	2.0049200	-1.6313000
C65	-5.7921300	2.1093200	-0.7826000
S66	-5.7834400	0.7714700	0.3501600
C67	-7.2203200	0.0635500	-0.3611300
C68	11.4512200	-2.3627100	-0.7163300
C69	-11.4512100	-2.3626400	0.7166200
C70	-3.4443900	6.5053400	-1.8253500
C71	3.4443900	6.5055500	1.8245700
H72	3.6621800	-2.6416200	3.1031900
H73	1.2096400	-2.6331000	3.3140400
H74	0.7446200	-2.3939900	-3.4480400
H75	3.2019800	-2.3250900	-3.5724300
H76	-3.6621800	-2.6419800	-3.1028800
H77	-1.2096400	-2.6334900	-3.3137200
H78	-0.7446200	-2.3935800	3.4483200
H79	-3.2019700	-2.3246700	3.5727100
H80	-9.7093400	-4.3884700	1.3294000
H81	-9.8062600	-0.3175300	-0.0218800
H82	9.8062600	-0.3175200	0.0219200
H83	9.7093400	-4.3886200	-1.3288800
H84	-1.2768500	6.9938100	-0.2300500
H85	1.2768500	6.9938400	0.2292000
H86	5.6904800	4.7735900	1.8303300
H87	7.0608600	2.7068400	2.4368700
H88	8.5440500	0.6004700	1.9836000
H89	-5.6904900	4.7733700	-1.8308900
H90	-8.5440600	0.6002400	-1.9836600

H91	-7.0608700	2.7065500	-2.4371800
H92	11.8663000	-1.3632600	-0.5542300
H93	11.8831300	-3.0292000	0.0412000
H94	11.7992200	-2.7176600	-1.6935100
H95	-11.8831200	-3.0292100	-0.0408300
H96	-11.7992100	-2.7174700	1.6938400
H97	-11.8663000	-1.3632000	0.5544100
H98	-4.1968100	6.6777100	-2.6014600
H99	-3.5711300	7.2918800	-1.0693100
H100	-2.4547000	6.6412500	-2.2745000
H101	2.4546800	6.6415200	2.2736900
H102	4.1967900	6.6780200	2.6006700
H103	3.5711300	7.2920100	1.0684400
H104	7.2379700	-4.3591700	-1.1720700
H105	-7.2379700	-4.3590300	1.1725900

Final geometry:

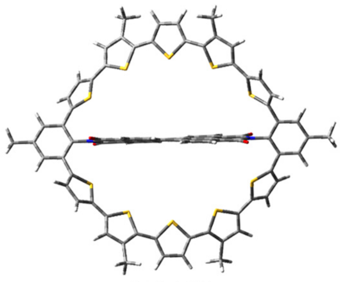

$(5T)_2$-PBI

Total energy: −7544.84772028 Hartrees

C1	-2.8754100	-0.1459000	-2.4185000
C2	-1.4773400	-0.1128900	-2.4281000
C3	-0.7341900	-0.0306600	-1.2490700
C4	-1.4317400	-0.0009190	-0.0001740
C5	-2.8607500	-0.0008510	-0.0003510
C6	-3.5706500	-0.0803700	-1.2232800
C7	-0.7344900	0.0287800	1.2488900
C8	-1.4779100	0.1110600	2.4277400
C9	-2.8759800	0.1442100	2.4178000
C10	-3.5709200	0.0787600	1.2224100
C11	-5.0501900	-0.1201900	-1.2474300
C12	-5.0504600	0.1188600	1.2462100
C13	0.7344800	0.0285200	-1.2488900
C14	0.7341800	-0.0304000	1.2490800
C15	1.4317400	-0.0009200	0.0001770
C16	2.8607400	-0.0008530	0.0003540
C17	3.5706400	-0.0801200	1.2233000
C18	2.8754100	-0.1454000	2.4185300
C19	1.4773300	-0.1123800	2.4281300
C20	1.4779100	0.1105500	-2.4277700
C21	2.8759700	0.1437100	-2.4178300
C22	3.5709200	0.0785000	-1.2224200
C23	5.0504500	0.1186100	-1.2462300

C24	5.0501900	-0.1199400	1.2474600
N25	-5.7018600	-0.0006340	-0.0007020
N26	5.7018600	-0.0006390	0.0007050
O27	5.6947500	-0.2537200	2.2753800
O28	5.6952200	0.2526100	-2.2739700
O29	-5.6952300	0.2530800	2.2739200
O30	-5.6947600	-0.2541800	-2.2753200
C31	7.1450100	-0.0009150	0.0011800
C32	-7.1450100	-0.0009070	-0.0011700
C33	7.8400100	-1.1625100	-0.3883500
C34	9.2437300	-1.1357700	-0.3823900
C35	9.9604500	-0.0009390	-0.0007000
C36	9.2442500	1.1356500	0.3766100
C37	7.8403900	1.1615300	0.3873500
C38	-7.8403900	1.1614600	-0.3875900
C39	-9.2442500	1.1355800	-0.3768500
C40	-9.9604500	-0.0009300	0.0006940
C41	-9.2437300	-1.1356900	0.3826100
C42	-7.8400100	-1.1624300	0.3885800
C43	1.2247100	7.1553700	0.3419900
C44	0.6834800	8.4183500	0.1856500
C45	-0.6834800	8.4183100	-0.1872800
C46	-1.2247000	7.1553000	-0.3433900
S47	0.0000060	5.9400300	-0.0005890
S48	-3.1850700	5.1863100	-0.2682000
C49	-2.5633500	6.7643000	-0.7319500
C50	-3.5024100	7.4597600	-1.4844600
C51	-4.7002200	6.7168100	-1.6711100
C52	-4.7017800	5.4634900	-1.0962100
S53	-5.8346700	3.1473900	0.0022900
C54	-5.7305300	4.4467500	-1.1691900
C55	-6.7179000	4.3061200	-2.1233700
C56	-7.5318300	3.1619700	-1.9292000
C57	-7.1778400	2.3986800	-0.8381800
C58	4.7002200	6.7171300	1.6698000
C59	3.5024100	7.4600500	1.4830100
C60	2.5633600	6.7644400	0.7306300
S61	3.1850800	5.1863700	0.2671700
C62	4.7017900	5.4637000	1.0951300
C63	7.5318400	3.1623500	1.9285700
C64	6.7179100	4.3065400	2.1225100
C65	5.7305400	4.4469800	1.1683100
S66	5.8346700	3.1473900	-0.0029100
C67	7.1778400	2.3988500	0.8376900
C68	-11.4710000	-0.0108100	-0.0276300
C69	11.4710000	-0.0108200	0.0276700
C70	3.3014400	8.8310100	2.0719800
C71	-3.3014400	8.8306100	-2.0736900
H72	-3.4366800	-0.2197800	-3.3437800
H73	-0.9753500	-0.1628400	-3.3869500

H74	-0.9761500	0.1609700	3.3867200
H75	-3.4374700	0.2181800	3.3429400
H76	3.4366700	-0.2190800	3.3438300
H77	0.9753500	-0.1621300	3.3869900
H78	0.9761500	0.1602600	-3.3867500
H79	3.4374700	0.2174800	-3.3429800
H80	9.7779900	-2.0373700	-0.6680200
H81	9.7788500	2.0398800	0.6529900
H82	-9.7788500	2.0397500	-0.6534200
H83	-9.7779900	-2.0372300	0.6684200
H84	1.2592400	9.3265100	0.3125200
H85	-1.2592400	9.3264400	-0.3143200
H86	-5.5492200	7.1054300	-2.2243200
H87	-6.8188500	4.9788000	-2.9679200
H88	-8.3274800	2.8705300	-2.6061600
H89	5.5492200	7.1058600	2.2229400
H90	8.3275000	2.8710500	2.6055800
H91	6.8188700	4.9793900	2.9669200
H92	-11.8803000	0.9894800	0.1475700
H93	-11.8454300	-0.3516300	-1.0019200
H94	-11.8814700	-0.6838200	0.7322900
H95	11.8453900	-0.3511700	1.0021400
H96	11.8814800	-0.6842100	-0.7319100
H97	11.8803200	0.9893800	-0.1480000
H98	3.9897900	8.9957000	2.9070300
H99	3.4872200	9.6251000	1.3362700
H100	2.2790900	8.9623100	2.4422800
H101	-2.2790800	8.9618500	-2.4440200
H102	-3.9897800	8.9951400	-2.9087800
H103	-3.4872200	9.6248300	-1.3381300
C104	-7.1761600	-2.3975400	0.8434600
S105	-5.8372800	-3.1504100	-0.0000470
C106	-7.5251800	-3.1554900	1.9396700
C107	-5.7278300	-4.4443600	1.1770100
C108	-6.7105100	-4.2990000	2.1352400
H109	-8.3174900	-2.8605900	2.6190500
C110	-4.6994700	-5.4615000	1.1036500
H111	-6.8074500	-4.9677000	2.9834200
S112	-3.1848600	-5.1858500	0.2713000
C113	-4.6964300	-6.7136400	1.6810500
C114	-2.5619100	-6.7628500	0.7366400
C115	-3.4989800	-7.4569000	1.4928400
H116	-5.5440900	-7.1011900	2.2370800
C117	-1.2240300	-7.1542100	0.3456500
C118	-3.2962900	-8.8266200	2.0841000
C119	-0.6831600	-8.4172200	0.1885800
S120	-0.0000040	-5.9389400	0.0005770
H121	-2.2724800	-8.9575700	2.4504900
H122	-3.9813000	-8.9891300	2.9223200
H123	-3.4854400	-9.6222600	1.3509200

C124	0.6831500	-8.4172600	-0.1869400
H125	-1.2587700	-9.3253100	0.3167400
C126	1.2240200	-7.1542800	-0.3442600
H127	1.2587600	-9.3253700	-0.3149200
C128	2.5619000	-6.7630000	-0.7353200
C129	3.4989800	-7.4572000	-1.4913800
S130	3.1848600	-5.1859100	-0.2702900
C131	4.6964300	-6.7139800	-1.6797300
C132	3.2962800	-8.8270400	-2.0823700
C133	4.6994700	-5.4617200	-1.1025800
H134	5.5440900	-7.1016400	-2.2356800
H135	3.9813000	-8.9897100	-2.9205500
H136	3.4854300	-9.6225300	-1.3490300
H137	2.2724800	-8.9580600	-2.4487400
C138	5.7278300	-4.4446000	-1.1761400
C139	6.7105200	-4.2994400	-2.1343800
S140	5.8372600	-3.1504000	0.0006570
C141	7.5251900	-3.1558900	-1.9390300
H142	6.8074800	-4.9683100	-2.9824200
C143	7.1761500	-2.3977100	-0.8429800
H144	8.3175100	-2.8611400	-2.6184700

Final geometry:

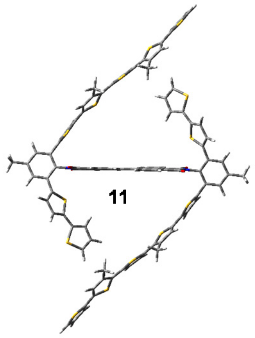

11

Total energy: –9754.49276862 Hartrees

C1	0.95871	-2.71421	-2.56024
C2	0.51661	-1.38751	-2.56859
C3	0.24216	-0.69402	-1.3882
C4	0.4409	-1.36277	-0.1387
C5	0.87715	-2.72411	-0.13926
C6	1.1322	-3.38817	-1.36398
C7	0.21252	-0.70384	1.11108
C8	0.40472	-1.42547	2.29081
C9	0.81396	-2.76296	2.28128
C10	1.05364	-3.41505	1.08438
C11	1.58437	-4.79821	-1.39089
C12	1.46155	-4.8392	1.10759
C13	-0.24255	0.69396	-1.3882
C14	-0.21281	0.70382	1.11108
C15	-0.44122	1.36274	-0.13871

C16	-0.87741	2.7241	-0.13927
C17	-1.0538	3.41507	1.08437
C18	-0.81411	2.76299	2.28127
C19	-0.40493	1.42548	2.29081
C20	-0.51706	1.38743	-2.56858
C21	-0.9591	2.71415	-2.56024
C22	-1.1325	3.38814	-1.36399
C23	-1.58457	4.79822	-1.39089
C24	-1.46159	4.83925	1.10758
N25	1.70772	-5.44122	-0.14375
N26	-1.70777	5.44126	-0.14377
O27	-1.57246	5.48079	2.13996
O28	-1.84329	5.39108	-2.42724
O29	1.57249	-5.48071	2.13999
O30	1.84306	-5.39107	-2.42724
C31	-2.03734	6.84718	-0.14897
C32	2.03742	-6.8471	-0.14899
C33	-1.00602	7.7706	-0.39203
C34	-1.29934	9.13746	-0.38794
C35	-2.59432	9.60371	-0.13747
C36	-3.59832	8.66518	0.09794
C37	-3.35116	7.28051	0.09949
C38	1.00619	-7.77062	-0.39214
C39	1.29968	-9.13744	-0.38829
C40	2.59475	-9.60357	-0.13795
C41	3.59862	-8.66496	0.09763
C42	3.35128	-7.28031	0.0994
C43	-9.56894	-2.65015	-0.16738
C44	-9.14967	-3.03108	-1.4309
C45	-7.99508	-3.84264	-1.43564
C46	-7.48542	-4.10944	-0.17662
S47	-8.48308	-3.32469	1.04094
S48	-5.06813	-5.05809	-1.1052
C49	-6.2809	-4.83962	0.15355
C50	-5.86642	-5.43159	1.34004
C51	-4.60028	-6.06716	1.21144
C52	-4.03002	-5.98035	-0.03904
S53	-1.45717	-6.82406	0.62588
C54	-2.75963	-6.49492	-0.50441
C55	-2.35039	-6.75894	-1.79412
C56	-1.00953	-7.22358	-1.88072
C57	-0.37949	-7.31839	-0.66523
C58	-12.32501	-0.51891	1.22699
C59	-11.06539	-1.17284	1.33167
C60	-10.74404	-1.88072	0.17949
S61	-12.03979	-1.75496	-1.00756
C62	-5.42538	6.55805	1.37624
C63	-6.43032	5.55739	1.39935
S64	-4.86363	4.95543	-0.55699
C65	-4.47902	6.3794	0.39232

C66	2.89148	-11.08473	-0.11626
C67	-2.89088	11.08491	-0.11536
C68	-10.20018	-1.05286	2.55991
C69	-6.64122	-5.44927	2.63239
H70	1.16789	-3.23723	-3.48728
H71	0.39292	-0.90131	-3.52885
H72	0.23029	-0.95679	3.25192
H73	0.94793	-3.31063	3.20801
H74	-0.948	3.31068	3.208
H75	-0.23048	0.9568	3.25192
H76	-0.39344	0.9012	-3.52884
H77	-1.16832	3.23715	-3.48728
H78	-0.49585	9.84259	-0.58281
H79	-4.61693	9.00367	0.26397
H80	0.49628	-9.84265	-0.58319
H81	4.61727	-9.00334	0.26362
H82	-9.66794	-2.72943	-2.33492
H83	-7.54371	-4.22903	-2.34315
H84	-4.1323	-6.60533	2.02995
H85	-2.99969	-6.63404	-2.65442
H86	-0.50912	-7.46306	-2.81222
H87	-12.7242	0.1038	2.02177
H88	-5.37169	7.37131	2.09184
H89	-7.22762	5.52323	2.13422
H90	2.54746	-11.54272	0.82023
H91	2.38488	-11.606	-0.93604
H92	3.96499	-11.27884	-0.20335
H93	-2.54828	11.54226	0.82197
H94	-2.3829	11.6066	-0.93401
H95	-3.96423	11.27922	-0.20399
H96	-10.58985	-0.27564	3.22445
H97	-10.16968	-1.98803	3.13314
H98	-9.16748	-0.78914	2.30662
H99	-7.69823	-5.68883	2.4736
H100	-6.22605	-6.19808	3.31406
H101	-6.60138	-4.48086	3.14771
C102	4.47904	-6.37912	0.39237
S103	4.8634	-4.95483	-0.55655
C104	5.4255	-6.55791	1.37619
C105	6.4303	-5.55712	1.3995
H106	5.37196	-7.37136	2.09158
H107	7.22765	-5.52308	2.13432
S108	12.03952	1.75412	-1.00799
C109	12.32566	0.5195	1.22724
C110	10.74416	1.88044	0.17943
C111	11.06599	1.17332	1.33193
H112	12.72519	-0.10265	2.02228
C113	9.56885	2.6495	-0.16754
C114	10.20118	1.05398	2.56052
C115	9.1488	3.02909	-1.4312

S116	8.48375	3.32533	1.04074
H117	9.16831	0.79055	2.30769
H118	10.59084	0.27683	3.22515
H119	10.17122	1.98934	3.13348
C120	7.99418	3.84061	-1.43609
H121	9.6665	2.72647	-2.33522
C122	7.48528	4.10873	-0.17703
H123	7.54223	4.226	-2.34374
C124	6.28096	4.83926	0.1531
C125	5.8669	5.4319	1.3394
S126	5.06784	5.05724	-1.10541
C127	4.60081	6.06757	1.21084
C128	6.64206	5.45013	2.63153
C129	4.03016	5.98018	-0.03942
H130	4.13313	6.6062	2.02922
H131	6.22671	6.19884	3.31321
H132	6.60284	4.48178	3.147
H133	7.69891	5.69019	2.47241
C124	2.75969	6.49463	-0.50469
C135	2.35011	6.75795	-1.79443
S136	1.45757	6.82449	0.62578
C137	1.00926	7.22264	-1.88093
H138	2.99917	6.63254	-2.65484
C139	0.37957	7.31819	-0.66532
H140	0.50861	7.46162	-2.81243
C141	-14.2592	-0.18225	-0.4033
C142	-14.77335	-0.09534	-1.68033
S143	-15.41243	0.4595	0.75702
C144	-16.07116	0.4863	-1.73296
H145	-14.22464	-0.42141	-2.55759
C146	-16.54515	0.8429	-0.50036
H147	-16.62456	0.6399	-2.65295
H148	-17.49017	1.30623	-0.25098
C149	-7.09219	3.43258	0.14921
C150	-6.74985	2.25473	-0.47981
S151	-8.77987	3.38925	0.63507
C152	-7.8214	1.31988	-0.56121
H153	-5.74932	2.05814	-0.85046
C154	-8.97783	1.78498	0.00314
H155	-7.73402	0.33675	-1.01094
H156	-9.93316	1.28411	0.0865
C157	7.09187	-3.43193	0.14986
C158	6.74945	-2.25402	-0.479
S159	8.77952	-3.38849	0.63583
C160	7.82091	-1.31904	-0.5602
H161	5.74892	-2.05748	-0.8497
C162	8.97735	-1.78411	0.00416
H163	7.73347	-0.33586	-1.00981
H164	9.93261	-1.28315	0.08767
C165	6.27316	-4.59751	0.42265

C166	-6.27337	4.59802	0.42223
C167	-12.98258	-0.70718	0.03077
C168	14.25936	0.18208	-0.40348
C169	14.77352	0.09539	-1.68053
S170	15.41245	-0.46018	0.7567
C171	16.07123	-0.48646	-1.73328
H172	14.22492	0.42181	-2.55772
C173	16.54514	-0.84345	-0.50076
H174	16.62463	-0.6399	-2.6533
H175	17.49007	-1.307	-0.25146
C176	12.98282	0.70714	0.03068

Final geometry:

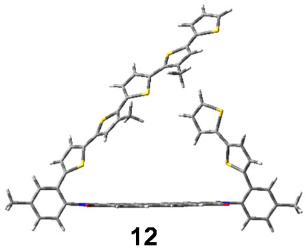

12

Total energy: –5813.16854119 Hartrees

C1	0.21155	5.03336	2.21321
C2	1.42403	4.3359	2.22778
C3	2.0176	3.86163	1.05631
C4	1.35849	4.10387	-0.1908
C5	0.11958	4.81776	-0.19664
C6	-0.44424	5.27602	1.01895
C7	1.90607	3.6475	-1.43197
C8	1.20926	3.9224	-2.61034
C9	-0.00184	4.62223	-2.60752
C10	-0.55118	5.06888	-1.41836
C11	-1.72666	6.0215	1.03876
C12	-1.841	5.79954	-1.44974
C13	3.28697	3.11894	1.06327
C14	3.17326	2.90128	-1.42458
C15	3.82789	2.65121	-0.1766
C16	5.0511	1.91085	-0.1679
C17	5.60316	1.43389	-1.38161
C18	4.95879	1.69592	-2.57805
C19	3.7619	2.41939	-2.59539
C20	3.98478	2.84527	2.2414
C21	5.18015	2.11902	2.24136
C22	5.71477	1.64724	1.05511
C23	6.96356	0.84936	1.09407
C24	6.85936	0.64647	-1.39696
N25	-2.35424	6.22396	-0.20857
N26	7.44781	0.37892	-0.14211
O27	7.36814	0.23635	-2.42707
O28	7.54641	0.58686	2.13497

O29	-2.44515	6.02502	-2.48681
O30	-2.22478	6.44743	2.06756
C31	8.6396	-0.43938	-0.13325
C32	-3.59053	6.97454	-0.21385
C33	9.88077	0.18714	-0.05074
C34	11.05342	-0.55958	-0.09127
C35	11.00642	-1.95356	-0.22507
C36	9.75415	-2.56705	-0.29641
C37	8.5529	-1.83822	-0.25025
C38	-4.8202	6.36513	0.09139
C39	-5.96416	7.18511	0.11192
C40	-5.91534	8.55409	-0.15391
C41	-4.6737	9.12323	-0.46657
C42	-3.52652	8.33858	-0.49216
C43	-0.08954	-5.10065	0.33072
C44	-0.40973	-6.06761	-0.60532
C45	-1.79094	-6.34613	-0.69512
C46	-2.57609	-5.59999	0.16703
S47	-1.55614	-4.51321	1.10046
S48	-4.95993	-6.31628	-1.01777
C49	-4.01626	-5.62925	0.30212
C50	-4.85019	-5.19765	1.32704
C51	-6.22224	-5.45155	1.04622
S52	-4.31141	3.6417	-0.55495
C53	-5.76734	2.99678	1.47194
C54	-5.74329	4.41494	1.4386
C55	-4.98482	4.93834	0.41593
C56	3.07108	-3.57779	1.65875
C57	1.71112	-3.97014	1.79506
C58	1.21604	-4.55588	0.63765
S59	2.44593	-4.58372	-0.62241
C60	3.6336	-3.8418	0.42892
C61	6.94679	-3.52268	-1.2948
C62	5.65099	-4.07457	-1.1208
C63	4.97143	-3.55382	-0.03913
S64	5.9607	-2.35792	0.77846
C65	7.27767	-2.56862	-0.36002
C66	-7.16559	9.40098	-0.10837
C67	12.27721	-2.76794	-0.29075
C68	0.94795	-3.77677	3.08035
C69	-4.40575	-4.5542	2.61568
H70	-0.23502	5.39283	3.13415
H71	1.89801	4.16683	3.18726
H72	1.59838	3.58987	-3.5652
H73	-0.53109	4.82287	-3.53296
H74	5.39752	1.32374	-3.49768
H75	3.29151	2.5948	-3.55544
H76	3.60335	3.19085	3.19476
H77	5.7026	1.90716	3.16819
H78	12.0136	-0.05443	-0.02079

H79	9.69342	-3.64926	-0.37128
H80	-6.92213	6.71874	0.32363
H81	-4.60403	10.18466	-0.69135
H82	0.3429	-6.58438	-1.19108
H83	-2.20337	-7.10041	-1.35668
H84	-7.0128	-5.21296	1.75115
H85	-6.28506	2.42199	2.2326
H86	-6.24043	5.04186	2.17084
H87	3.63232	-3.12977	2.47304
H88	7.61429	-3.79672	-2.1045
H89	5.23052	-4.83559	-1.76987
H90	-8.06577	8.783	-0.03602
H91	-7.15561	10.07583	0.75726
H92	-7.25694	10.02679	-1.00375
H93	12.80193	-2.60718	-1.24134
H94	12.97116	-2.49086	0.51127
H95	12.07162	-3.83918	-0.20335
H96	1.64092	-3.63127	3.91512
H97	0.29539	-2.89495	3.04075
H98	0.31486	-4.63877	3.31385
H99	-4.09834	-3.51132	2.46583
H100	-5.22573	-4.55195	3.34057
H101	-3.55968	-5.08281	3.06759
H102	-2.56255	8.78133	-0.72349
H103	9.91891	1.26833	0.03849
C104	-7.72916	-6.46649	-0.74549
C105	-7.96477	-7.33151	-1.79389
S106	-9.24963	-5.83759	-0.12837
C107	-9.3456	-7.49723	-2.09577
H108	-7.16852	-7.84793	-2.31954
C109	-10.16093	-6.76199	-1.27984
H110	-9.71559	-8.1437	-2.88408
H111	-11.24095	-6.70331	-1.28355
C112	-4.82908	1.00199	0.18352
C113	-3.79324	0.388	-0.48724
S114	-6.0057	-0.19485	0.70245
C115	-3.93059	-1.02678	-0.57539
H116	-2.94695	0.93729	-0.88609
C117	-5.06873	-1.49043	0.02613
H118	-3.20742	-1.67441	-1.05885
H119	-5.41881	-2.51124	0.10583
C120	-6.46746	-6.06139	-0.16475
C121	-5.03165	2.41035	0.46483

Final geometry:

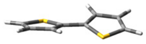

13

Total energy: –1104.81669881 Hartrees

C1	0	3.21057	-0.05036
C2	-1.26892	2.7657	0.19851
C3	-1.35485	1.34567	0.26702
S4	1.11645	1.89317	-0.22313
H5	0.3497	4.22987	-0.14378
H6	-2.11707	3.42706	0.33872
H7	-2.27318	0.80799	0.4783
C8	0.14867	-0.71017	0.06763
C9	1.35485	-1.34567	0.26702
S10	-1.11645	-1.89317	-0.22313
C11	1.26892	-2.7657	0.19851
H12	2.27318	-0.80799	0.4783
C13	0	-3.21057	-0.05036
H14	2.11707	-3.42706	0.33872
H15	-0.3497	-4.22987	-0.14378
C16	-0.14867	0.71017	0.06763

Supporting References

[S1] W. Kabsch, *Acta Crystallogr. D* **2010**, *66*, 125-132.

[S2] Bruker, **2014**, XPREP Version 2014/2, Bruker AXS Inc., Madison.

[S3] G. M. Sheldrick, *Acta Crystallogr. A* **2008**, *64*, 112-122.

[S4] N. Auerhammer, A. Schulz, A. Schmiedel, M. Holzapfel, J. Hoche, M. I. S. Röhr, R. Mitric, C. Lambert, *Phys. Chem. Chem. Phys.* **2019**, *21*, 9013-9025.

[S5] Gaussian 16, Revision B.01, M. J. Frisch, G. W. Trucks, H. B. Schlegel, G. E. Scuseria, M. A. Robb, J. R. Cheeseman, G. Scalmani, V. Barone, G. A. Petersson, H. Nakatsuji, X. Li, M. Caricato, A. V. Marenich, J. Bloino, B. G. Janesko, R. Gomperts, B. Mennucci, H. P. Hratchian, J. V. Ortiz, A. F. Izmaylov, J. L. Sonnenberg, D. Williams-Young, F. Ding, F. Lipparini, F. Egidi, J. Goings, B. Peng, A. Petrone, T. Henderson, D. Ranasinghe, V. G. Zakrzewski, J. Gao, N. Rega, G. Zheng, W. Liang, M. Hada, M. Ehara, K. Toyota, R. Fukuda, J. Hasegawa, M. Ishida, T. Nakajima, Y. Honda, O. Kitao, H. Nakai, T. Vreven, K. Throssell, J. A. Montgomery, Jr., J. E. Peralta, F. Ogliaro, M. J. Bearpark, J. J. Heyd, E. N. Brothers, K. N. Kudin, V. N. Staroverov, T. A. Keith, R. Kobayashi, J. Normand, K. Raghavachari, A. P. Rendell, J. C. Burant, S. S. Iyengar, J. Tomasi, M. Cossi, J. M. Millam, M. Klene, C. Adamo, R. Cammi, J. W. Ochterski, R. L. Martin, K. Morokuma, O. Farkas, J. B. Foresman, and D. J. Fox, Gaussian, Inc., Wallingford CT, 2016.

[S6] M.-A. Sato, K. Fukui, *Synth. Met.* **2007**, *157*, 619-626.

[S7] W. E. Benjamin, D. R. Veit, M. J. Perkins, E. Bain, K. Scharnhorst, S. McDowall, D. L. Patrick, J. D. Gilbertson, *Chem. Mater.* **2014**, *26*, 1291-1293.

[S8] K. Bold, M. Stolte, F. Würthner, *Organic Materials* **2021**, *03*, 119-127.

[S9] For a similar half-life time determination of two rotamers see: F. Yang, C. Liu, D. Yin, Y. Xu, M. Wu, W. Wei, *Chem. Commun.* **2019**, *55*, 14335-14338.

[S10] For a similar determination of strain energies see: M. Ball, B. Fowler, P. Li, L. A. Joyce, F. Li, T. Liu, D. Paley, Y. Zhong, H. Li, S. Xiao, F. Ng, M. L. Steigerwald, C. Nuckolls, *J. Am. Chem. Soc.* **2015**, *137*, 9982-9987.

[S11] A. M. Brouwer, *Pure Appl. Chem.* **2011**, *83*, 2213-2228.
